# The hyperbolic Anderson model: moment estimates of the Malliavin derivatives and applications

**DOI:** 10.1007/s40072-021-00227-5

**Published:** 2022-01-18

**Authors:** Raluca M. Balan, David Nualart, Lluís Quer-Sardanyons, Guangqu Zheng

**Affiliations:** 1 Department of Mathematics and Statistics, STEM Building, University of Ottawa, 150 Louis-Pasteur Private, Ottawa, ON K1N 6N5 Canada; 2 Department of Mathematics, University of Kansas, 405 Snow Hall, Lawrence, KS 66045 USA; 3 Departament de Matemàtiques, Universitat Autònoma de Barcelona, 08193 Cerdanyola del Vallès Catalonia, Spain; 4 School of Mathematics, The University of Edinburgh, James Clerk Maxwell Building, Peter Guthrie Tait Road, Edinburgh, EH9 3FD UK

**Keywords:** Hyperbolic Anderson model, Wiener chaos expansion, Malliavin calculus, Second-order Poincaré inequality, Quantitative central limit theorem, Riesz kernel, Dalang’s condition, 60H15, 60H07, 60G15, 60F05

## Abstract

In this article, we study the hyperbolic Anderson model driven by a space-time *colored* Gaussian homogeneous noise with spatial dimension $d=1,2$. Under mild assumptions, we provide $L^p$-estimates of the iterated Malliavin derivative of the solution in terms of the fundamental solution of the wave solution. To achieve this goal, we rely heavily on the *Wiener chaos expansion* of the solution. Our first application are *quantitative central limit theorems* for spatial averages of the solution to the hyperbolic Anderson model, where the rates of convergence are described by the total variation distance. These quantitative results have been elusive so far due to the temporal correlation of the noise blocking us from using the Itô calculus. A *novel* ingredient to overcome this difficulty is the *second-order Gaussian Poincaré inequality* coupled with the application of the aforementioned $L^p$-estimates of the first two Malliavin derivatives. Besides, we provide the corresponding functional central limit theorems. As a second application, we establish the absolute continuity of the law for the hyperbolic Anderson model. The $L^p$-estimates of Malliavin derivatives are crucial ingredients to verify a local version of Bouleau-Hirsch criterion for absolute continuity. Our approach substantially simplifies the arguments for the one-dimensional case, which has been studied in the recent work by [2].

## Introduction

One of the main tools of modern stochastic analysis is Malliavin calculus. To put it short, this is a differential calculus on a Gaussian space that represents an infinite dimensional generalization of the usual analytical concepts on an Euclidean space. The Malliavin calculus (also known as the stochastic calculus of variations) was initiated by Paul Malliavin [21] to give a probabilistic proof of Hörmander’s “sum of squares” theorem. It has been further developed by Stroock, Bismut, Watanabe and others. One of the main applications of Malliavin calculus is the study of regularity properties of probability laws, for example, the laws of the solutions to certain stochastic differential equations and stochastic partial differential equations (SPDEs), see *e.g.* [27, Chapter 2]. The Malliavin calculus is also useful in formulating and interpreting stochastic (partial) differential equations when the solution is not adapted to a Brownian filtration, which is the case of SPDEs driven by a Gaussian noise that is colored in time.

Recently, the Malliavin calculus has found another important application in the work of Nualart and Ortiz-Latorre [28], which paved the road for *Stein to meet Malliavin*. The authors of [28] applied the Malliavin calculus (notably the integration by parts formula) to characterize the convergence in law of a sequence of multiple Wiener integrals, and they were able to give new proofs for the fourth moment theorems of Nualart, Peccati and Tudor [30, 37]. Soon after the work [28], Nourdin and Peccati combined Malliavin calculus and Stein’s method of normal approximation to quantify the fourth moment theorem. Their work [24] marked the birth of the so-called Malliavin-Stein approach. This combination works admirably well, partially because one of the fundamental ingredients in Stein’s method—the so-called Stein’s lemma (2.6)—that characterizes the normal distribution, is nothing else but a particular case of the integration by parts formula (2.5) in Malliavin calculus. We refer interested readers to [44, Section 1.2] for a friendly introduction to this approach.

The central object of study in this paper is the stochastic wave equation with *linear* Gaussian multiplicative noise (in *Skorokhod sense*):

1.1 $$\begin{aligned} {\left\{ \begin{array}{ll} \dfrac{\partial ^2 u}{\partial t^2} =\Delta u+u{\dot{W}} \\ u(0,x) = 1, \quad \dfrac{\partial u}{\partial t}(0,x)=0\end{array}\right. } ~\text {on }{\mathbb {R}}_{+} \times {\mathbb {R}}^d\text { for }d\in \{1,2\}, \end{aligned}$$

where $\Delta$ is the Laplacian in space variables and the Gaussian noise ${\dot{W}}$ has the following correlation structure

$$\begin{aligned} {\mathbb {E}}\big [ {\dot{W}}(t,x) {\dot{W}}(s,y) \big ] =\gamma _0(t-s) \gamma (x-y), \end{aligned}$$

with the following standing assumptions:

(i) $\gamma _0:{\mathbb {R}}\rightarrow [0,\infty ]$ is locally integrable and non-negative definite;
(ii) $\gamma$ is a non-negative and non-negative definite measure on ${\mathbb {R}}^d$ whose spectral measure $\mu$^1^ satisfies *Dalang’s condition*:
  1.2 $$\begin{aligned} \qquad \qquad \quad \int _{{\mathbb {R}}^d}\frac{1}{1+|\xi |^2}\mu (d\xi )<\infty , \end{aligned}$$

where $|\xi |$ denotes the Euclidean norm of $\xi \in {\mathbb {R}}^d$.

An important example of the temporal correlation is the Riesz kernel $\gamma _0(t)=|t|^{-\alpha _0}$ for some $\alpha _0\in (0,1)$ (with $\gamma _0(0)=\infty$).

Equation (1.1) is also known in the literature as the *hyperbolic Anderson model*, by analogy with the parabolic Anderson model in which the wave operator is replaced by the heat operator. The noise ${\dot{W}}$ can be formally realized as an isonormal Gaussian process $W=\{W(\phi ): \phi \in {\mathcal {H}}\}$ and here ${\mathcal {H}}$ is a Hilbert space that is the completion of the set $C^\infty _c\big ({\mathbb {R}}_+\times {\mathbb {R}}^d)$ of infinitely differentiable functions with compact support under the inner product

1.3 $$\begin{aligned} \langle \phi , \psi \rangle _{{\mathcal {H}}}&= \int _{{\mathbb {R}}_{+}^2 \times {\mathbb {R}}^{2d}}\phi (t,x)\psi (s,y)\gamma _0(t-s)\gamma (x-y)dtdxdsdy \end{aligned}$$

1.4 $$\begin{aligned}&= \int _{{\mathbb {R}}_{+}^2 } dt ds \gamma _0(t-s) \int _{{\mathbb {R}}^d} dx \phi (t,x) \big [\psi (s,\bullet ) *\gamma \big ](x) , \end{aligned}$$

where we write $\gamma (x)$ for the density of $\gamma$ if it exists and we shall use the definition (1.4) instead of (1.3) when $\gamma$ is a measure. In (1.4), $*$ denotes the convolution in the space variable and $\gamma _0(t)= \gamma _0(-t)$ for $t<0$. We denote by ${\mathcal {H}}^{\otimes p}$ the *p*th tensor product of ${\mathcal {H}}$ for $p\in {\mathbb {N}}^*$, see Sect. 2 for more details.

As mentioned before, the existence of a temporal correlation $\gamma _0$ prevents us from defining equation (1.1) in the Itô sense due to a lack of the martingale structure. In the recent work [3] by Balan and Song, the following results are established using Malliavin calculus. Let $G_t$ denote the fundamental solution to the corresponding deterministic wave equation, that is, for $(t,z)\in (0,\infty )\times {\mathbb {R}}^d$,

1.5 $$\begin{aligned} G_t(z) := {\left\{ \begin{array}{ll} \dfrac{1}{2} {\mathbf {1}}_{\{ | z|< t\}} \quad &{}\text {if }d=1; \\ \dfrac{1}{2\pi \sqrt{ t^2 - | z|^2}} {\mathbf {1}}_{\{ | z| < t\}} \quad &{}\text {if }d=2. \end{array}\right. } \end{aligned}$$

To ease the notation, we will stick to the convention that

1.6 $$\begin{aligned} G_t(z) =0\text { when }t\le 0. \end{aligned}$$

### Definition 1.1

Fix $$d\in \{1,2\}$$. We say that a square-integrable process $$u = \{ u(t,x): (t,x)\in {\mathbb {R}}_+\times {\mathbb {R}}^d\}$$ is a *mild Skorokhod solution* to the hyperbolic Anderson model (1.1) if *u* has a jointly measurable modification (still denoted by $$u$$ $$)$$ such that $$\sup \{ {\mathbb {E}}[u(t,x)^2 ]: (t,x)\in [0,T]\times {\mathbb {R}}^d\} < \infty $$ for any finite *T*; and for any $$t>0$$ and $$x\in {\mathbb {R}}^d$$, the following equality holds in $$L^2(\Omega )$$:

$$\begin{aligned} u(t,x)=1 + \int _0^t \int _{{\mathbb {R}}^d} G_{t-s}(x-y) u(s,y) W(ds,dy), \end{aligned}$$

where the above stochastic integral is understood in the *Skorokhod sense* and the process $(s,y)\in {\mathbb {R}}_+\times {\mathbb {R}}^d\longmapsto {\mathbf {1}}_{(0,t)}(s) G_{t-s}(x-y)u(s,y)$ is Skorokhod integrable. See Definition 5.1 in [3] and Definition 1.1 in [2].

It has been proved in [3, Section 5] that equation (1.1) admits a unique mild Skorokhod solution *u* with the following Wiener chaos expansion:

1.7 $$\begin{aligned} u(t,x) = 1 + \sum _{n\ge 1}I_n\big ( {\widetilde{f}}_{t,x,n} \big ), \end{aligned}$$

where $I_n$ denotes the *n*th multiple Wiener integral associated to the isonormal Gaussian process *W* (see Sect. 2 for more details), $f_{t,x,n}\in {\mathcal {H}}^{\otimes n}$ is defined by (with the convention (1.6) in mind)

1.8 $$\begin{aligned} f_{t,x,n}(t_1,x_1,\dots ,t_n, x_n)\!:=\!G_{t-t_{1}}(x-x_{1}) G_{t_1-t_2}(x_1-x_2) \cdots G_{t_{n-1}-t_n}(x_{n-1}\!-\!x_n), \nonumber \\ \end{aligned}$$

and ${\widetilde{f}}_{t,x,n}$ is the canonical symmetrization of $f_{t,x,n}\in {\mathcal {H}}^{\otimes n}$ given by

1.9 $$\begin{aligned} {\widetilde{f}}_{t,x,n}(t_1,x_1,\dots ,t_n, x_n):=\frac{1}{n!} \sum _{\sigma \in {\mathfrak {S}}_n}f_{t,x,n}(t_{\sigma (1)}, x_{\sigma (1)}, \dots ,t_{\sigma (n)}, x_{\sigma (n)}), \end{aligned}$$

where the sum in (1.9) runs over ${\mathfrak {S}}_n$, the set of permutations on $\{1,2,\dots , n\}$. For example, $f_{t,x,1}(t_1,x_1) =G_{t-t_1}(x-x_1)$ and

$$\begin{aligned}&{\widetilde{f}}_{t,x,2}(t_1,x_1, t_2, x_2)\\&\quad = \frac{1}{2} \Big ( G_{t-t_1}(x-x_1) G_{t_1-t_2}(x_1\!-\!x_2) \!+\! G_{t-t_2}(x-x_2) G_{t_2-t_1}(x_2-x_1) \Big ). \end{aligned}$$

We would like to point out that in the presence of temporal correlation, there is no developed solution theory for the nonlinear wave equation (replacing $u {\dot{W}}$ in (1.1) by $\sigma (u) {\dot{W}}$ for some deterministic Lipschitz function $\sigma :{\mathbb {R}}\rightarrow {\mathbb {R}}$). We regard this as a totally different problem.

Now let us introduce the following hypothesis when $d=2$:

$$\begin{aligned} \mathbf{(H1)} {\left\{ \begin{array}{ll} &{}{(\texttt {a}) \gamma \in L^\ell ({\mathbb {R}}^2)\text { for some }\ell \in (1,\infty ),}\\ &{} {(\texttt {b}) \gamma (x)= |x|^{-\beta }\text { for some } \beta \in (0,2),}\\ &{}{(\texttt {c}) \gamma (x_1,x_2) =\gamma _1(x_1)\gamma _2(x_2),\text { where }\gamma _i(x_i) = |x_i|^{-\beta _i}\text { or }\gamma _i\in L^{\ell _i}({\mathbb {R}}) } \\ &{}\qquad \text {for some }0< \beta _i< 1< \ell _i <+\infty , i=1,2. \end{array}\right. } \end{aligned}$$

### Remark 1.2

(i) Note that condition (a) for $d=2$ is slightly stronger than Dalang’s condition (1.2). In fact, when $d=2$, the paper [18] pointed out that Dalang’s condition (1.2) is equivalent to
  1.10 $$\begin{aligned} \int _{|x|\le 1} \ln ( |x|^{-1} ) \gamma (x)dx < \infty ; \end{aligned}$$
  let $\ell ^\star = \frac{\ell }{\ell -1}$ and $0< \varepsilon < 1/\ell ^\star$, then there is some $\delta \in (0,1)$ and a constant $C_\varepsilon$ such that $\ln ( |x|^{-1}) \le C_\varepsilon |x|^{-\varepsilon }$ for any $|x|\le \delta$, from which we deduce that
  $$\begin{aligned} \int _{|x|\le 1} \ln ( |x|^{-1} ) \gamma (x)dx&\le \ln (\delta ^{-1}) \int _{ \delta< |x|\le 1} \gamma (x)dx + C_\varepsilon \int _{|x|\le \delta } |x|^{-\varepsilon } \gamma (x)dx \\&\le \ln (\delta ^{-1}) \int _{ \delta< |x|\le 1} \gamma (x)dx \\&\quad + C_\varepsilon \Vert \gamma \Vert _{L^\ell ({\mathbb {R}}^2)}\left( \int _{|x|\le \delta } |x|^{-\varepsilon \ell ^\star }dx \right) ^{1/\ell ^\star }<\infty . \end{aligned}$$
(ii) The case (c) in Hypothesis $\mathbf{(H1)}$ is a mixture of cases (a) and (b). Accordingly, more examples of the noise ${\dot{W}}$ arise. In the space variables, *W* can behave like a fractional Brownian sheet with Hurst indices greater than 1/2 in both directions, i.e. $\gamma (x_1,x_2)=|x_1|^{2H_1-2}|x_2|^{2H_2-2}$ for some $H_1,H_2 \in (1/2,1)$.
(iii) For $d=1$ we just assume that $\gamma$ is a non-negative and non-negative definite measure on ${\mathbb {R}}$. In this case (see, for instance, Remark 10 of [11]) Dalang’s condition is always satisfied.

Under Hypothesis $\mathbf (H1)$, we will state our first main result — the $L^p(\Omega )$ estimates of the Malliavin derivatives of *u*(*t*, *x*). The first Malliavin derivative *Du*(*t*, *x*) is a random element in the Hilbert space ${\mathcal {H}}$, the completion of $C^\infty _c\big ({\mathbb {R}}_+\times {\mathbb {R}}^d)$ under the inner product (1.3); as the space ${\mathcal {H}}$ contains generalized functions, it is not clear at first sight whether $(s,y) \longmapsto D_{s,y}u(t,x)$ is a (random) function. The higher-order Malliavin derivative $D^{m} u(t,x)$ is a random element in ${\mathcal {H}}^{\otimes m}$ for $m\ge 1$, see Sect. 2 for more details.

Let us first fix some notation.

**Notation A** (1) We write $a\lesssim b$ to mean $a\le Kb$ for some immaterial constant $K>0$.

(2) We write $\Vert X\Vert _p = \big ({\mathbb {E}}[ |X | ^p ]\big )^{1/p}$ to denote the $L^p(\Omega )$-norm of *X* for $p\in [1,\infty )$.

(3) When *p* is a positive integer, we often write $\pmb {z_p} = (z_1, \dots , z_p)$ for points in ${\mathbb {R}}_+^p$ or ${\mathbb {R}}^{dp}$, and $d\pmb {z_p}=dz_1 \cdots dz_p$, $\mu (d\pmb {z_p}) = \mu (dz_1)\cdots \mu (dz_p)$. For a function $h: ({\mathbb {R}}_+\times {\mathbb {R}}^d)^p\rightarrow {\mathbb {R}}$ with $p\ge 2$, we often write

$$\begin{aligned} h(\pmb {s_p}, \pmb {y_p}) = h(s_1, \dots , s_p, y_1,\dots , y_p) = h(s_1, y_1, \dots , s_p, y_p), \end{aligned}$$

which shall not cause any confusion. For $m\in \{1,\dots , p-1\}$ and $(\pmb {s_m}, \pmb {y_m})\in {\mathbb {R}}_+^m\times {\mathbb {R}}^{dm}$, the expression $h(\pmb {s_m}, \pmb {y_m};\bullet )$ stands for the function

$$\begin{aligned}&(t_1,x_1, \dots , t_{p-m}, x_{p-m}) \mapsto h(s_1, y_1, \dots , s_m, y_m, t_1, x_1, \dots , t_{p-m}, x_{p-m})\\&\quad =h(\pmb {s_m}, \pmb {y_m};\pmb {t_{p-m}}, \pmb {x_{p-m}}). \end{aligned}$$

Now, with the above notation in mind, we are in the position to state the first main result.^2^

### Theorem 1.3

Let $d\in \{1,2\}$ and suppose that Hypothesis $\mathbf (H1)$ holds if $d=2$. Then, for any $(t,x) \in {\mathbb {R}}_+\times {\mathbb {R}}^d$, the random variable *u*(*t*, *x*) belongs to ${\mathbb {D}}^{\infty }$ (see Sect. 2.1). Moreover, for any integer $m\ge 1$, the *m*th Malliavin derivative $D^mu(t,x)$ is a random symmetric function denoted by

$$\begin{aligned} (\pmb {s_m}, \pmb {y_m})\!=\!(s_1,y_1, \dots , s_m, y_m)\longmapsto D_{s_1,y_1} D_{s_2, y_2}\ldots D_{s_m, y_m} u(t,x) \!=\! D^m_{\pmb {s_m}, \pmb {y_m}} u(t,x), \end{aligned}$$

and for any $p\in [2,\infty )$, we have, for almost all $(\pmb {s_m}, \pmb {y_m}) \in [0,t]^m \times {\mathbb {R}}^{md}$,

1.11 $$\begin{aligned} m! {\widetilde{f}}_{t,x,m}(\pmb {s_m}, \pmb {y_m}) \le \big \Vert D^m_{\pmb {s_m}, \pmb {y_m}} u(t,x) \big \Vert _p \lesssim {\widetilde{f}}_{t,x,m}(\pmb {s_m}, \pmb {y_m}), \end{aligned}$$

where the constant in the upper bound only depends on $(p,t,\gamma _0, \gamma ,m)$ and is increasing in *t*. Moreover, $D^m u(t,x)$ has a measurable modification.

Throughout this paper, we will work with the measurable modifications of *Du*(*t*, *x*) and $D^2 u(t,x)$ given by Theorem 1.3, which are still denoted by $D u(t,x), D^2 u(t,x)$ respectively.

In this paper, we will present two applications of Theorem 1.3. Our first application are *quantitative central limit theorems* (CLTs) for the spatial averages of the solution to (1.1), which have been elusive so far due to the temporal correlation of the noise preventing the use of Itô calculus approach. A *novel* ingredient to overcome this difficulty is the so-called *second-order Gaussian Poincaré inequality* in an improved form. We will address these CLT results in Sect. 1.1. While in Sect. 1.2, as the second application, we establish the absolute continuity of the law of the solution to equation (1.1) using the $L^p$-estimates of Malliavin derivatives that are crucial to establish a local version of Bouleau-Hirsch criterion [5].

### Gaussian fluctuation of spatial averages

Spatial averages of SPDEs have recently attracted considerable interest. It was Huang, Nualart and Viitasaari who first studied the fluctuation of spatial statistics and established a central limit theorem for a nonlinear SPDE in [15]. More precisely, they considered the following one-dimensional stochastic heat equation

1.12 $$\begin{aligned} \frac{\partial u}{\partial t} = \frac{1}{2}\Delta u + \sigma (u) {\dot{W}} \end{aligned}$$

on ${\mathbb {R}}_+\times {\mathbb {R}}$, where ${\dot{W}}$ is a space-time Gaussian white noise, with constant initial condition $u(0,\bullet )=1$ and the nonlinearity $\sigma :{\mathbb {R}}\rightarrow {\mathbb {R}}$ is a Lipschitz function. In view of the localization property of its mild formulation (in the Walsh sense [43]),

1.13 $$\begin{aligned} u(t,x)=1+\int _0^t \int _{{\mathbb {R}}} p_{t-s}(x-y) \sigma \big ( u(s,y) \big ) W(ds, dy), \end{aligned}$$

with $p_t$ denoting the heat kernel,^3^ one can regard *u*(*t*, *x*) and *u*(*t*, *y*) as weakly dependent random variables for *x*, *y* far apart so that the integral

$$\begin{aligned} \int _{-R}^R \big [ u(t,x) -1 \big ] dx \end{aligned}$$

can be roughly understood as a sum of weakly dependent random variables. Therefore, it is very natural to expect Gaussian fluctuations when *R* tends to infinity.

Let us stop now to briefly fix some notation to facilitate our discussion.

**Notation B.** (1) For $t>0$, we define, with $B_R:=\{ x\in {\mathbb {R}}^d: |x| \le R\}$,

1.14 $$\begin{aligned} F_R(t) := \int _{B_R} \big [ u(t,x) -1 \big ] dx \quad \mathrm{and}\quad \sigma _R(t) = \sqrt{ \text {Var}\big (F_R(t) \big ) }. \end{aligned}$$

(2) We write $f(R)\sim g(R)$ to mean that *f*(*R*)/*g*(*R*) converges to some positive constant as $R\rightarrow \infty$.

(3) For two real random variables *X*, *Y* with distribution measures $\mu , \nu$ respectively, the total variation distance between *X*, *Y* (or $\mu ,\nu$) is defined to be

1.15 $$\begin{aligned} d_{\mathrm{TV}}( X, Y) = \sup _{B} \big \vert \mu (B) -\nu (B)\vert , \end{aligned}$$

where the supremum runs over all Borel set $B\subset {\mathbb {R}}$. The total variation distance is well known to induce a stronger topology than that of convergence in distribution, see [25, Appendix C].

(4) We define the following quantities for future reference:

1.16 $$\begin{aligned} \omega _1=2, \quad \omega _2=\pi , \quad \mathrm{and}\quad \kappa _{\beta ,d} := \int _{{\mathbb {R}}^{2d}} dxdy |x-y|^{-\beta } {\mathbf {1}}_{B_1}(x) {\mathbf {1}}_{B_1}(y)~\text {for }\beta \in (0,d). \end{aligned}$$

(5) For an integer $m\ge 1$ and $p\in [1,\infty )$, we say $F\in {\mathbb {D}}^{m,p}$ if *F* is *m*-times Malliavin differentiable random variable in $L^p(\Omega )$ and ${\mathbb {E}}\big [ \Vert D^j F\Vert _{{\mathcal {H}}^{\otimes j}}^p \big ] <\infty$ for every $j=1,\dots , m$; see Sect. 2.1 for more details.

Now let us illustrate the strategy in [15]: (For this reference, $d=1$)

- The authors first rewrite $F_R(t) = \delta (V_{t, R})$ with the random kernel
  $$\begin{aligned} V_{t,R}(s,y) = \sigma (u(s,y) ) \int _{B_R} p_{t-s}(x-y) dx, \end{aligned}$$
  where $\delta$ denotes the Skorokhod integral, the adjoint of the Malliavin derivative *D*.
- By standard computations, they obtained $\sigma ^2_R(t)\sim R$.
- If $F=\delta (v)\in {\mathbb {D}}^{1,2}$ is a centered random variable with variance one, for some *v* in the domain of $\delta$, the (univariate) Malliavin-Stein bound (see [15, Proposition 2.2]) ensures that $d_{\mathrm{TV}}( F, Z )\le 2\sqrt{ \text {Var}( \langle DF, v\rangle _{\mathcal {H}})}$ for $Z\sim N(0,1)$.
- Combining the above points, one can see that the obtention of a quantitative CLT is reduced to the computation of $\text {Var}( \langle DF_R(t), V_{t,R} \rangle _{\mathcal {H}})$.

Because the driving noise is white in time as considered in [15], tools from Itô calculus (Clark-Ocone formula, Burkholder’s inequality, *etc.*) are used to estimate the above variance term. It is proved in [15] that $d_{\mathrm{TV}}( F_R(t) /\sigma _R(t), Z ) \lesssim R^{-1/2}$. Meanwhile, a multivariate Malliavin-Stein bound and similar computations lead to the convergence of the finite-dimensional distributions, which coupled with the tightness property gives a functional CLT for $\{ R^{-1/2}F_R(t): t\in {\mathbb {R}}_+\}$.

The above general strategy has been adapted to various settings, see [9, 10, 16, 19, 20, 38] for the study of stochastic heat equations and see [4, 12, 35] for the study of stochastic wave equations. All these references consider a Gaussian noise that is white in time. Nevertheless, when the Gaussian noise is colored in time, the mild formulation (1.13) cannot be interpreted in the Walsh-Itô sense. In this situation, only in the case $\sigma (u)=u$ the stochastic heat equation (1.12) (also known as the *parabolic Anderson model*) can be properly solved using Wiener chaos expansions, so that $F_R(t)$, defined in (1.14), can be expressed as an infinite sum of multiple Wiener integrals. With this well-known fact in mind, Nualart and Zheng [33] considered the parabolic Anderson model (*i.e.* (1.12) with $\sigma (u)=u$) on ${\mathbb {R}}_+\times {\mathbb {R}}^d$ such that $d\ge 1$, the initial condition is constant and the assumptions (i)–(ii) hold (see page 2). The main result of [33] is the chaotic CLT that is based on the fourth moment theorems [30, 37]. When, additionally, $\gamma$ is a finite measure, the authors of [33] established $\sigma _R(t)\sim R^{d/2}$ and a functional CLT for the process $R^{-d/2} F_R$; they also considered the case where $\gamma (x)=|x|^{-\beta }$, for some $\beta \in (0,2\wedge d)$, is the Riesz kernel, and obtain the corresponding CLT results. As pointed out in the paper [33], due to the homogeneity of the underlying Gaussian noise, the solution *u* to (1.12) can be regarded as the functional of a stationary Gaussian random field so that, with the Breuer-Major theorem [6] in mind, it is natural to study Gaussian fluctuations for the problems (1.12) and (1.1). Note that the constant initial condition makes the solution stationary in space and, in fact it is spatially ergodic (see [10, 36]). At last, let us mention the paper [32] in which chaotic CLT was used to study the parabolic Anderson model driven by a colored Gaussian noise that is rough in space. However, let us point out that the aforementioned methods fail to provide the rate of convergence when the noise is colored in time.

In this paper, we bring in a novel ingredient—the *second-order Gaussian Poincaré inequality*^4^—to reach quantitative CLT results for the hyperbolic Anderson model (1.1). Let us first state our main result.

#### Theorem 1.4

Let *u* denote the solution to the hyperbolic Anderson model (1.1) and recall the definition of $F_R(t)$ and $\sigma _R(t)$ from (1.14). Let $Z\sim N(0,1)$ be the standard normal random variable. We assume that $\gamma _0$ is not identically zero meaning

1.17 $$\begin{aligned} \Vert \gamma _0\Vert _{L^1([0,\varepsilon ])}>0 ~\text { for any }\varepsilon \in (0,1). \end{aligned}$$

Then the following statements hold true:

1. Suppose that $0<\gamma ({\mathbb {R}}^d) <\infty$ if $d=1$ and $\gamma \in L^1({\mathbb {R}}^d) \cap L^\ell ({\mathbb {R}}^d)$ for some $\ell >1$ if $d=2$. Then,
  $$\begin{aligned} \sigma _R(t) \sim R^{d/2}\text { and } d_{\mathrm{TV}}\big ( F_R(t) / \sigma _R(t) , Z \big ) \lesssim R^{-d/2}. \end{aligned}$$
  Moreover, as $R\rightarrow \infty$, the process $\big \{ R^{-d/2} F_R(t): t\in {\mathbb {R}}_+\big \}$ converges weakly in the space of continuous functions $C({\mathbb {R}}_+)$ to a centered Gaussian process ${\mathcal {G}}$ with covariance structure
  1.18 $$\begin{aligned} {\mathbb {E}}\big [ {\mathcal {G}}(t) {\mathcal {G}}(s) \big ] = \omega _d \sum _{p\ge 1} p! \int _{{\mathbb {R}}^d} \big \langle {\widetilde{f}}_{t,x,p}, {\widetilde{f}}_{s,0,p} \big \rangle _{{\mathcal {H}}^{\otimes p}}dx, \end{aligned}$$
  for $t,s\in {\mathbb {R}}_+$. Here $\omega _1=2$, $\omega _2=\pi$ and ${\widetilde{f}}_{t,x,p}$ are introduced in (1.16) and (1.9), respectively. The convergence of the series in (1.18) is part of the conclusion.
2. Suppose $d\in \{1,2\}$ and $\gamma (x) = | x|^{-\beta }$ for some $\beta \in (0,2\wedge d)$. Then,
  $$\begin{aligned} \sigma _R(t) \sim R^{d-\frac{\beta }{2}}\text { and } d_{\mathrm{TV}}\big ( F_R(t) / \sigma _R(t) , Z \big ) \lesssim R^{-\beta /2}. \end{aligned}$$
  Moreover, as $R\rightarrow \infty$, the process $\big \{ R^{-d+\frac{\beta }{2}} F_R(t): t\in {\mathbb {R}}_+\big \}$ converges weakly in the space $C({\mathbb {R}}_+)$ to a centered Gaussian process ${\mathcal {G}}_{\beta }$ with the covariance structure
  1.19 $$\begin{aligned} {\mathbb {E}}\big [ {\mathcal {G}}_\beta (t) {\mathcal {G}}_\beta (s) \big ] = \kappa _{\beta , d} \int _0^t dr\int _0^s dr' \gamma _0(r-r') (t-r)(s-r'), \end{aligned}$$
  for $t,s\in {\mathbb {R}}_+$. Here the quantity $\kappa _{\beta , d}$ is introduced in (1.16).
3. Suppose $d=2$ and $\gamma (x_1,x_2) = \gamma _1(x_1) \gamma _2(x_2)$ such that one of the following two conditions holds:
  1.20 $$\begin{aligned} {\left\{ \begin{array}{ll} &{}\mathrm{(a')} ~\gamma _i(x_i) =|x_i|^{-\beta _i}~\text {for some }\beta _i\!\in (0,1), i=1,2; \\ &{}\mathrm{(b')} ~\gamma _1\!\in L^{\ell }({\mathbb {R}}) \cap L^1({\mathbb {R}}) ~\text {and }\gamma _2(x_2)=|x_2|^{-\beta }\text { for some }0\!<\! \beta< 1 \!<\! \ell <\infty . \end{array}\right. } \end{aligned}$$
  Then,
  $$\begin{aligned} {\left\{ \begin{array}{ll} \sigma _R(t) \sim R^{2 - \frac{1}{2}( \beta _1 + \beta _2)} \quad \text {and} \quad d_{\mathrm{TV}}\big ( F_R(t) / \sigma _R(t) , Z \big ) \!\lesssim \! R^{-(\beta _1\!+\!\beta _2)/2} ~ &{} \text {in case }\mathrm{(a')}, \\ \sigma _R(t) \!\sim R^{(3-\beta )/2 } \quad \text {and} \quad d_{\mathrm{TV}}\big ( F_R(t) / \sigma _R(t) , Z \big ) \!\lesssim \! R^{-(\beta +1)/2} ~ &{} \text {in case }\mathrm{(b')}. \end{array}\right. } \end{aligned}$$
  Moreover, as $R\rightarrow \infty$, in case $(a')$ , the process $\big \{ R^{-2+\frac{\beta _1+\beta _2}{2}} F_R(t): t\in {\mathbb {R}}_+\big \}$ converges weakly in the space $C({\mathbb {R}}_+)$ to a centered Gaussian process ${\mathcal {G}}_{\beta _1, \beta _2}$ with the covariance structure
  1.21 $$\begin{aligned} {\mathbb {E}}\big [ {\mathcal {G}}_{\beta _1, \beta _2}(t) {\mathcal {G}}_{\beta _1, \beta _2 } (s) \big ] = K_{\beta _1, \beta _2} \int _0^t dr\int _0^s dr' \gamma _0(r-r') (t-r)(s-r'), \end{aligned}$$
  for $$t,s\in {\mathbb {R}}_+$$, where
  1.22 $$\begin{aligned} K_{\beta _1, \beta _2} :&= \int _{{\mathbb {R}}^4} {\mathbf {1}}_{\{ x_1^2+x_2^2\le 1 \}} {\mathbf {1}}_{\{ y_1^2+y_2^2\le 1 \}} |x_1 - y_1|^{-\beta _1} |x_2 - y_2|^{-\beta _2} dx_1dx_2dy_1dy_2; \end{aligned}$$
  and in case $(b')$ , the process $\big \{ R^{\frac{\beta -3}{2}} F_R(t): t\in {\mathbb {R}}_+\big \}$ converges weakly in the space $C({\mathbb {R}}_+)$ to a centered Gaussian process $\widehat{{\mathcal {G}}}_{\beta }$ with the covariance structure
  1.23 $$\begin{aligned} {\mathbb {E}}\big [ \widehat{{\mathcal {G}}}_{\beta }(t) \widehat{{\mathcal {G}}}_{\beta } (s) \big ] = \gamma _1({\mathbb {R}}) {\mathcal {L}}_\beta \int _0^t dr\int _0^s dr' \gamma _0(r-r') (t-r)(s-r') \end{aligned}$$
  for $$t,s\in {\mathbb {R}}_+$$, where
  1.24 $$\begin{aligned} {\mathcal {L}}_\beta : = \int _{{\mathbb {R}}^3} dx_1dx_2 dx_3 {\mathbf {1}}_{\{ x_1^2 + x_2^2\le 1 \}} {\mathbf {1}}_{\{ x_1^2 + x_3^2\le 1 \}} |x_2-x_3|^{-\beta }. \end{aligned}$$

For the above functional convergences, we specify that the space $C({\mathbb {R}}_+)$ is equipped with the topology of uniform convergence on compact sets.

#### Remark 1.5

1. Note that the case when $\gamma (x) =\gamma _1(x_1)\gamma _2(x_2)$ with $\gamma _i\in L^{\ell _i}({\mathbb {R}})\cap L^1({\mathbb {R}})$ for some $\ell _i>1$, $i=1,2$, is covered in part (1). Indeed, suppose that $\ell _1\ge \ell _2$, then by Hölder’s inequality, $\gamma _1\in L^{\ell _1}({\mathbb {R}})\cap L^{1}({\mathbb {R}})$ implies $\gamma _1\in L^{\ell _2}({\mathbb {R}})\cap L^{1}({\mathbb {R}})$ and hence $\gamma \in L^{\ell _2}({\mathbb {R}}^2) \cap L^1({\mathbb {R}}^2)$.
2. The rate of convergence can also be described using other common distances such as the Wasserstein distance and the Kolmogorov distance; see [25, Appendix C].
3. The variance orders and the rates in parts (1) and (2) of Theorem 1.4 are consistent with previous work on stochastic wave equations, see [4, 12, 35]. The setting in part (3) is new. As we will see shortly, our strategy is quite different from that in these papers.

Now, let us briefly explain our strategy and begin with the Gaussian Poincaré inequality. For $F\in {\mathbb {D}}^{1,2}$, the Gaussian Poincaré inequality (see *e.g.* [14] or (2.12)) ensures that

$$\begin{aligned} \text {Var}(F) \le {\mathbb {E}}\big [ \Vert DF \Vert _{\mathcal {H}}^2 \big ]~\text {with equality if and only if }F\text { is Gaussian}, \end{aligned}$$

that is, if *DF* is small, then the random variable *F* has necessarily small fluctuations. In the paper [8], Chatterjee pointed out that for $F=f(X_1, \dots , X_d)$ with $X_1, \dots , X_d$ i.i.d. *N*(0, 1) and *f* twice differentiable, *F* is close in total variation distance to a normal distribution with matched mean and variance if the Hessian matrix $\text {Hess}f(X_1, \dots , X_d)$ is negligible, roughly speaking. This is known as the second-order Gaussian Poincaré inequality. In what follows, we state the infinite-dimensional version of this inequality due to Nourdin, Peccati and Reinert; see the paper [26] as well as the book [25].^5^

#### Proposition 1.6

Let *F* be a centered element of ${\mathbb {D}}^{2,4}$ such that ${\mathbb {E}}[ F^2] = \sigma ^2 > 0$ and let $Z\sim N(0,\sigma ^2)$. Then,

1.25 $$\begin{aligned} d_{\mathrm{TV}}(F,Z) \le \frac{3}{\sigma ^2} \left( {\mathbb {E}}\Big [ \big \Vert D^2 F \otimes _1 D^2F \big \Vert ^2_{ {\mathcal {H}}^{\otimes 2}} \Big ] \right) ^{1/4} \left( {\mathbb {E}}\big [ \Vert DF \Vert _{{\mathcal {H}}}^4 \big ]\right) ^{1/4}, \end{aligned}$$

where $D^2 F \otimes _1 D^2F$ denotes the 1-contraction between $D^2F$ and itself (see 2.10).

It has been known that this inequality usually gives sub-optimal rate. In the recent work [42] by Vidotto, she provided an improved version of the above inequality, where she considered an $L^2$-based Hilbert space ${\mathcal {H}}= L^2(A, \nu )$ with $\nu$ a diffusive measure (nonnegative, $\sigma$-finite and non-atomic) on some measurable space *A*. Let us state this result for the convenience of readers.

#### Theorem 1.7

(Theorem 2.1 in [42]) Let $F\in {\mathbb {D}}^{2,4}$ with mean zero and variance $\sigma ^2>0$ and let $Z\sim N(0,\sigma ^2)$. Suppose ${\mathcal {H}}= L^2(A,\nu )$ with $\nu$ a diffusive measure on some measurable space *A*. Then,

$$\begin{aligned}&d_{\mathrm{TV}}\big (F, Z\big ) \\&\, \le \! \frac{4}{\sigma ^2}\!\! \left[ \int _{A\times A} \!\!\sqrt{ {\mathbb {E}}\big [ \big ( D^2F\otimes _1 D^2F\big )^2(x,y) \big ] \!\times \! {\mathbb {E}}\big [ (DF)^2(x) (DF)^2(y) \big ] } \nu (dx)\nu (dy) \right] ^{\frac{1}{2}}\!. \end{aligned}$$

The proof of the above inequality follows from the general Malliavin-Stein bound

1.26 $$\begin{aligned} d_{\mathrm{TV}}\big (F, Z\big ) \le \frac{2}{\sigma ^2} {\mathbb {E}}\left( \big \vert \sigma ^2 - \langle DF, - DL^{-1}F \rangle _{{\mathcal {H}}} \big \vert \right) \end{aligned}$$

(see [25, equation (5.1.4)])^6^ and Vidotto’s new bound of

$$\begin{aligned} \qquad \qquad {\mathbb {E}}\big [ ( \text {Cov}(F,G) - \langle DF, - DL^{-1}G \rangle _{{\mathcal {H}}} )^2 \big ]~\text {for centered }F, G\in {\mathbb {D}}^{2,4} \end{aligned}$$

(see [42, Proposition 3.2]), where $L^{-1}$ is the pseudo-inverse of the Ornstein-Uhlenbeck operator *L*; see Sect. 2.1 for the definitions.

Recall that our Hilbert space ${\mathcal {H}}$ is the completion of $C^\infty _c({\mathbb {R}}_+\times {\mathbb {R}}^d)$ under the inner product (1.3). The Hilbert space ${\mathcal {H}}$ contains generalized functions, but fortunately the objects $D^2u(t,x)$, *Du*(*t*, *x*) are random functions in view of Theorem 1.3. By adapting Vidotto’s proof to our setting, we have the following version of second-order Gaussian Poincaré inequality. Note we write $f\in | {\mathcal {H}}^{\otimes p }|$ to mean *f* is a real valued function and $\bullet \mapsto | f(\bullet ) |$ belongs to ${\mathcal {H}}^{\otimes p }$.

#### Proposition 1.8

If $F\in {\mathbb {D}}^{2,4}$ has mean zero and variance $\sigma ^2\in (0,\infty )$ such that with probability 1, $DF\in | {\mathcal {H}}|$ and $D^2F\in |{\mathcal {H}}^{\otimes 2}|$, then

$$\begin{aligned} d_{\mathrm{TV}}\big ( F, Z\big ) \le \frac{4}{\sigma ^2} \sqrt{{\mathcal {A}}}, \end{aligned}$$

where $$Z\sim N(0,\sigma ^2)$$ and

$$\begin{aligned} {\mathcal {A}}:&= \int _{{\mathbb {R}}_+^6\times {\mathbb {R}}^{6d}} drdr' dsds' d\theta d\theta ' dzdz' dydy' dwdw' \gamma _0(\theta - \theta ') \gamma _0(s-s') \gamma _0(r-r') \\&\quad \times \gamma (z-z') \gamma (w-w') \gamma (y-y') \Vert D_{r,z}D_{\theta ,w}F \Vert _4 \Vert D_{s,y}D_{\theta ',w'}F \Vert _4 \Vert \\&\qquad \times D_{r',z'}F\Vert _4 \Vert D_{s', y' }F \Vert _4. \end{aligned}$$

As mentioned before, Proposition 1.8 will follow from the Malliavin-Stein bound (1.26) and Cauchy-Schwarz inequality, taking into account that, by the duality relation (2.5), we have that ${\mathbb {E}}\left( \langle DF, - DL^{-1}F \rangle _{{\mathcal {H}}} \right) = {\mathbb {E}}[ F^2]=\sigma ^2$. Indeed, we can write

$$\begin{aligned} d_{\mathrm{TV}}(F, Z)&\le \frac{2}{\sigma ^2} {\mathbb {E}}\left( \big \vert \sigma ^2 - \langle DF, - DL^{-1}F \rangle _{{\mathcal {H}}} \big \vert \right) \le \frac{2}{\sigma ^2} \sqrt{ \text {Var}\big ( \langle DF, - DL^{-1}F \rangle _{{\mathcal {H}}} \big ) } \\&\le \frac{4}{\sigma ^2} \sqrt{{\mathcal {A}}} \quad \text {by Proposition}~1.9\text { below.} \end{aligned}$$

#### Proposition 1.9

If $F, G\in {\mathbb {D}}^{2,4}$ have mean zero such that with probability one, $DF, DG\in | {\mathcal {H}}|$ and $D^2F, D^2G\in | {\mathcal {H}}^{\otimes 2}|$, then

1.27 $$\begin{aligned} \mathrm{Var}\Big ( \langle DF, - DL^{-1}G \rangle _{\mathcal {H}} \Big ) = {\mathbb {E}}\big [ ( \text {Cov}(F,G) - \langle DF, - DL^{-1}G \rangle _{\mathcal {H}} )^2 \big ] \le 2 A_1 + 2A_2, \end{aligned}$$

where

$$\begin{aligned} A_1:&= \int _{{\mathbb {R}}_+^6\times {\mathbb {R}}^{6d}} drdr' dsds' d\theta d\theta ' dzdz' dydy' dwdw'\\&\quad \times \gamma _0(\theta - \theta ') \gamma _0(s-s') \gamma _0(r-r') \gamma (z-z') \gamma (w-w') \gamma (y-y')\\&\quad \times \Vert D_{r,z}D_{\theta ,w}F \Vert _4 \Vert D_{s,y}D_{\theta ',w'}F \Vert _4 \Vert D_{r',z'}G\Vert _4 \Vert D_{s', y' }G \Vert _4 \end{aligned}$$

and $A_2$ is defined by switching the positions of *F*, *G* in the definition of $A_1$.

For the sake of completeness, we sketch the proof of Proposition 1.9 in Appendix A.2. Once we have the information on the growth order of $\sigma _R(t)$, we can apply Theorem 1.3 and Proposition 1.9 to obtain the error bounds in Theorem 1.4. The proof of Theorem 1.4 will be given in Sect. 4: In Sect. 4.1, we will establish the limiting covariance structure, which will be used to obtain the quantitative CLTs in Sect. 4.2; Proposition 1.9, combined with a multivariate Malliavin-Stein bound (see *e.g.* [25, Theorem 6.1.2]), also gives us easy access to the convergence of finite-dimensional distributions (*f.d.d. convergence*) for part (1), while in the other parts, the *f.d.d.* convergence follows easily from the dominance of the first chaotic component of $F_R(t)$; finally in Sect. 4.3, we establish the functional CLT by showing the required tightness, which will follow by verifying the well-known criterion of Kolmogorov-Chentsov (see *e.g.* [17, Corollary 16.9]).

### Absolute continuity of the law of the solution to Eq. (1.1)

In this part, we fix the following extra hypothesis on the correlation kernels $\gamma _0,\gamma$.

$$\begin{aligned} \mathbf{(H2)} {\left\{ \begin{array}{ll} \gamma _0 ={\mathcal {F}}\mu _0\text { and }\gamma = {\mathcal {F}} \mu ,\text { where }\mu _0, \mu \text { are nonnegative tempered measures} \\ \text { and have strictly positive densities with respect to the Lebesgue measure. } \end{array}\right. } \end{aligned}$$

The following is the main result of this section.

#### Theorem 1.10

Let $d\in \{1,2\}$ and assume that Hypothesis $\mathbf{(H2)}$ holds. In addition, assume that Hypothesis $\mathbf{(H1)}$ holds if $d=2$. Let *u* be the solution to (1.1). For any $t>0$ and $x \in {\mathbb {R}}^d$, the law of *u*(*t*, *x*) restricted to the set ${\mathbb {R}}\backslash \{0\}$ is absolutely continuous with respect to the Lebesgue measure on ${\mathbb {R}}\backslash \{0\}$.

Let us sketch the proof of Theorem 1.10. In view of the Bouleau-Hirsch criterion for absolute continuity (see [5]), it suffices to prove that for each $m\ge 1$,

1.28 $$\begin{aligned} \Vert D u(t,x)\Vert _{{\mathcal {H}}}>0 \quad \text{ a.s. } \text{ on } \ \Omega _m, \end{aligned}$$

where $\Omega _m =\{ |u(t,x) | \ge 1/m\}$. Notice that

$$\begin{aligned} \Vert D u(t,x)\Vert ^2_{{\mathcal {H}}} = \int _0^t \int _0^t \gamma _0(r-s) \langle D_{r,\bullet }u(t,x) , D_{s,\bullet }u(t,x) \rangle _{0} drds, \end{aligned}$$

where ${\mathcal {P}}_0$ is the completion of $C^\infty _c({\mathbb {R}}^d)$ with respect to the inner product $\langle \cdot , \cdot \rangle _0$ introduced in (2.1). The usual approach to show the positivity of this norm is to get a lower bound for this integral by integrating on a small interval $[ t-\delta , t]^2$ and use that, for *r* close to *t*, $D_{r,y}u(t,x)$ behaves as $G_{t-r}(x-y) u(s,y)$ (see, e.g., [31]). However, for $r\not =s$, the inner product $\langle D_{r,\bullet }u(t,x) , D_{s,\bullet }u(t,x) \rangle _{0}$ is not necessarily non-negative. Our strategy to overcome this difficulty consists in making use of Hypothesis $\mathbf{(H2)}$ in order to show that

$$\begin{aligned} \int _0^t \Vert D_{r,\bullet }u(t,x) \Vert _{0} ^2 dr>0 ~ ~ \text {implies ~ }\Vert D u(t,x)\Vert _{{\mathcal {H}}}>0 \text { (see Lemma} ~A.1). \end{aligned}$$

This allows us to reduce the problem to the non-degeneracy of $\int _{t-\delta } ^t \Vert D_{r,\bullet }u(t,x) \Vert _{0} ^2 dr$ for $\delta$ small enough, which can be handled by the usual arguments. At this point, we will make use of the estimates provided in Theorem 1.3.

For $d=1$, Theorem 1.10 was proved in [2] under stronger assumptions on the covariance structure. The result in Theorem 1.10 for $d=2$ is new. Indeed, the study of the existence (and smoothness) of the density for the stochastic wave equation has been extensively revisited over the last three decades. We refer the readers to [7, 22, 23, 31, 39–41]. In all these articles, the authors considered a stochastic wave equation of the form

$$\begin{aligned} \frac{\partial ^2 u}{\partial t^2}(t,x)=\Delta u(t,x)+b(u(t,x))+\sigma (u(t,x)) \dot{{\mathfrak {X}}}(t,x), \end{aligned}$$

on ${\mathbb {R}}_+\times {\mathbb {R}}^d$, with $d\ge 1$. Here, $\dot{{\mathfrak {X}}}$ denotes a space-time white noise in the case $d=1$, or a Gaussian noise that is white in time and has a spatially homogeneous correlation (slightly more general than that of *W*) in the case $d\ge 2$. The functions $b,\sigma$ are usually assumed to be globally Lipschitz, and such that the following non-degeneracy condition is fulfilled: $|\sigma (z)|\ge C>0$, for all $z\in {\mathbb {R}}$. The temporal nature of the noise $\dot{{\mathfrak {X}}}$ made possible to interpret the solution in the classical Dalang-Walsh sense, making use of all needed martingale techniques. The first attempt to consider a Gaussian noise that is colored in time was in the paper [2], where the hyperbolic Anderson model with spatial dimension one was considered. As mentioned above, in that paper the existence of density was proved under a slightly stronger assumption than Hypothesis $\mathbf{(H2)}$.

The rest of this paper is organized as follows. Section 2 contains preliminary results and the proofs of our main results—Theorems 1.3, 1.4 and 1.10—are given in Sects. 3, 4 and 5 , respectively.

## Preliminary results

This section is devoted to presenting some basic elements of the Malliavin calculus and collecting some preliminary results that will be needed in the sequel.

### Basic Malliavin calculus

Recall that the Hilbert space ${\mathcal {H}}$ is the completion of $C^\infty _c({\mathbb {R}}_+\times {\mathbb {R}}^d)$ under the inner product (1.3) that can be written as

$$\begin{aligned} \big \langle \psi , \phi \big \rangle _{{\mathcal {H}}} = \int _{{\mathbb {R}}_+^2}dsdt \gamma _0(t-s) \big \langle \psi (t, \bullet ), \phi (s, \bullet ) \big \rangle _{0} \quad \text {for }\psi , \phi \in C^\infty _c({\mathbb {R}}_+\times {\mathbb {R}}^d), \end{aligned}$$

where

2.1 $$\begin{aligned} \langle h, g\rangle _0= \int _{{\mathbb {R}}^{2d}}dzdz' \gamma (z-z') h(z) g(z'). \end{aligned}$$

As defined in Sect. 1.2, we denote by ${\mathcal {P}}_0$ the completion of $C^\infty _c({\mathbb {R}}^d)$ with respect to the inner product $\langle h, g\rangle _0$. Let $| {\mathcal {P}}_0|$ be the set of measurable functions $h:{\mathbb {R}}^d \rightarrow {\mathbb {R}}$ such that

2.2 $$\begin{aligned} \int _{{\mathbb {R}}^{2d}}dzdz' \gamma (z-z') |h|(z) |h|(z') <\infty . \end{aligned}$$

Then $|{\mathcal {P}}_0| \subset {\mathcal {P}}_0$ and for $h\in | {\mathcal {P}}_0|$, $\Vert h \Vert ^2_0= \int _{{\mathbb {R}}^{2d}}dzdz' \gamma (z-z') h(z) h(z')$. We define the space $|{\mathcal {H}}|$ in a similar way. For $h,g\in C^\infty _c({\mathbb {R}}^d)$ we can express (2.1) using the Fourier transform:

2.3 $$\begin{aligned} \langle h, g\rangle _0 =\int _{{\mathbb {R}}^{d}} \mu (d\xi ) {\mathcal {F}}h(\xi ) \overline{ {\mathcal {F}}g(\xi )}. \end{aligned}$$

The Parseval-type relation (2.3) also holds for functions $h,g \in L^1({\mathbb {R}}^d) \cap |{\mathcal {P}}_0|$.

For every integer $p\ge 1$, ${\mathcal {H}}^{\otimes p}$ and ${\mathcal {H}}^{\odot p}$ denote the *p*th tensor product of ${\mathcal {H}}$ and its symmetric subspace, respectively. For example, $f_{t,x,n}$ in (1.8) belongs to ${\mathcal {H}}^{\otimes n}$ and ${\widetilde{f}}_{t,x,n}\in {\mathcal {H}}^{\odot n}$; we also have $f\otimes g\in {\mathcal {H}}^{\otimes (n+m)}$, provided $f\in {\mathcal {H}}^{\otimes m}$ and $g\in {\mathcal {H}}^{\otimes n}$; see [25, Appendix B] for more details.

Fix a probability space $(\Omega , {\mathcal {B}}, {\mathbb {P}})$, on which we can construct the isonormal Gaussian process associated to the Gaussian noise ${\dot{W}}$ in (1.1) that we denote by $\{W(\phi ): \phi \in {\mathcal {H}}\}$. That is, $\{W(\phi ): \phi \in {\mathcal {H}}\}$ is a *centered Gaussian family* of real-valued random variables defined on $(\Omega , {\mathcal {B}}, {\mathbb {P}})$ such that ${\mathbb {E}}[ W(\psi ) W(\phi ) ] = \langle \psi , \phi \rangle _{{\mathcal {H}}}$ for any $\psi , \phi \in {\mathcal {H}}$. We will take ${\mathcal {B}}$ to be the $\sigma$-algebra $\sigma \{W\}$ generated by the family of random variables $\{ W(h): h\in C^\infty _c({\mathbb {R}}_+\times {\mathbb {R}}^d)\}$.

In the sequel, we recall some basics on Malliavin calculus from the books [25, 27].

Let $C^\infty _\text {poly}({\mathbb {R}}^n)$ denote the space of smooth functions with all their partial derivatives having at most polynomial growth at infinity and let ${\mathcal {S}}$ denote the set of simple smooth functionals of the form

$$\begin{aligned} F = f\big (W(h_1), \dots , W(h_n) \big )\text { for }f\in C^\infty _\text {poly}({\mathbb {R}}^n)\text { and }h_i\in {\mathcal {H}}, 1\le i\le n. \end{aligned}$$

For such a random variable *F*, its Malliavin derivative *DF* is the ${\mathcal {H}}$-valued random variable given by

$$\begin{aligned} DF = \sum _{i=1}^n \frac{\partial f}{\partial x_i} \big (W(h_1), \dots , W(h_n) \big ) h_i. \end{aligned}$$

And similarly its *m*th Malliavin derivative $D^mF$ is the ${\mathcal {H}}^{\otimes m}$-valued random variable given by

2.4 $$\begin{aligned} D^mF = \sum _{i_1, \dots , i_m=1}^n \frac{\partial ^m f }{\partial x_{i_1} \cdots \partial x_{i_m}} \big (W(h_1), \dots , W(h_n) \big ) h_{i_1}\otimes \cdots \otimes h_{i_m}, \end{aligned}$$

which is an element in $L^p(\Omega ; {\mathcal {H}}^{\odot m})$ for any $p\in [1,\infty )$. It is known that the space ${\mathcal {S}}$ is dense in $L^p(\Omega , \sigma \{W\}, {\mathbb {P}})$ and

$$\begin{aligned} D^m: {\mathcal {S}} \longrightarrow L^p(\Omega ; {\mathcal {H}}^{\odot m}) \end{aligned}$$

is closable for any $p\in [1,\infty )$; see *e.g.* Lemma 2.3.1 and Proposition 2.3.4 in [25]. Let ${\mathbb {D}}^{m,p}$ be the closure of ${\mathcal {S}}$ under the norm

$$\begin{aligned} \big \Vert F \big \Vert _{{\mathbb {D}}^{m,p}}= & {} \Big ( {\mathbb {E}}\big [ | F |^p \big ] + {\mathbb {E}}\big [ \Vert D F \Vert ^p_{{\mathcal {H}}} \big ] + \cdots + {\mathbb {E}}\big [ \Vert D^mF \Vert ^p_{{\mathcal {H}}^{\otimes m}} \big ] \Big )^{1/p}~\text {and}\\ \text {let } {\mathbb {D}}^{\infty }:= & {} \bigcap _{m,p\ge 1}{\mathbb {D}}^{m,p}. \end{aligned}$$

Now, let us introduce the adjoint of the derivative operator $D^m$. Let $\text {Dom}(\delta ^m)$ be the set of random variables $v\in L^2 ( \Omega ; {\mathcal {H}}^{\otimes m} )$ such that there is a constant $C_v>0$ for which

$$\begin{aligned} \Big \vert {\mathbb {E}}\big [ \langle D^m F, v \rangle _{{\mathcal {H}}^{\otimes m}} \big ] \Big \vert \le C_v \Vert F \Vert _2 \quad \text {for all }F\in {\mathcal {S}}. \end{aligned}$$

By *Riesz representation theorem*, there is a unique random variable, denoted by $\delta ^m(v)$, such that the following duality relationship holds:

2.5 $$\begin{aligned} {\mathbb {E}}\big [ F \delta ^m(v) \big ] = {\mathbb {E}}\big [ \langle D^m F, v \rangle _{{\mathcal {H}}^{\otimes m}} \big ]. \end{aligned}$$

Equality (2.5) holds for all $v\in \text {Dom}(\delta ^m)$ and all $F\in {\mathbb {D}}^{m,2}$. In the simplest case when $F = f( W(h))$ with $h\in {\mathcal {H}}$ and $f\in C^1_\text {poly}({\mathbb {R}})$, we have $\delta (h) = W(h)\sim N(0, \Vert h\Vert _{\mathcal {H}}^2)$ and equality (2.5) reduces to

$$\begin{aligned} {\mathbb {E}}\big [ f(W(h)) W(h) \big ] = {\mathbb {E}}\big [ f'(W(h) ) \big ] \Vert h \Vert _{{\mathcal {H}}}^2, \end{aligned}$$

which is exactly part of the Stein’s lemma recalled below: For $\sigma \in (0,\infty )$ and an integrable random variable *Z*, Stein’s lemma (see *e.g.* [25, Lemma 3.1.2]) asserts that

2.6 $$\begin{aligned} Z\sim N(0, \sigma ^2) ~\text {if and only if} ~ {\mathbb {E}}[ Z f(Z) ] = \sigma ^2 {\mathbb {E}}[ f'(Z) ], \end{aligned}$$

for any differentiable function $f:{\mathbb {R}}\rightarrow {\mathbb {R}}$ such that the above expectations are finite. The operator $\delta$ is often called the *Skorokhod integral* since in the case of the Brownian motion, it coincides with an extension of the Itô integral introduced by Skorokhod, see *e.g.* [29]. Then we can say $\text {Dom}(\delta ^m)$ is the space of Skorokhod integrable random variables with values in ${\mathcal {H}}^{\otimes m}$.

The Wiener-Itô chaos decomposition theorem asserts that $L^2(\Omega , \sigma \{W\}, {\mathbb {P}})$ can be written as a direct sum of mutually orthogonal subspaces:

$$\begin{aligned} L^2(\Omega , \sigma \{W\}, {\mathbb {P}}) = \bigoplus _{n\ge 0} {\mathbb {C}}_n^W, \end{aligned}$$

where ${\mathbb {C}}_0^W$, identified as ${\mathbb {R}}$, is the space of constant random variables and ${\mathbb {C}}_n^W = \{ \delta ^n( h): h \in {\mathcal {H}}^{\otimes n} ~\text {is deterministic}\}$, for $n\ge 1$, is called the *n*th *Wiener chaos* associated to *W*. Note that the first Wiener chaos consists of centered Gaussian random variables. When $h \in {\mathcal {H}}^{\otimes n}$ is deterministic, we write $I_n(h) = \delta ^n( h )$ and we call it the *n*th multiple integral of *h* with respect to *W*. By the symmetry in (2.4) and the duality relation (2.5), $\delta ^n( h ) = \delta ^n( {\widetilde{h}} )$ with ${\widetilde{h}}$ the canonical symmetrization of *h*, so that we have $I_n(h) = I_n({\widetilde{h}})$ for any $h \in {\mathcal {H}}^{\otimes n}$. The above decomposition can be rephrased as follows. For any $F\in L^2(\Omega , \sigma \{W\}, {\mathbb {P}})$,

2.7 $$\begin{aligned} F = {\mathbb {E}}[F] + \sum _{n\ge 1} I_n(f_n), \end{aligned}$$

with $f_n \in {\mathcal {H}}^{\odot n}$ uniquely determined for each $n\ge 1$. Moreover, the (modified) isometry property holds

2.8 $$\begin{aligned} {\mathbb {E}}\big [ I_p(f) I_q(g) \big ] = p! {\mathbf {1}}_{\{ p=q\}} \big \langle {\widetilde{f}}, {\widetilde{g}} \big \rangle _{{\mathcal {H}}^{\otimes p}}, \end{aligned}$$

for any $f\in {\mathcal {H}}^{\otimes p}$ and $g\in {\mathcal {H}}^{\otimes q}$. We have the following *product formula*: For $f\in {\mathcal {H}}^{\odot p}$ and $g\in {\mathcal {H}}^{\odot q}$,

2.9 $$\begin{aligned} I_p(f) I_q(g) = \sum _{r=0}^{p\wedge q} r! \left( {\begin{array}{c}p\\ r\end{array}}\right) \left( {\begin{array}{c}q\\ r\end{array}}\right) I_{p+q-2r}( f\otimes _r g ), \end{aligned}$$

where $f\otimes _r g$ is the *r*-contraction between *f* and *g*, which is an element in ${\mathcal {H}}^{\otimes (p+q-2r)}$ defined as follows. Fix an orthonormal basis $\{e_i, i\in {\mathcal {O}}\}$ of ${\mathcal {H}}$. Then, for $1\le r \le p\wedge q$,

2.10 $$\begin{aligned} f\otimes _r g&:= \sum _{i_1,\dots ,i_p, j_1,\dots ,j_q\in {\mathcal {O}}} \langle f, e_{i_1} \otimes \cdots \otimes e_{i_p} \rangle _{{\mathcal {H}}^{\otimes p}}\langle g, e_{j_1} \otimes \cdots \otimes e_{j_q}\rangle _{{\mathcal {H}}^{\otimes p}}\nonumber \\&\quad \times {\mathbf {1}}_{\{ i_k=j_k, \forall k=1,\dots ,r \}} e_{i_{r+1}} \otimes \cdots \otimes e_{i_p} \otimes e_{j_{r+1}} \otimes \cdots \otimes e_{j_q}. \end{aligned}$$

In the particular case when *f*, *g* are real-valued functions, we can write

$$\begin{aligned}&(f\otimes _r g)( \pmb {t_{p-r}}, \pmb {x_{p-r}} , \pmb {t'_{q-r}} , \pmb {x'_{q-r}} )\\&\quad = \int _{{\mathbb {R}}_+^{2r} \times {\mathbb {R}}^{2rd}} d\pmb {s_r}d\pmb {s'_r} d\pmb {y_r}d\pmb {y'_r} \left( \prod _{j=1}^r \gamma _0(s_j-s'_j) \gamma (y_j-y'_j) \right) \\&\quad \times f( \pmb {s_r},\pmb {t_{p-r}}, \pmb {y_r}, \pmb {x_{p-r}} ) g( \pmb {s'_r},\pmb {t'_{q-r}}, \pmb {y'_r}, \pmb {x'_{q-r}} ), \end{aligned}$$

provided the above integral exists. For $F\in {\mathbb {D}}^{m,2}$ with the representation (2.7) and $m\ge 1$, we have

2.11 $$\begin{aligned} D^m_{\bullet } F = \sum _{n\ge m} \frac{n!}{(n-m)!} I_{n-m}\big ( f_n(\bullet ,*)\big ) ~\text {with convergence in }L^2(\Omega ; {\mathcal {H}}^{\otimes m}), \end{aligned}$$

where $I_{n-m}\big ( f_n(\bullet ,*)\big )$ is understood as the $(n-m)$th multiple integral of $f_n(\bullet ,*)\in {\mathcal {H}}^{\otimes (n-m)}$ for fixed $\bullet$. We can write

$$\begin{aligned} D^m_{\pmb {s_m}, \pmb {y_m}} F = \sum _{n\ge m} \frac{n!}{(n-m)!} I_{n-m}\big ( f_n(\pmb {s_m}, \pmb {y_m};*)\big ), \end{aligned}$$

whenever the above series makes sense and converges in $L^2(\Omega )$. With the decomposition (2.11) in mind, we have the following Gaussian Poincaré inequality: For $F\in {\mathbb {D}}^{1,2}$, it holds that

2.12 $$\begin{aligned} \text {Var}(F) \le {\mathbb {E}}\big [ \Vert DF \Vert _{\mathcal {H}}^2 \big ]. \end{aligned}$$

In fact, if *F* has the representation (2.7), then

$$\begin{aligned} \text {Var}(F) = \sum _{n\ge 1} n! \Vert f_n \Vert _{{\mathcal {H}}^{\otimes n}}^2 \quad \text{ and } \quad {\mathbb {E}}\big [ \Vert DF \Vert _{\mathcal {H}}^2 \big ] = \sum _{n\ge 1} n n! \Vert f_n \Vert _{{\mathcal {H}}^{\otimes n}}^2, \end{aligned}$$

which gives us (2.12) and, moreover, indicates that the equality in (2.12) holds only when $F\in {\mathbb {C}}^W_0 \oplus {\mathbb {C}}^W_1$, that is, only when *F* is a real Gaussian random variable.

Now let us mention the particular case when the Gaussian noise is white in time, which is used in the reduction step in Sect. 3.2. First, let us denote

$$\begin{aligned} {\mathcal {H}}_0:=L^2\big ({\mathbb {R}}_{+};{\mathcal {P}}_0\big ) \end{aligned}$$

and point out that the following inequality reduces many calculations to the case of the white noise in time. For any nonnegative function $f\in {\mathcal {H}}_0^{\otimes n}$ that vanishes outside $([0,t] \times {\mathbb {R}}^d)^n$,

2.13 $$\begin{aligned} \Vert f\Vert _{{\mathcal {H}}^{\otimes n}}^2 \le \Gamma _t^n \Vert f\Vert _{{\mathcal {H}}_0^{\otimes n}}^2, \end{aligned}$$

where^7^

$$\begin{aligned} \Gamma _t=2\int _{0}^t \gamma _0(s)ds \quad \mathrm{and} \quad \Vert f\Vert _{{\mathcal {H}}_0^{\otimes n}}^2=\int _{[0,t]^n}\Vert f(t_1,\cdot ,\ldots ,t_n,\cdot )\Vert _{{\mathcal {P}}_0^{\otimes n}}^2 dt_1 \cdots dt_n; \end{aligned}$$

whenever no ambiguity arises, we write $\Vert f\Vert _0:=\Vert f\Vert _{{\mathcal {P}}_0^{\otimes n}}$ so that $\Vert f\Vert _{{\mathcal {H}}_0^{\otimes n}}^2=\int _{[0,t]^n}\Vert f( \pmb {t_n} ,\bullet )\Vert _{0}^2 d\pmb {t_n}.$

Let $\dot{{\mathfrak {X}}}$ denote the Gaussian noise that is white in time and has the same spatial correlation as *W*. More precisely, $\{{\mathfrak {X}}(f): f\in {\mathcal {H}}_0\}$ is a centered Gaussian family with covariance

$${\mathbb {E}}[ {\mathfrak {X}}(f) {\mathfrak {X}}(g) ] =\langle f, g \rangle _{{\mathcal {H}}_0}, \quad \text{ for } \text{ any } f,g\in {\mathcal {H}}_0.$$

Denote by $I^{{\mathfrak {X}}}_p$ the *p*-th multiple stochastic integral with respect to ${\mathfrak {X}}$. The product formula (2.9) still holds with *W* replaced by the noise ${\mathfrak {X}}$. Moreover, if $f\in {\mathcal {H}}^{\otimes p}$ and $g\in {\mathcal {H}}^{\otimes q}$ have disjoint temporal supports,^8^ then we have $f\otimes _r g =0$ for $r=1,\dots , p\wedge q$ and the product formula (2.9) reduces to

2.14 $$\begin{aligned} I^{{\mathfrak {X}}}_p(f) I^{{\mathfrak {X}}}_q(g) = I^{{\mathfrak {X}}}_{p+q}(f\otimes g). \end{aligned}$$

In this case, the random variables $I^{{\mathfrak {X}}}_p(f)$ and $I^{{\mathfrak {X}}}_q(g)$ are independent by the Üstünel-Zakai-Kallenberg criterion (see Exercise 5.4.8 of [25]) and note that we do not need to assume *f*, *g* to be symmetric in (2.14).

Now let us introduce the Ornstein-Uhlenbeck operator *L* that can be defined as follows. We say that *F* belongs to the $\text {Dom}(L)$ if $F\in {\mathbb {D}}^{1,2}$ and $DF\in \text {Dom}(\delta )$; in this case, we let $LF = -\delta DF$. For $F\in L^2(\Omega )$ of the form (2.7), $F\in \text {Dom}(L)$ if and only if $\sum _{n\ge 1} n^2 n! \Vert f_n \Vert _{{\mathcal {H}}^{\otimes n}}^2 <\infty .$ In this case, we have $LF = \sum _{n\ge 1} -n I_n(f_n)$. Using the chaos expansion, we can also define the Ornstein-Uhlenbeck semigroup $\{P_t = e^{tL}, t\in {\mathbb {R}}_+\}$ and the pseudo-inverse $L^{-1}$ of the Ornstein-Uhlenbeck operator *L* as follows. For $F\in L^2(\Omega )$ having the chaos expansion (2.7),

$$\begin{aligned} P_t F := \sum _{n\ge 0} e^{-nt} I_n(f_n) \quad \mathrm{and}\quad L^{-1} F = \sum _{n\ge 1} -\frac{1}{n} I_n(f_n). \end{aligned}$$

Observe that for any centered random variable $F\in L^2(\Omega , \sigma \{W\}, {\mathbb {P}})$, $LL^{-1}F = F$ and for any $G\in \text {Dom}(L)$, $L^{-1} LG = G - {\mathbb {E}}[G].$ The above expression and the modified isometry property (2.8) give us the contraction property of $P_t$ on $L^2(\Omega )$, that is, for $F\in L^2(\Omega , \sigma \{W\}, {\mathbb {P}})$, $\Vert P_t F\Vert _2 \le \Vert F\Vert _2$. Moreover, $P_t$ is a contraction operator on $L^q(\Omega )$ for any $q\in [1,\infty )$; see [25, Proposition 2.8.6].

Finally, let us recall Nelson’s *hypercontractivity property* of the Ornstein-Uhlenbeck semigroup: For $F\in L^q(\Omega , \sigma \{W\}, {\mathbb {P}})$ with $q\in (1,\infty )$, it holds for each $t\ge 0$ that $\Vert P_t F \Vert _{q_t} \le \Vert F \Vert _q$ with $q_t = 1 + (q-1)e^{2t}$. In this paper, we need one of its consequences – a moment inequality comparing $L^q(\Omega )$-norms on a fixed chaos:

2.15 $$\begin{aligned} \text {If }F\in {\mathbb {C}}^W_n\text { and }p\in [2,\infty ),\text { then } \Vert F\Vert _p \le (p-1)^{n/2}\Vert F\Vert _2; \end{aligned}$$

see *e.g.* [25, Corollary 2.8.14].

### Inequalities

Let us first present a few inequalities, which will be used in Sect. 3.

#### Lemma 2.1

Fix an integer $d\ge 1$. Suppose that either one of the following conditions hold:

$$\begin{aligned} \mathrm{(a)} \gamma \in L^{\ell }({\mathbb {R}}^d){ for some }\ell \in (1,\infty ) \quad \mathrm{(b)} \gamma (x)=|x|^{-\beta }\,{ for some }\beta \in (0,d). \end{aligned}$$

Define

$$\begin{aligned} q= {\left\{ \begin{array}{ll} \ell /(2\ell -1) &{} \text {in case (a)} \\ d/(2d-\beta )&{} \text {in case (b).} \end{array}\right. } \end{aligned}$$

Then, for any $f,g \in L^{2q}({\mathbb {R}}^{d})$,

$$\int _{{\mathbb {R}}^d} \int _{{\mathbb {R}}^d}f(x)g(y)\gamma (x-y)dxdy \le C_\gamma \Vert f\Vert _{L^{2q}({\mathbb {R}}^d)} \Vert g\Vert _{L^{2q}({\mathbb {R}}^d)},$$

where $C_\gamma =\Vert \gamma \Vert _{L^{\ell }({\mathbb {R}}^d)}$ in case (a), and $C_\gamma =C_{d,\beta }$ is the constant (depending on $d,\beta )$ that appears in the Hardy–Littlewood–Sobolev inequality (2.16) below, in case (b).

#### Proof

In the case $d=2$, this result was essentially proved on page 15 of [35] in case (a), and on page 6 of [4] in case (b). We reproduce the arguments here for the sake of completeness.

In case (a), we apply Hölder’s inequality and *Young’s convolution inequality*:

$$\begin{aligned}&\int _{{\mathbb {R}}^d}f(x)(g*\gamma )(x)dx \le \Vert f\Vert _{L^{\frac{2\ell }{2\ell -1}}({\mathbb {R}}^d)} \Vert g*\gamma \Vert _{L^{2\ell }({\mathbb {R}}^d)} \\&\quad \le \Vert f\Vert _{L^{\frac{2\ell }{2\ell -1}}({\mathbb {R}}^d)} \Vert g\Vert _{L^{\frac{2\ell }{2\ell -1}}({\mathbb {R}}^d)} \Vert \gamma \Vert _{L^{\ell }({\mathbb {R}}^d)}. \end{aligned}$$

In case (b), we apply Hölder’s inequality and *Hardy-Littlewood-Sobolev inequality*:

2.16 $$\begin{aligned}&\int _{{\mathbb {R}}^d}f(x)(g*\gamma )(x)dx \le \Vert f\Vert _{L^{\frac{2d}{2d-\beta }}({\mathbb {R}}^d)} \Vert g*\gamma \Vert _{L^{2d/\beta }({\mathbb {R}}^d)} \nonumber \\&\quad \le C_{d,\beta }\Vert f\Vert _{L^{\frac{2d}{2d-\beta }}({\mathbb {R}}^d)} \Vert g\Vert _{L^{\frac{2d}{2d-\beta }}({\mathbb {R}}^d)}. \end{aligned}$$

This concludes the proof. $\square$

To deal with case (c) in $\mathbf{(H1)}$, we need the following modification of Lemma 2.1.

#### Lemma 2.2

Suppose that $\gamma (x_1,\ldots ,x_d)=\prod _{i=1}^{d}\gamma _i(x_i)$, where for each $i\in \{1,\ldots ,d\}$,

$$\begin{aligned} (M1)\gamma _i\in \! L^{\ell _i}(\mathbb {R}) {for}\, {some}\, \ell _i\!\in \!(1, \infty ) \quad \mathrm{or}\quad (M2)\gamma _i(x) \!=\!|x| ^{-\beta _i} {for}\, {some}\, \beta _i \!\in \! (0,1) \end{aligned}$$

Let $q_i=\ell _i/(2\ell _i-1)$ in case (M1) and $q_{i}=1/(2-\beta _i)$ in case (M2). Let $q=\max \{q_i: i=1,\dots ,d\}$.

If $f, g \in L^{2q}({\mathbb {R}}^d)$ satisfy $f(x)=g(x)=0$ for $x \not \in \prod _{i=1}^d[a_i,b_i]$ for some real numbers $a_i<b_i$,^9^ then

2.17 $$\begin{aligned} \int _{{\mathbb {R}}^d} \int _{{\mathbb {R}}^d}f(x)g(y)\gamma (x-y)dxdy \le \Lambda ^{\nu } C_\gamma \Vert f\Vert _{L^{2q}({\mathbb {R}}^d)} \Vert g\Vert _{L^{2q}({\mathbb {R}}^d)}, \end{aligned}$$

with $\Lambda =\max \{b_i-a_i;i=1,\ldots ,d\}$, $C_\gamma = \prod _{i=1}^{d}C_{\gamma _i}$ and $\nu = \sum _{i=1}^{d} (q_i^{-1} - q^{-1})$. In particular, when $q_i=q$ for all $i\in \{1,\ldots ,d\}$, we have

$$\begin{aligned} \int _{{\mathbb {R}}^d} \int _{{\mathbb {R}}^d}f(x)g(y)\gamma (x-y)dxdy \le C_\gamma \Vert f\Vert _{L^{2q}({\mathbb {R}}^d)} \Vert g\Vert _{L^{2q}({\mathbb {R}}^d)}. \end{aligned}$$

The constants $C_{\gamma _i}$ are defined as in Lemma  2.1.

#### Proof

By Lemma 2.1, inequality (2.17) holds for $d=1$ with $\nu =0$. Now let us consider $d\ge 2$ and prove inequality (2.17) by induction. Suppose (2.17) holds for $d\le k-1$ $(k\ge 2)$. We use the notation $x=(x_1,\ldots ,x_k)=:\pmb {x_k}.$

Without loss of any generality we assume $q_1\ge q_2\ge \cdots \ge q_k$, so that $q=q_1$. Applying the initial step $(d=1)$ yields

2.18 $$\begin{aligned}&\int _{{\mathbb {R}}^{2k}} d\pmb {x_k} d\pmb {y_k} f(\pmb {x_k} ) g(\pmb {y_k}) \prod _{i=1}^k \gamma _i(x_i-y_i) \nonumber \\&\quad \le C_{\gamma _k} \!\int _{{\mathbb {R}}^{2(k-1)}} d\pmb {x_{k-1}} d\pmb {y_{k-1}} \big \Vert f(\pmb {x_{k-1}}, \bullet ) \big \Vert _{L^{2q_k}({\mathbb {R}})} \big \Vert g(\pmb {y_{k-1}}, \bullet ) \big \Vert _{L^{2q_k}({\mathbb {R}})} \!\prod _{i=1}^{k-1} \gamma _i(x_i\!-\!y_i). \end{aligned}$$

By the induction hypothesis, we can bound the right-hand side of (2.18) by

$$\begin{aligned}&\left( \prod _{i=1}^k C_{\gamma _i} \right) \Lambda ^{\nu ^*} \left( \int _{{\mathbb {R}}^{k-1}} \big \Vert f(\pmb {x_{k-1}}, \bullet ) \big \Vert _{L^{2q_k}({\mathbb {R}})}^{2q} d\pmb {x_{k-1}} \right) ^{\frac{1}{2q}}\\&\quad \times \,\left( \int _{{\mathbb {R}}^{k-1}} \big \Vert g(\pmb {y_{k-1}}, \bullet ) \big \Vert _{L^{2q_k}({\mathbb {R}})}^{2q} d\pmb {y_{k-1}} \right) ^{\frac{1}{2q}}, \end{aligned}$$

with $\nu ^*= \sum _{i=1}^{k-1}( q_i^{-1} - q^{-1})$. By Hölder’s inequality,

$$\begin{aligned}&\left( \int _{{\mathbb {R}}^{k-1}} \big \Vert f(\pmb {x_{k-1}}, \bullet ) \big \Vert _{L^{2q_k}({\mathbb {R}})}^{2q} d\pmb {x_{k-1}} \right) ^{\frac{1}{2q}}\\&\quad = \left( \int _{{\mathbb {R}}^{k-1}} \left[ \int _{a_k}^{b_k} \big \vert f(\pmb {x_{k-1}}, x_k ) \big \vert ^{2q_k} dx_k \right] ^{\frac{2q}{2q_k}} d\pmb {x_{k-1}} \right) ^{\frac{1}{2q}} \\&\quad \le \Lambda ^{\frac{1}{2q_k} - \frac{1}{2q}} \left( \int _{{\mathbb {R}}^{k-1}} \int _{a_k}^{b_k} \big \vert f(\pmb {x_{k-1}}, x_k ) \big \vert ^{2q} dx_k d\pmb {x_{k-1}} \right) ^{\frac{1}{2q}}. \end{aligned}$$

A similar inequality holds for *g*. Since $\nu ^*+ ( q_k^{-1} - q^{-1}) = \sum _{i=1}^{k} ( q_i^{-1} - q^{-1})$, inequality (2.17) holds for $d=k$. $\square$

We will need the following generalization of Lemmas 2.1 and 2.2.

#### Lemma 2.3

1. Under the conditions of Lemma 2.1, for any $f,g \in L^{2q}({\mathbb {R}}^{md})$
  2.19 $$\begin{aligned} \int _{{\mathbb {R}}^{2md}} f(\pmb {x_m}) g(\pmb {y_m}) \prod _{j=1}^m \gamma (x_j-y_j) d\pmb {x_m} d\pmb {y_m} \le C_\gamma ^m \Vert f \Vert _{L^{2q}({\mathbb {R}}^{md})} \Vert g \Vert _{L^{2q}({\mathbb {R}}^{md})}, \end{aligned}$$
  where $C_\gamma$ is the same constant as in Lemma 2.1. Here $\pmb {x_m} = (x_1, \dots , x_m)$ with $x_i\in {\mathbb {R}}^d$.
2. Let $\gamma , C_{\gamma }$ and *q* be given as in Lemma 2.2. If $f,g \in L^{2q}({\mathbb {R}}^{md})$ satisfy $f(\pmb {x_{md}}) = g(\pmb {x_{md}})=0$ for $\pmb {x_{md}} \notin \prod _{i=1}^{md} [a_i, b_i]$ for some real numbers $a_i<b_i$, then inequality (2.19) holds with $C_\gamma$ replaced by $\Lambda ^\nu C_\gamma$, where $\Lambda =\max \{ b_i -a_i : i=1,\dots , md\}$ and $\nu = \sum _{i=1}^d ( q_i^{-1} - q^{-1})$. Here $\pmb {x_{md}} = (x_1, \dots , x_{md})$ with $x_i\in {\mathbb {R}}$.

#### Proof

The proof will be done by induction on *m* simultaneously for both cases (1) and (2). Let $C=C_{\gamma }$ in case (1) and $C=\Lambda ^{\nu }C_{\gamma }$ in case (2). The results are true for $m=1$ by Lemmas 2.1 and 2.2. Assume that the results hold for $m-1$. Applying the inequality for $m=1$ yields

$$\begin{aligned}&\int _{{\mathbb {R}}^{2dm}} f(\pmb {x_m}) g(\pmb {y_m}) \prod _{j=1}^m \gamma (x_j-y_j) d\pmb {x_m} d\pmb {y_m}\\&\quad \le C \int _{{\mathbb {R}}^{2d(m-1)}} \Vert f(\pmb {x_{m-1}},\bullet ) \Vert _{L^{2q} ({\mathbb {R}}^d)} \Vert g(\pmb {y_{m-1}},\bullet ) \Vert _{L^{2q} ({\mathbb {R}}^d)}\\&\qquad \times \prod _{j=1}^{m-1} \gamma (x_j-y_j) d\pmb { x_{m-1}} d\pmb {y_{m-1}}. \end{aligned}$$

By the induction hypothesis, the latter term can be bounded by

$$\begin{aligned}&C^m \left( \int _{{\mathbb {R}}^{d(m-1)}} \Vert f(\pmb {x_{m-1}},\bullet )\Vert ^{2q}_{L^{2q} ({\mathbb {R}}^d)} d\pmb {x_{m-1}} \right) ^{\frac{1}{2q}}\\&\qquad \times \left( \int _{{\mathbb {R}}^{d(m-1)}} \Vert g(\pmb {x_{m-1}},\bullet ) \Vert ^{2q} _{L^{2q} ({\mathbb {R}}^d)} d\pmb {x_{m-1}}\right) ^{\frac{1}{2q}}, \end{aligned}$$

which completes the proof. $\square$

Let us return to the three cases of Hypothesis $\mathbf{(H1)}$. Lemma 2.1 indicates that $L^{2q}({\mathbb {R}}^{2})$ is continuously embedded into ${\mathcal {P}}_{0}$, with $q\in (1/2, 1)$ given by

2.20 $$\begin{aligned} q= {\left\{ \begin{array}{ll} \ell /(2\ell -1) &{} \text{ in } \text{ case } (\texttt {a}), \\ 2/(4-\beta )&{} \text{ in } \text{ case } (\texttt {b}). \end{array}\right. } \end{aligned}$$

Recall that ${\mathcal {P}}_{0}$ has been defined at the beginning of Sect. 2.1. Moreover, for any $f, g\in L^{2q}({\mathbb {R}}^2)$,

2.21 $$\begin{aligned} \int _{{\mathbb {R}}^4} \big \vert f(x)g(x) \big \vert \gamma (x-y)dxdy \le D_{\gamma } \Vert f\Vert _{L^{2q}({\mathbb {R}}^2)}\Vert g\Vert _{L^{2q}({\mathbb {R}}^2)}, \end{aligned}$$

where

2.22 $$\begin{aligned} D_{\gamma }= {\left\{ \begin{array}{ll} \Vert \gamma \Vert _{L^{\ell }({\mathbb {R}}^2)} &{} \text{ in } \text{ case } (\texttt {a}), \\ C_{2,\beta }&{} \text{ in } \text{ case } (\texttt {b}). \end{array}\right. } \end{aligned}$$

For case (c) of Hypothesis $\mathbf{(H1)}$, we consider three sub-cases:

$$\begin{aligned} {\left\{ \begin{array}{ll} &{}\mathrm{(i)} ~ \gamma _i\in L^{\ell _i}({\mathbb {R}}) ~\text {for some }\ell _i>1, i=1,2; \\ &{}\mathrm{(ii)} ~\gamma _i(x_i) =|x_i|^{-\beta _i}~\text {for some }\beta _i\in (0,1), i=1,2; \\ &{}\mathrm{(iii)} ~\gamma _1\in L^{\ell }({\mathbb {R}}) ~\text {for some }\ell \in (1,\infty )\text { and }\gamma _2(x_2)=|x_2|^{-\beta }\text { for some }\beta \in (0,1). \end{array}\right. } \end{aligned}$$

Lemma 2.2 implies that, for any $f, g\in L^{2q}({\mathbb {R}}^2)$ with

2.23 $$\begin{aligned} q= {\left\{ \begin{array}{ll} \max \{\ell _i/(2\ell _i-1) : i=1,2\}&{} \text{ in } \text{ case } \text{(i) } \\ \max \{1/(2-\beta _i): i=1,2\}&{} \text{ in } \text{ case } \text{(ii) }\\ \max \{ \ell /(2\ell -1), 1/(2-\beta )\}&{} \text{ in } \text{ case } \text{(iii) } \end{array}\right. }, \end{aligned}$$

such that *f*, *g* vanish outside a box with side lengths bounded by $\Lambda$, then inequality (2.21) still holds with

2.24 $$\begin{aligned} D_\gamma ={\left\{ \begin{array}{ll} \Vert \gamma _1\Vert _{L^{\ell _1}({\mathbb {R}})} \Vert \gamma _2\Vert _{L^{\ell _2}({\mathbb {R}})} \Lambda ^{ |\frac{1}{\ell _1} - \frac{1}{\ell _2}|}&{} \text{ in } \text{ case } \text{(i) } \\ C_{1,\beta _1}C_{1,\beta _2} \Lambda ^{ | \beta _1-\beta _2|} &{} \text{ in } \text{ case } \text{(ii) }\\ C_{1,\beta } \Vert \gamma _1\Vert _{L^\ell ({\mathbb {R}})} \Lambda ^{ | \frac{1}{\ell }-\beta | } &{} \text{ in } \text{ case } \text{(iii) } \end{array}\right. }, \end{aligned}$$

where the constants $C_{1,\beta _i}$ are given as in Lemma 2.1.

From Lemma 2.3, we deduce that in cases (a) and (b),

2.25 $$\begin{aligned} \Vert f\Vert _{{\mathcal {H}}_0^{\otimes n}}^2\le D_{\gamma }^n \int _{[0,t]^n} \Vert f( \pmb {t_n},\bullet )\Vert _{L^{2q}({\mathbb {R}}^{2n})}^2 d\pmb {t_n}, \end{aligned}$$

for any measurable function $f:( {\mathbb {R}}_+ \times {\mathbb {R}}^2)^n \rightarrow {\mathbb {R}}$ such that *f* vanishes outside $([0,t] \times {\mathbb {R}}^2)^n$; in case (c), inequality (2.25) holds true for any measurable function $f: ({\mathbb {R}}_+ \times {\mathbb {R}}^{2})^n \rightarrow {\mathbb {R}}$ such that

$$\begin{aligned} f(t_1,x_1, \dots , t_n, x_n) = f(\pmb {t_n}, \pmb {x_n}) = 0~\text {for } \pmb {t_n}\notin [0,t]^n\text { and }\pmb {x_n}\notin \prod _{i=1}^{2n} [a_i, b_i] \end{aligned}$$

with $$\Lambda :=\max \{ b_i-a_i : i=1,\dots , 2n\}<\infty $$.

Let us present a few facts on the fundamental solution *G*. When $d=2$,

2.26 $$\begin{aligned} \Vert G_t\Vert _{L^p({\mathbb {R}}^2)}= & {} \left( \frac{(2\pi )^{1-p}}{2-p} \right) ^{1/p}t^{\frac{2}{p}-1} \quad \text{ for } \text{ all }~ p \in (0,2), \end{aligned}$$

2.27 $$\begin{aligned} G_t^{p}(x)\le & {} (2\pi t)^{q-p} G_t^{q}(x) \quad \text{ for } \text{ all } ~ p<q, \end{aligned}$$

and

2.28 $$\begin{aligned} {\mathbf {1}}_{\{|x|<t\}} \le 2\pi t G_t(x). \end{aligned}$$

We will use also the following estimate.

#### Lemma 2.4

(Lemma 4.3 of [4]) For any $q \in (1/2,1)$ and $d=2$,

$$\begin{aligned} \int _r^t (G_{t-s}^{2q} * G_{s-r}^{2q})^{1/q}(z)ds \le A_{q} (t-r)^{\frac{1}{q}-1} G_{t-r}^{2-\frac{1}{q}}(z), \end{aligned}$$

where $A_{q}>0$ is a constant depending on *q*.

Finally, we record the expression of the Fourier transform of $G_t$ for $d\in \{1,2\}$:

2.29 $$\begin{aligned} {\mathcal {F}} G_t(\xi ) = \int _{{\mathbb {R}}^d} e^{-i \xi \cdot x} G_t(x) dx = \frac{\sin ( t | \xi | )}{|\xi |}=: {\widehat{G}}_t(\xi ). \end{aligned}$$

Note that (see e.g. (3.4) of [3])

2.30 $$\begin{aligned} \big \vert {\widehat{G}} _t(\xi )\big \vert ^2 \le 2(t^2\vee 1) \frac{1}{1+ |\xi |^2}. \end{aligned}$$

In Sect. 4, we need the following two results.

#### Lemma 2.5

For $d\in \{ 1,2\}$, let $\gamma _0$ satisfy the assumption (i) on page 2 and let $\mu _p$ be a symmetric measure on $({\mathbb {R}}^{d})^p$, for some integer $p\ge 1$. Then, with $0< s\le t$ and $\Delta _p(t)= \{ \pmb {s_p}\in {\mathbb {R}}_+^p: t =s_0> s_1> \cdots> s_p > 0 \}$,

$$\begin{aligned}&\sum _{\sigma \in {\mathfrak {S}}_p} \int _{\Delta _p(t)} d\pmb {s_p} \int _{[0,s]^p} d\pmb {{\tilde{s}}_p} {\mathbf {1}}_{\{ s> {\tilde{s}}_{\sigma (1)> \cdots> {\tilde{s}}_{\sigma (p)} >0 } \}} \left( \prod _{j=1}^p \gamma _0(s_j - {\tilde{s}}_{j} ) \right) \int _{{\mathbb {R}}^{pd}} \mu _p(d\pmb {\xi _p}) \\&\quad \times g(s_1, \xi _1, \dots , s_p, \xi _p) g({\tilde{s}}_{\sigma (1)}, \xi _{\sigma (1)}, \dots , {\tilde{s}}_{\sigma (p)}, \xi _{\sigma (p)}) \\&\quad \le \Gamma _t^p \int _{\Delta _p(t)} d\pmb {s_p} \int _{{\mathbb {R}}^{pd}} \mu _p(d\pmb {\xi _p}) g(s_1, \xi _1, \dots , s_p, \xi _p)^2, \quad \text {with}~ \Gamma _t : = \int _{-t}^t \gamma _0(a)da, \end{aligned}$$

for any measurable function $g: ({\mathbb {R}}_+\times {\mathbb {R}}^d)^p\rightarrow {\mathbb {R}}_+$ for which the above integral is finite.

#### Proof

After applying $|ab|\le \frac{a^2+b^2}{2}$ and using the symmetry of $\mu _p$, we have that the left-hand side quantity is bounded by

2.31 $$\begin{aligned}&\frac{1}{2} \sum _{\sigma \in {\mathfrak {S}}_p} \int _{\Delta _p(t)} d\pmb {s_p} \int _{[0,s]^p} d\pmb {{\tilde{s}}_p} {\mathbf {1}}_{\{ s> {\tilde{s}}_{\sigma (1)> \cdots> {\tilde{s}}_{\sigma (p)} >0 } \}} h(\pmb {s_p}) \prod _{j=1}^p \gamma _0(s_j - {\tilde{s}}_{j} ) \ \end{aligned}$$

2.32 $$\begin{aligned}&\quad + \frac{1}{2} \sum _{\sigma \in {\mathfrak {S}}_p}\int _{\Delta _p(t)} d\pmb {s_p} \int _{[0,s]^p} d\pmb {{\tilde{s}}_p} {\mathbf {1}}_{\{ s> {\tilde{s}}_{\sigma (1)> \cdots> {\tilde{s}}_{\sigma (p)} >0 } \}} h\big ( {\tilde{s}}_{\sigma (1)}, ..., {\tilde{s}}_{\sigma (p)} \big ) \prod _{j=1}^p \gamma _0(s_j -{\tilde{s}}_{j} ) \end{aligned}$$

with

$$\begin{aligned} h(s_1, \dots , s_p):= {\left\{ \begin{array}{ll} {\displaystyle \int _{{\mathbb {R}}^{pd}} \mu _p(d\pmb {\xi _p} ) g(s_1, \xi _1, \dots , s_p, \xi _p)^2, } \quad &{}\text {for }\pmb {s_p}\in \Delta _p(t) \\ 0, &{} \text {otherwise.} \end{array}\right. } \end{aligned}$$

Putting ${\mathcal {I}}_s(s_1, \dots , s_p) := {\mathbf {1}}_{\{ s> s_1> \cdots> s_p>0 \}}$ and letting $\widetilde{{\mathcal {I}}}_s(s_1, \dots , s_p)$ be its canonical symmetrization (so that $\big \vert \widetilde{{\mathcal {I}}}_s\big \vert \le (p!)^{-1}$), we can rewrite the term in (2.31) as

$$\begin{aligned}&\frac{p!}{2} \int _{\Delta _p(t)}\int _{[0,s]^p} d\pmb {s_p} d\pmb {{\tilde{s}}_p} h(\pmb {s_p}) \widetilde{{\mathcal {I}}}_s(\pmb {{\tilde{s}}_p} ) \prod _{j=1}^p \gamma _0(s_j-{\tilde{s}}_j) \\&\quad \le \frac{1}{2} \int _{\Delta _p(t)}\int _{[0,s]^p} d\pmb {s_p} d\pmb {{\tilde{s}}_p} h(\pmb {s_p}) \prod _{j=1}^p \gamma _0(s_j-{\tilde{s}}_j) \\&\quad \le \frac{1}{2}\Gamma _t^p \int _{\Delta _p(t)} d\pmb {s_p} h(\pmb {s_p}), \end{aligned}$$

using also the bound $\sup \{ \int _0^s \gamma _0(r-r') dr' : r\in [0,t] \}\le \Gamma _t$. For the other term (2.32), we argue in the same way: With $({\mathcal {I}}_s \cdot h)(s_1, ... , s_p) ={\mathcal {I}}_s(s_1, \dots , s_p) h(s_1, ... , s_p)$, we rewrite the term (2.32) as

$$\begin{aligned}&\frac{p!}{2} \int _{[0,t]^p} d\pmb {s_p} \int _{[0,s]^p} d\pmb {{\tilde{s}}_p} {\mathcal {I}}_t(\pmb {s_p}) \times \widetilde{({\mathcal {I}}_s \cdot h)}(\pmb {{\widetilde{s}}_p}) \prod _{j=1}^p \gamma _0(s_j - {\tilde{s}}_{j} ) = \frac{p!}{2} \big \langle {\mathcal {I}}_t, \widetilde{{\mathcal {I}}_s \cdot h} \big \rangle _{{\mathcal {H}}^{\otimes p}} \\&\quad =\frac{p!}{2} \big \langle \widetilde{ {\mathcal {I}}_t}, {\mathcal {I}}_s \cdot h \big \rangle _{{\mathcal {H}}^{\otimes p}} \le \frac{1}{2} \int _{[0,t]^p} d\pmb {t_p} \int _{\Delta _p(s)} h( \pmb {{\widetilde{s}}_p}) \prod _{j=1}^p\gamma _0(s_j-{\widetilde{s}}_j) \\&\quad \le \frac{1}{2} \Gamma _t^p \int _{\Delta _p(s)} d\pmb {s_p} h(\pmb {s_p}), \end{aligned}$$

since $h\ge 0$ and $\big \vert \widetilde{{\mathcal {I}}}_t\big \vert \le (p!)^{-1}$. This concludes the proof. $\square$

#### Lemma 2.6

For $d\in \{ 1,2\}$ let $\gamma , \mu$ satisfy the assumption (ii) on page 2. Then, for any *nonnegative* function $h\in {\mathcal {P}}_0\cap L^1({\mathbb {R}}^d)$,

$$\begin{aligned} \sup _{z\in {\mathbb {R}}^d} \int _{{\mathbb {R}}^d}\mu (d\xi ) | {\mathcal {F}}h(\xi +z) |^2 \le \int _{{\mathbb {R}}^d}\mu (d\xi ) | {\mathcal {F}}h(\xi ) |^2. \end{aligned}$$

As a consequence, for any integer $p\ge 1$ and $w_1, \dots , w_p\in [0,t]$,

2.33 $$\begin{aligned} \sup _{\pmb {w_p}\in [0,t]^p }\sup _{\pmb {z_p}\in {\mathbb {R}}^{dp}} \int _{{\mathbb {R}}^{dp}} \mu (d\pmb {\xi _p}) \prod _{j=1}^{p} \big \vert {\widehat{G}}_{w_j}(\xi _j + z_j ) \big \vert ^2 \le \left( 2(t^2\vee 1) \int _{{\mathbb {R}}^d} \frac{\mu (d\xi )}{1+ |\xi |^2} \right) ^p.\nonumber \\ \end{aligned}$$

#### Proof

Since $h \ge 0$, using the fact that ${\mathcal {F}}h(\xi +z) = {\mathcal {F}}(e^{-iz \cdot } h)(\xi )$ together with $|e^{-iz (x +y)}|=1$, we get

$$\begin{aligned} \int _{{\mathbb {R}}^d} \mu (d\xi ) \big \vert {\mathcal {F}}h (\xi + z ) \big \vert ^2&= \int _{{\mathbb {R}}^{2d}} e^{-iz (x +y)} h(x) h(y) \gamma (x-y) dxdy \\&\le \int _{{\mathbb {R}}^{2d}} h(x) h(y) \gamma (x-y) dxdy, \end{aligned}$$

which is exactly $\int _{{\mathbb {R}}^d} \mu (d\xi ) \big \vert {\mathcal {F}}h(\xi ) \big \vert ^2.$ In particular, by (2.30),

$$\begin{aligned} \sup _{z\in {\mathbb {R}}^d}\int _{{\mathbb {R}}^d} \mu (d\xi ) \big \vert {\widehat{G}}_s (\xi + z ) \big \vert ^2 \le \int _{{\mathbb {R}}^d} \mu (d\xi ) \big \vert {\widehat{G}}_s (\xi ) \big \vert ^2 \le 2(s^2\vee 1) \int _{{\mathbb {R}}^d} \frac{\mu (d\xi )}{1+ |\xi |^2}, \end{aligned}$$

which is finite due to Dalang’s condition (1.2). Applying this inequality several times yields

$$\begin{aligned}&\int _{{\mathbb {R}}^{dp}} \mu (d\pmb {\xi _p}) \prod _{j=1}^{p} \big \vert {\widehat{G}}_{w_j}(\xi _j + z_j ) \big \vert ^2 \le \left( 2(t^2\vee 1) \int _{{\mathbb {R}}^d} \frac{\mu (d\xi )}{1+ |\xi |^2} \right) ^p, \end{aligned}$$

which is a uniform bound over $(\pmb {z_p}, \pmb {w_p})\in {\mathbb {R}}^{dp}\times [0,t]^p$. $\square$

## $L^p$ estimates for Malliavin derivatives

This section is mainly devoted to the proof of Theorem 1.3. The proof will be done in several steps organized in Sects. 3.1, 3.2, 3.3, 3.4 and 3.5. In Sect. 3.6, we record a few consequences of Theorem  1.3 that will be used in the proof of Theorem 1.10 in Sect. 5.

### Step 1: Preliminaries

Let us first introduce some handy notation. Recall that for $\pmb {t_n}:=(t_1,\ldots ,t_n)$ and $\pmb {x_n}:=(x_1,\ldots ,x_n)$, we defined in (1.8)

$$\begin{aligned} f_{t,x,n}(\pmb {t_n},\pmb {x_n})=G_{t-t_{1}}(x-x_{1}) G_{t_1-t_2}(x_1-x_2) \cdots G_{t_{n-1}-t_n}(x_{n-1}-x_n), \end{aligned}$$

with the convention (1.6), and we denote by ${\widetilde{f}}_{t,x,n}$ the symmetrization of $f_{t,x,n}$; see (1.9). We treat the time-space variables $(t_i,x_i)$ as one coordinate and we write

$$\begin{aligned} f_{t,x,n}(r,z;\pmb {t_{n-1}},\pmb {x_{n-1}}) := f_{t,x,n}(r,z, t_1,x_1, \ldots , t_{n-1}, x_{n-1}) \end{aligned}$$

as in Notation **A**-(3). Recall that the solution *u*(*t*, *x*) has the Wiener chaos expansion

$$\begin{aligned} u(t,x)=1+ \sum _{n=1}^ \infty I_n(f_{t,x,n}), \end{aligned}$$

where the kernel $f_{t,x,n}$ is not symmetric and in this case, by definition, $I_n(f_{t,x,n})= I_n\big ({\widetilde{f}}_{t,x,n} \big )$.

Our first goal is to show that, for any fixed $(r,z) \in [0,t] \times {\mathbb {R}}^d$ and for any $p\in [2,\infty )$, the series

3.1 $$\begin{aligned} \sum _{n\ge 1} n I_{n-1}\big ( {\widetilde{f}}_{t,x,n}(r,z; \bullet ) \big ) \end{aligned}$$

converges in $$L^p(\Omega )$$, and the sum, denoted by $$D_{r,z}u(t,x) $$, satisfies the $$L^p$$ estimates (1.11).

The first term of the series (3.1) is ${\widetilde{f}}_{t,x,1}(r,z)=G_{t-r}(x-z)$. In general, for any $n\ge 1$,

3.2 $$\begin{aligned} {\widetilde{f}}_{t,x,n}(r,z;\bullet ) = \frac{1}{n} \sum _{j=1}^n h^{(j)}_{t,x,n}(r,z;\bullet ), \end{aligned}$$

where $h^{(j)}_{t,x,n}(r,z;\bullet )$ is the symmetrization of the function $(\pmb {t_{n-1}},\pmb {x_{n-1}})\rightarrow f^{(j)}_{t,x,n}(r,z; \pmb {t_{n-1}},\pmb {x_{n-1}})$, which is obtained from $f_{t,x,n}$ by placing *r* on position *j* among the time instants, and *z* on position *j* among the space points: With the convention (1.6),

3.3 $$\begin{aligned}&f^{(j)}_{t,x,n}(r,z; \pmb {t_{n-1}},\pmb {x_{n-1}})\nonumber \\&\quad =G_{t-t_1}(x-x_1) \cdots G_{t_{j-1}-r}(x_{j-1}\!-\!z)G_{r-t_j}(z-x_j) \cdots G_{t_{n-2}\!-\!t_{n-1}}(x_{n-2}\!-\!x_{n-1}). \end{aligned}$$

That is,

3.4 $$\begin{aligned} f^{(j)}_{t,x,n}(r,z; \bullet ) = f_{t,x,j}^{(j)}(r,z;\bullet )\otimes f_{r,z,n-j}, \end{aligned}$$

with $$ f_{r,z,1}=1$$. For example, $$f^{(1)}_{t,x,1}(r,z; \bullet ) = G_{t-r}(x-z)$$ and $$f^{(1)}_{t,x,n}(r,z; \pmb {t_{n-1}}, \pmb {x_{n-1}} )= G_{t-r}(x-z) f_{r,z,n-1}( \pmb {t_{n-1}}, \pmb {x_{n-1}} )$$. By the definition of the symmetrization,

3.5 $$\begin{aligned}&h^{(j)}_{t,x,n}(r,z;\pmb {t_{n-1}},\pmb {x_{n-1}})\nonumber \\&\quad =\frac{1}{(n-1)!}\sum _{\sigma \in {\mathfrak {S}}_{n-1}}f_{t,x,n}^{(j)}(r,z;t_{\sigma (1)},x_{\sigma (1)}, \ldots ,t_{\sigma (n-1)},x_{\sigma (n-1)}). \end{aligned}$$

Similarly, for $\pmb {s_m}\in [0,t]^m$ and $\pmb {y_m}\in {\mathbb {R}}^{dm}$, and for any $p\in [2,\infty )$, we will show that

3.6 $$\begin{aligned} D^m_{\pmb {s_m}, \pmb {y_m} }u(t,x) := \sum _{n\ge m} \frac{n!}{(n-m)!} I_{n-m}\big ( {\widetilde{f}}_{t,x,n}(\pmb {s_m}, \pmb {y_m}; \bullet ) \big ) \end{aligned}$$

converges in $L^p(\Omega )$. Note that if the series (3.6) converges in $L^p(\Omega )$, we can see that almost surely, the function

$$\begin{aligned} (\pmb {s_m}, \pmb {y_m} ) \mapsto D^m_{\pmb {s_m}, \pmb {y_m} }u(t,x) \end{aligned}$$

is *symmetric*, meaning that for any $\sigma \in {\mathfrak {S}}_m$,

$$\begin{aligned} D_{s_1, y_1} D_{s_2, y_2} \cdots D_{s_m, y_m} u(t,x) = D_{s_{\sigma (1)}, y_{\sigma (1)}} D_{s_{\sigma (2)}, y_{\sigma (2)}} \cdots D_{s_{\sigma (m)} , y_{\sigma (m)}} u(t,x). \end{aligned}$$

*From now on*, we assume $t>s_1> ...> s_m>0$ without losing any generality. Note that like (3.2), we can write

3.7 $$\begin{aligned} \frac{n!}{(n-m)!} {\widetilde{f}}_{t,x,n}(\pmb {s_m}, \pmb {y_m}; \bullet ) = \sum _{\pmb {i_m}\in \Delta _{n,m}} h^{(\pmb {i_m})}_{t,x,n}(\pmb {s_m}, \pmb {y_m}; \bullet ), \end{aligned}$$

where $\pmb {i_m}\in \Delta _{n,m}$ means $1 \le i_1< i_2< \cdots < i_m \le n$ and $h^{(\pmb {i_m})}_{t,x,n}(\pmb {s_m}, \pmb {y_m}; \bullet )$ is the symmetrization of the function $f^{(\pmb {i_m})}_{t,x,n}(\pmb {s_m}, \pmb {y_m}; \bullet )$ that is defined by

3.8 $$\begin{aligned} f^{(\pmb {i_m})}_{t,x,n}(\pmb {s_m}, \pmb {y_m}; \bullet )&= f^{(i_1)}_{t,x,i_1}(s_1, y_1; \bullet ) \otimes f^{(i_2-i_1)}_{s_1,y_1,i_2-i_1}(s_2, y_2; \bullet ) \otimes \cdots \otimes \nonumber \\&\quad f^{(i_m-i_{m-1})}_{s_{m-1},y_{m-1}, i_m- i_{m-1}}(s_m, y_m; \bullet ) \otimes f_{s_m, y_m, n-i_m}, \end{aligned}$$

which is a generalization of (3.4).

### Step 2: Reduction to white noise in time

Let $\dot{{\mathfrak {X}}}$ denote the Gaussian noise that is white in time and has the same spatial correlation as *W* and let $\{{\mathfrak {X}}(f): f\in {\mathcal {H}}_0\}$ denote the resulting isonormal Gaussian process; see Sect. 2.1.

For any $p\in [2,\infty )$, we deduce from (3.6) and (3.7) that

$$\begin{aligned} \big \Vert D^m_{\pmb {s_m}, \pmb {y_m} }u(t,x) \big \Vert _p \!&\le \! \sum _{n\ge m} \left\| I_{n-m}\left( \sum _{\pmb {i_m}\in \Delta _{n,m} } h^{(\pmb {i_m})}_{t,x,n}(\pmb {s_m}, \pmb {y_m}; \bullet ) \right) \right\| _p \, \text {by triangle inequality} \\&\le \! \sum _{n\ge m} (p-1)^{\frac{n-m}{2}} \left\| I_{n-m}\left( \sum _{\pmb {i_m}\in \Delta _{n,m}} \!h^{(\pmb {i_m})}_{t,x,n}(\pmb {s_m}, \pmb {y_m}; \bullet ) \right) \right\| _2 \, \text {by }(2.15). \end{aligned}$$

The function $\sum _{\pmb {i_m}\in \Delta _{n,m}} h^{(\pmb {i_m})}_{t,x,n}(\pmb {s_m}, \pmb {y_m}; \bullet )$ vanishes outside $\big ([0,t]\times {\mathbb {R}}^d\big )^{n-m}$, thus we deduce from (2.13) that

$$\begin{aligned}&\left\| I_{n-m}\left( \sum _{\pmb {i_m}\in \Delta _{n,m}} h^{(\pmb {i_m})}_{t,x,n}(\pmb {s_m}, \pmb {y_m}; \bullet ) \right) \right\| _2^2 = (n-m)! \left\| \sum _{\pmb {i_m}\in \Delta _{n,m}} h^{(\pmb {i_m})}_{t,x,n}(\pmb {s_m}, \pmb {y_m}; \bullet )\right\| _{{\mathcal {H}}^{\otimes ( n-m)}}^2 \\&\quad \le \Gamma _t^{n-m} (n-m)! \left\| \sum _{\pmb {i_m}\in \Delta _{n,m}} h^{(\pmb {i_m})}_{t,x,n}(\pmb {s_m}, \pmb {y_m}; \bullet )\right\| _{{\mathcal {H}}_0^{\otimes (n-m)}}^2 \\&\quad = \Gamma _t^{n-m} \left\| I^{{\mathfrak {X}}}_{n-m}\left( \sum _{\pmb {i_m}\in \Delta _{n,m}} h^{(\pmb {i_m})}_{t,x,n}(\pmb {s_m}, \pmb {y_m}; \bullet ) \right) \right\| _2^2. \end{aligned}$$

Therefore, we get

3.9 $$\begin{aligned} \big \Vert D^m_{\pmb {s_m}, \pmb {y_m} }u(t,x) \big \Vert _p&\le \sum _{n\ge m} \big [ (p-1) \Gamma _t\big ]^{\frac{n-m}{2}} \left\| \sum _{\pmb {i_m}\in \Delta _{n,m}} I^{{\mathfrak {X}}}_{n-m} \big ( f^{(\pmb {i_m})}_{t,x,n}(\pmb {s_m}, \pmb {y_m}; \bullet )\big ) \right\| _2. \end{aligned}$$

This leads to

3.10 $$\begin{aligned} \big \Vert D^m_{\pmb {s_m}, \pmb {y_m} }u(t,x) \big \Vert _p \le \sum _{n\ge m} \big [ (p-1) \Gamma _t\big ]^{\frac{n-m}{2}} \sqrt{{\mathcal {Q}}_{m,n}}, \end{aligned}$$

with

3.11 $$\begin{aligned} {\mathcal {Q}}_{m,n} :&={\mathbb {E}}\left[ \left( \sum _{\pmb {i_m}\in \Delta _{n,m}}I^{{\mathfrak {X}}}_{n-m} \big ( f^{(\pmb {i_m})}_{t,x,n}(\pmb {s_m}, \pmb {y_m}; \bullet )\big ) \right) ^2 \right] \nonumber \\&\le \left( {\begin{array}{c}n\\ m\end{array}}\right) \sum _{\pmb {i_m}\in \Delta _{n,m}} {\mathbb {E}}\left( I^{{\mathfrak {X}}}_{n-m} \big ( f^{(\pmb {i_m})}_{t,x,n}(\pmb {s_m}, \pmb {y_m}; \bullet )\big )^2 \right) . \end{aligned}$$

The product formula (2.14) and the decomposition (3.8) yield, with $(i_0, s_0, y_0)=(0, t,x)$,

3.12 $$\begin{aligned}&{\mathcal {Q}}_{m,n} \le \left( {\begin{array}{c}n\\ m\end{array}}\right) \nonumber \\&\quad \sum _{\pmb {i_m}\in \Delta _{n,m}} {\mathbb {E}}\left( I^{{\mathfrak {X}}}_{n - i_{m}}\big ( f_{s_m, y_m, n-i_m} \big )^2 \prod _{j=1}^m I^{{\mathfrak {X}}}_{i_j - i_{j-1}-1} \Big ( f^{ (i_j- i_{j-1}) }_{s_{j-1}, y_{j-1}, i_j-i_{j-1}}(s_j, y_j;\bullet ) \Big )^2 \right) \nonumber \\&\quad = \left( {\begin{array}{c}n\\ m\end{array}}\right) \sum _{\pmb {i_m}\in \Delta _{n,m}} \big \Vert I^{{\mathfrak {X}}}_{n - i_{m}}\big ( f_{s_m, y_m, n-i_m} \big )\big \Vert ^2_2 \nonumber \\&\quad \times \prod _{j=1}^m\Big \Vert I^{{\mathfrak {X}}}_{i_j - i_{j-1}-1} \Big ( f^{ (i_j- i_{j-1}) }_{s_{j-1}, y_{j-1}, i_j-i_{j-1}}(s_j, y_j;\bullet ) \Big ) \Big \Vert ^2_2, \end{aligned}$$

where the last equality is obtained by using the independence among the random variables inside the expectation. It remains to estimate two typical terms:

3.13 $$\begin{aligned} \big \Vert I^{{\mathfrak {X}}}_j(f_{r,z,j}) \Vert _2^2 \quad \mathrm{and}\quad \Big \Vert I^{{\mathfrak {X}}}_{j-1}(f^{(j)}_{t,x,j}(r,z;\bullet ) \big ) \Big \Vert _2^2 ~\text {for }1\le j\le n\text { and }t>r. \end{aligned}$$

The first term in (3.13) can be estimated as follows. Using Fourier transform in space (see (2.29)), we have, with $t_0=r$,

3.14 $$\begin{aligned} \big \Vert I^{{\mathfrak {X}}}_j(f_{r,z,j}) \Vert _2^2&= j! \big \Vert {\widetilde{f}}_{r,z,j} \big \Vert _{{\mathcal {H}}_0^{\otimes j}}^2 = \int _{[0,r]^j} \big \Vert f_{r,z,j}(\pmb {t_j}, \bullet ) \big \Vert _0^2 d\pmb {t_j} \nonumber \\&= \int _{r>t_1> \cdots> t_j>0} \int _{{\mathbb {R}}^{dj}}\big \vert {\mathcal {F}} f_{r,z,j}(\pmb {t_j}, \pmb {\xi _j}) \big \vert ^2 \mu (d\pmb {\xi _j}) d\pmb {t_j} \nonumber \\&= \int _{r>t_1> \cdots> t_j >0} \left( \int _{{\mathbb {R}}^{dj}} \prod _{k=0}^{j-1} \big \vert {\mathcal {F}}G_{t_{k} \!-\! t_{k+1}}(\xi _{k+1} \!+\!\cdots \!+\! \xi _j ) \big \vert ^2 \mu (d\xi _k) \right) d\pmb {t_j}. \end{aligned}$$

By Lemma 2.6,

3.15 $$\begin{aligned} \big \Vert I^{{\mathfrak {X}}}_j(f_{r,z,j}) \Vert _2^2&\le \frac{C^j}{j!}, \end{aligned}$$

where $$C= 2(t^2+1) \int _{{\mathbb {R}}^d} ( 1+ |\xi |^2)^{-1}\mu (d\xi )$$.

#### Remark 3.1

By the arguments that lead to (3.9), we can also get, for any $p\in [2,\infty )$,

$$\begin{aligned} \big \Vert u(t,x) \big \Vert _p \le 1 + \sum _{n\ge 1} \big \Vert I_n(f_{t,x,n} )\big \Vert _p \le 1 + \sum _{n\ge 1} \big [ (p-1) \Gamma _t\big ] ^{n/2} \big \Vert I^{{\mathfrak {X}}}_n(f_{t,x,n} )\big \Vert _2 \end{aligned}$$

and then the estimate (3.15) implies $u(t,x)\in L^p(\Omega )$. Moreover,

3.16 $$\begin{aligned} \sup _{(s,y)\in [0,t]\times {\mathbb {R}}^d } \Vert u(s,y) \Vert _p <+\infty ~ \text {for any }t\in {\mathbb {R}}_+. \end{aligned}$$

This is done under the Dalang’s condition (1.2) only and the case $p=2$ provides another proof of [3, Theorem 4.4] when $d=1,2$.

In what follows, we estimate the second term in (3.13) separately for the cases $d=1$ and $d=2$. As usual, we will use *C* to denote an immaterial constant that may vary from line to line.

#### Estimation of $\Big \Vert I^{{\mathfrak {X}}}_{j-1}(f^{(j)}_{t,x,j}(r,z;\bullet ) \big ) \Big \Vert _2^2$ when $d=1$

When $d=1$, $G_t(x) = \frac{1}{2} {\mathbf {1}}_{\{|x| <t \}}$. For $j=1$, $I^{{\mathfrak {X}}}_{j-1}(f^{(j)}_{t,x,j}(r,z;\bullet ) \big )=G_{t-r}(x-z)$ with the convention (1.6). For $j\ge 2$, it follows from the (modified) isometry property (2.8) that

$$\begin{aligned} \Big \Vert I^{{\mathfrak {X}}}_{j-1}(f^{(j)}_{t,x,j}(r,z;\bullet ) \big ) \Big \Vert _2^2&= (j-1)! \Big \Vert h^{(j)}_{t,x,j}(r,z;\bullet ) \Big \Vert _{{\mathcal {H}}_0^{\otimes (j-1)}}^2 \\&= \int _{[r,t]^{j-1}} \big \Vert f^{(j)}_{t,x,j}(r,z; \pmb {t_{j-1}}, \bullet ) \big \Vert _{0}^2 d\pmb {t_{j-1}}, \end{aligned}$$

where we recall that $h^{(j)}_{t,x,j}(r,z;\bullet )$ is the symmetrization of $f^{(j)}_{t,x,j}(r,z;\bullet )$; see (3.5). Then, taking advantage of the simple form of $G_t(x)$ for $d=1$, we get

$$\begin{aligned} 0\le f^{(j)}_{t,x,j}(r,z; \pmb {t_{j-1}}, \bullet ) \le \frac{1}{2}{\mathbf {1}}_{\{ |x - z| < t-r\}} f_{t,x,j-1}(\pmb {t_{j-1}}, \bullet ), \end{aligned}$$

from which we further get

3.17 $$\begin{aligned} \Big \Vert I^{{\mathfrak {X}}}_{j-1}(f^{(j)}_{t,x,j}(r,z;\bullet ) \big ) \Big \Vert _2^2&\le G^2_{t-r}(x-z) \int _{[r,t]^{j-1}} \big \Vert f_{t,x,j-1}(\pmb {t_{j-1}}, \bullet ) \big \Vert _0^2 d\pmb {t_{j-1}} \nonumber \\&\le \frac{C^{j-1}}{(j-1)!} G^2_{t-r}(x-z), \end{aligned}$$

where the last inequality follows from (3.15) and (3.14).

#### Estimation of $\Big \Vert I^{{\mathfrak {X}}}_{j-1}(f^{(j)}_{t,x,j}(r,z;\bullet ) \big ) \Big \Vert _2^2$ when $d=2$

Let *q* be defined as in (2.20) and (2.23) and we fix such a *q* *throughout this subsection*. For $j=1$, $I^{{\mathfrak {X}}}_{j-1}(f^{(j)}_{t,x,j}(r,z;\bullet ) \big )=G_{t-r}(x-z)$ with the convention (1.6). For $j\ge 2$, we begin with

$$\begin{aligned} \Big \Vert I^{{\mathfrak {X}}}_{j-1}(f^{(j)}_{t,x,j}(r,z;\bullet ) \big ) \Big \Vert _2^2&= \int _{[r,t]^{j-1}} \big \Vert f^{(j)}_{t,x,j}(r,z; \pmb {t_{j-1}}, \bullet ) \big \Vert _0^2 d\pmb {t_{j-1}}, \\&\le C^{j-1}\!\! \int _{t> t_1> \cdots>t_{j-1}>r} \big \Vert f^{(j)}_{t,x,j}(r,z; \pmb {t_{j-1}}, \bullet ) \big \Vert _{L^{2q}({\mathbb {R}}^{2j-2}) }^2 d\pmb {t_{j-1}} \\&=C^{j-1} {\mathcal {T}}_j, \end{aligned}$$

where we applied Lemma 2.3 for the inequality above^10^ and we denote

3.18 $$\begin{aligned} {\mathcal {T}}_j := \int _{ t>t_1>\cdots>t_{j-1}>r} d\pmb {t_{j-1}} \left( \! \int _{{\mathbb {R}}^{2(j-1)}} G^{2q}_{t-t_1}(x-x_1) \! \cdots \! G^{2q}_{t_{j-1} -r}(x_{j-1} \!-\!z) d \pmb {x_{j-1}}\! \right) ^{ 1/q}\!. \end{aligned}$$

Note that we can choose *C* to depend only on $(t,\gamma , q)$ and be increasing in *t*.

**Case** $j=2$. In this case, we deduce from Lemma  2.4 and (2.27) that

3.19 $$\begin{aligned} {\mathcal {T}}_2 \!=\! \int _r^t dt_1 ( G^{2q}_{t-t_1} *G^{2q}_{t_1-r} )^{1/q} (x\!-\!z) \!\le \! CG_{t-r}^{2-\frac{1}{q}}(x-z) \le C G^2_{t-r} (x-z).\quad \end{aligned}$$

**Case** $j\ge 3$. In this case, we use Minkowski inequality with respect to the norm in $L^{1/q}( [t_2,t] ,dt_1)$ in order to get

$$\begin{aligned} {\mathcal {T}}_{j}&\le \int _{ t>t_2>\cdots>t_{j-1}>r} \Bigg ( \int _{{\mathbb {R}}^{2(j-2)}} \left[ \int _{t_2} ^t \big ( G_{t-t_1}^{2q} *G_{t_1-t_2}^{2q}\big )^{1/q}(x-x_2) dt_1 \right] ^q\\&\quad \times G^{2q}_{t_2-t_3}(x_2-x_3) \cdots G^{2q}_{t_{j-1} -r}(x_{j-1} -z) dx_2 \cdots dx_{j-1} \Bigg ) ^{1/q} dt_2 \cdots dt_{j-1}. \end{aligned}$$

Applying Lemma 2.4 yields

3.20 $$\begin{aligned} {\mathcal {T}}_{j}&\le A_q \int _{t> t_2> \cdots> t_{j-1}>r} (t-t_2)^{\frac{1}{q}-1} \Bigg ( \int _{{\mathbb {R}}^{2(j-2)}} G^{2q-1} _{t-t_2}(x-x_2) \nonumber \\&\quad \times G^{2q}_{t_2-t_3}(x_2-x_3) \cdots G^{2q}_{t_{j-1} -r}(x_{j-1} -z) dx_2 \cdots dx_{j-1} \Bigg ) ^{1/q} dt_2 \cdots dt_{j-1}. \end{aligned}$$

If $j=3$, we have

$$\begin{aligned} {\mathcal {T}}_3&\le A_q \int _r^t (t-t_2)^{\frac{1}{q}-1} \Bigg ( \int _{{\mathbb {R}}^{2}} G^{2q-1} _{t-t_2}(x-x_2) G^{2q}_{t_2-r}(x_2-z) dx_2 \Bigg ) ^{ 1/q} dt_2. \end{aligned}$$

Owing to (2.27), we can bound $$G^{2q-1} _{t-t_2}(x-x_2) $$ by $$(2\pi ) (t-t_2)G^{2q} _{t-t_2}(x-x_2) $$, and then we apply again Lemma 2.4 and (2.27) to conclude that

3.21 $$\begin{aligned} {\mathcal {T}}_3 \le A_q^{2} (2\pi )^{\frac{1}{q}} (t-r)^{\frac{3}{q}-2} G_{t-r}^{2-\frac{1}{q}}(x-z) \le C G^2_{t-r}(x-z). \end{aligned}$$

For $j\ge 4$, we continue with the estimate (3.20). We can first apply Minkowski inequality with respect to the norm $L^{1/q}\big ( [t_4, t_2], dt_3\big )$ and then apply Lemma 2.4 to obtain

3.22 $$\begin{aligned} {\mathcal {T}}_{j}&\le A_q^{2} \int _{t> t_2>t_4> \cdots> t_{j-1}> r} dt_2 dt_4 \cdots dt_{j-1} (t-t_2)^{\frac{1}{q}-1}(t_2-t_4)^{\frac{1}{q}-1}\nonumber \\&\quad \Bigg ( \int _{{\mathbb {R}}^{2(j-3)}} G^{2q-1} _{t-t_2}(x-x_2) \nonumber \\&\quad \times G^{2q-1} _{t_2-t_4} (x_2-x_4) G^{2q}_{t_4-t_5}(x_4-x_5) \cdots G^{2q}_{t_{j-1} -r}(x_{j-1} -z) dx_2 dx_4 \cdots dx_{j-1} \Bigg ) ^{1/q}. \end{aligned}$$

Note that

$$\begin{aligned} G^{2q-1} _{t-t_2}(x-x_2) G^{2q-1} _{t_2-t_4}(x_2-x_4) \le {\mathbf {1}}_{\{ |x-x_4 | \le t-t_4\}} G^{2q-1} _{t-t_2}(x-x_2) G^{2q-1} _{t_2-t_4}(x_2-x_4). \end{aligned}$$

Then, by Cauchy-Schwarz inequality and (2.26), we can infer that

$$\begin{aligned} \int _{{\mathbb {R}}^2} G^{2q-1} _{t-t_2}(x\!-\!x_2) G^{2q-1} _{t_2-t_4} (x_2-x_4) dx_2 \!&\le \! {\mathbf {1}}_{\{ |x-x_4 | \le t-t_4\}} \Vert G^{2q-1} _{t-t_2} \Vert _{L^2({\mathbb {R}}^2)} \Vert G^{2q-1} _{t_2-t_4} \Vert _{L^2({\mathbb {R}}^2)} \\&=c_1 (t-t_2)^{2-2q}(t_2-t_4)^{2-2q} {\mathbf {1}}_{\{ |x-x_4 | \le t-t_4\}}, \end{aligned}$$

where $c_1= \frac{(2\pi )^{3-4q}}{4-4q}$. Thus, substituting this estimate into (3.22), we end up with

$$\begin{aligned}&{\mathcal {T}}_{j} \le A_q^{2} c_1^{1/q} \int _{t> t_2>t_4> \cdots> t_{j-1}> r} dt_2 dt_4 \cdots dt_{j-1} (t-t_2)^{\frac{3}{q}-3}(t_2-t_4) ^{\frac{3}{q}-3} \\&\quad \times \left( \!\int _{{\mathbb {R}}^{2(j-4)}} {\mathbf {1}}_{\{ |x-x_4 | \!\le \! t-t_4\}} G^{2q}_{t_4-t_5}(x_4-x_5) \cdots G^{2q}_{t_{j-1} -r}(x_{j-1} -z) dx_4 \cdots dx_{j-1} \right) ^{1/q}\!. \end{aligned}$$

Focusing on the indicators, the right-hand side of this estimate can be bounded by

$$\begin{aligned}&A_q^{2} c_1^{1/q} {\mathbf {1}}_{\{ |x-z | \le t-r\}} \int _{t> t_2>t_4> \cdots> t_{j-1}> r} dt_2 dt_4 \cdots dt_{j-1} (t-t_2)^{\frac{3}{q}-3}(t_2-t_4) ^{\frac{3}{q}-3}\\&\quad \times \left( \int _{{\mathbb {R}}^{2(j-4)}} G^{2q}_{t_4-t_5}(x_4-x_5) \cdots G^{2q}_{t_{j-1} -r}(x_{j-1} -z) dx_4 \cdots dx_{j-1} \right) ^{1/q}. \end{aligned}$$

For $j=4$, using (2.28), we have

3.23 $$\begin{aligned} {\mathcal {T}}_{4} \le A_q^{2} c_1^{1/q} (t-r)^{\frac{6}{q}-6} {\mathbf {1}}_{\{ |x-z | \le t-r\}} \le C G_{t-r}^{2}(x-z). \end{aligned}$$

Now for $j\ge 5$, we just integrate in each of the variables $x_4, \dots , x_{j-1}$ (with this order) so that, thanks to (2.26), we end up with

$$\begin{aligned}&{\mathcal {T}}_{j} \le A_q^{2} c_1^{1/q} c_2^{j-4} {\mathbf {1}}_{\{ |x-z | \le t-r\}} \int _{t> t_2>t_4> \cdots> t_{j-1}> r} dt_2 dt_4 \cdots dt_{j-1} \\&\times (t-t_2)^{\frac{3}{q}-3}(t_2-t_4) ^{\frac{3}{q}-3} (t_4-t_5)^{\frac{2}{q}-2}\cdots (t_{j-1}-r)^{\frac{2}{q}-2} \quad \text {with }c_2\\&=\left( \dfrac{(2\pi )^{1-2q}}{2-2q}\right) ^{2} \le A_q^{2} c_1^{1/q} c_2^{j-4} \frac{ (t-r)^{j-3} }{(j-3)!} (t-r+1)^{j(\frac{2}{q}-2) } {\mathbf {1}}_{\{ |x-z | \le t-r\}}, \end{aligned}$$

where we used the rough estimate $a^\nu \le (b+1)^{\nu }$ for $0<a\le b$ and $\nu >0$. Thus, using (2.28) we obtain:

3.24 $$\begin{aligned} {\mathcal {T}}_{j} \le \frac{C^{j-3}}{(j-3)!}G^2_{t-r}(x-z) \quad \text {for any}~ j\ge 5. \end{aligned}$$

Hence, combining the estimates (3.19), (3.21), (3.23) and (3.24) and taking into account that $I^{{\mathfrak {X}}}_{0}(f^{(1)}_{t,x,1}(r,z;\bullet ) \big )=G_{r-s}(z-y)$, we can write

$$\begin{aligned} \Big \Vert I^{{\mathfrak {X}}}_{j-1}(f^{(j)}_{t,x,j}(r,z;\bullet ) \big ) \Big \Vert _2^2 \le {\left\{ \begin{array}{ll} C G_{t-r}^2(x-z) &{} \text {for }j=1,2,3,4\\ \dfrac{C^{j} }{(j-3)!} G_{t-r}^2(x-z) &{} \text {for }j\ge 5 \end{array}\right. }, \end{aligned}$$

where the constant $C > 1$ depends on $(t,\gamma , q)$ and is increasing in *t*. For $1\le j \le n$, we obtain the following bound

3.25 $$\begin{aligned} \Big \Vert I^{{\mathfrak {X}}}_{j-1}(f^{(j)}_{t,x,j}(r,z;\bullet ) \big ) \Big \Vert _2^2 \le \ \frac{C^{j}}{j!}n^3G_{t-r}^2(x-z). \end{aligned}$$

### Step 3: Proof of (1.11)

Let us first consider the lower bound in (1.11) for $d\in \{1,2\}$. For $p\in [2,\infty )$, we deduce from the modified isometry (2.8) that

$$\begin{aligned} \big \Vert D^m_{\pmb {s_m}, \pmb {y_m}} u(t,x) \big \Vert _p \ge \big \Vert D^m_{\pmb {s_m}, \pmb {y_m}} u(t,x) \big \Vert _2 \ge m! {\widetilde{f}}_{t,x,m}( \pmb {s_m}, \pmb {y_m}). \end{aligned}$$

Now let us establish the upper bound in (1.11). By symmetry, we can assume $t>s_1> \cdots> s_m >0$. First we consider the case where $d=2$. Recall the definition of ${\mathcal {Q}}_{m,n}$ from (3.11), and then plugging the estimates (3.15) and (3.25) into (3.12) yields, with $(i_0, s_0, y_0) = (0, t,x)$,

$$\begin{aligned} {\mathcal {Q}}_{m,n}&\le \left( {\begin{array}{c}n\\ m\end{array}}\right) \sum _{\pmb {i_m}\in \Delta _{n,m}} \frac{C^{n-i_m}}{(n-i_m)!} \times \prod _{j=1}^m \frac{n^3C^{ i_j - i_{j-1}} }{ (i_j - i_{j-1} )! } G^2_{s_{j-1} - s_j}(y_{j-1} - y_j) \\&\le (2C)^n n^{3m} \left( \sum _{\pmb {i_m}\in \Delta _{n,m}} \frac{1}{ i_1! (i_2-i_1)! \cdots (i_m - i_{m-1})! (n- i_m)! } \right) f^2_{t,x,m}(\pmb {s_m}, \pmb {y_m}), \end{aligned}$$

where we used the rough bound $$\left( {\begin{array}{c}n\\ m\end{array}}\right) \le 2^n$$. The sum in the above display is equal to

$$\begin{aligned} \frac{1}{n!} \sum _{\begin{array}{c} a_1 + ... + a_{m+1} =n \\ a_i\in {\mathbb {N}},\forall i \end{array}} \left( {\begin{array}{c}n\\ a_1, ..., a_{m+1}\end{array}}\right) = \frac{(m+1)^n}{n!}, \end{aligned}$$

by multinomial formula. That is, we can get

$$\begin{aligned} {\mathcal {Q}}_{m,n} \le \frac{\big [C(m+1) \big ]^n n^{3m}}{n!} f^2_{t,x,m}(\pmb {s_m}, \pmb {y_m}), \end{aligned}$$

which, together with the estimate (3.10), implies the upper bound in (1.11), when $d=2$.

The case $d=1$ can be done in the same way by noticing that the bound in (3.17) can be replaced by $n\frac{C^j}{j!} G_{t-r}^2(x-z)$ for $1\le j\le n$. Then, like the estimate for $d=2$, we can get, for $t>s_1> \cdots>s_m>0$,

$$\begin{aligned} {\mathcal {Q}}_{m,n} \le \frac{\big [ C(m+1) \big ]^n n^{m}}{n!} f^2_{t,x,m}(\pmb {s_m}, \pmb {y_m}), \end{aligned}$$

which together with the estimate (3.10) implies the upper bound in (1.11), when $d=1$. This completes the proof of the estimate (1.11).

Notice that the upper bound also shows the convergence in $L^p$ for any $p\in [2,\infty )$ of the series (3.6), for any *fixed* $\pmb {s_m}\in [0,t]^m$ and $\pmb {y_m}\in {\mathbb {R}}^{dm}$.

### Step 4: Existence of a measurable version

We claim that there is a random field *Y* such that $Y(\pmb {s_m}, \pmb {y_m}) = D^m_{\pmb {s_m}, \pmb {y_m}}u(t,x)$ almost surely for almost all $(\pmb {s_m}, \pmb {y_m})\in [0,t]^m\times {\mathbb {R}}^{md}$ and the mapping

$$\begin{aligned} (\omega , \pmb {s_m}, \pmb {y_m})\in \Omega \times [0,t]^m\times {\mathbb {R}}^{md} \longmapsto Y(\omega , \pmb {s_m}, \pmb {y_m})\in {\mathbb {R}}\end{aligned}$$

is jointly measurable. This fact is rather standard and we will sketch the proof only in the case $d=2$. From the explicit form of the kernels $f_{t,x,n}$ given in (1.8), it follows that the mapping

3.26 $$\begin{aligned} (\pmb {s_m}, \pmb {y_m}) \rightarrow {\widetilde{f}}_{t,x,n}(\pmb {s_m}, \pmb {y_m}; \bullet ) \end{aligned}$$

is measurable from $[0,t]^m\times {\mathbb {R}}^{2m}$ to $L^2([0,t]^{n-m} ; L^{2q}({\mathbb {R}}^{2(n-m)}))$. Because

$$\begin{aligned}&L^2([0,t]^{n-m} ; L^{2q}({\mathbb {R}}^{2(n-m)})) \text {is continuously embedded into }{\mathcal {H}}^{\otimes (n-m)}\text { (see }(2.13)\\&\text { and }(2.25)), \end{aligned}$$

we deduce that the map (3.26) is measurable from $[0,t]^m\times {\mathbb {R}}^{2m}$ into ${\mathcal {H}}^{\otimes (n-m)}$. This implies that the mapping

3.27 $$\begin{aligned} (\pmb {s_m}, \pmb {y_m}) \rightarrow I_{n-m}( {\widetilde{f}}_{t,x,n}(\pmb {s_m}, \pmb {y_m}; \bullet ) ) \end{aligned}$$

is measurable from $[0,t]^m\times {\mathbb {R}}^{2m}$ to $L^2(\Omega )$. The upper bound in (1.11) implies that the mapping (3.27) belongs to the space

$$\begin{aligned} L^{2q} ( [0,t]^m \times {\mathbb {R}}^{2m} ; L^2(\Omega )) \subset L^{2q} ( [0,t]^m \times {\mathbb {R}}^{2m} \times \Omega ). \end{aligned}$$

From this, it follows that we can find a measurable modification of the process

$$\begin{aligned} \{ I_{n-m}( {\widetilde{f}}_{t,x,n}(\pmb {s_m}, \pmb {y_m}; \bullet ) )(\omega ): (\omega , \pmb {s_m}, \pmb {y_m})\in \Omega \times [0,t]^m \times {\mathbb {R}}^{2m} \}. \end{aligned}$$

Finally, by standard arguments we deduce the existence of a measurable modification of the series (3.6).

### Step 5: Proof of $u(t,x)\in {\mathbb {D}}^{\infty }$

We have already seen in Remark  3.1 that $u(t,x)\in L^p(\Omega )$ for any $p\in [2,\infty )$. Then, it remains to show that the function $D^m_{\pmb {s_m}, \pmb {y_m}}u(t,x)$ defined as the limit of the series (3.6) coincides with the *m*th Malliavin derivative of *u*(*t*, *x*). To do this, it suffices to show that ${\mathbb {E}}\big [ \Vert D^mu(t,x)\Vert _{{\mathcal {H}}^{\otimes m} }^p \big ] <\infty$ for any $m\ge 1$. By Fubini’ theorem and using the upper bound (1.11), we write

$$\begin{aligned}&\Big ( {\mathbb {E}}\big [ \Vert D^mu(t,x)\Vert _{{\mathcal {H}}^{\otimes m} }^p \big ] \Big )^{2/p} \\&\quad = \left\| \int _{[0,t]^{2m}\times {\mathbb {R}}^{2md}} d\pmb {s_m} d\pmb {s'_m} d\pmb {y_m} d\pmb {y'_m} \big ( D^m_{\pmb {s_m},\pmb {y_m} }u(t,x)\big ) \big ( D^m_{\pmb {s'_m},\pmb {y'_m} }u(t,x)\big )\right. \\&\quad \left. \quad \prod _{j=1}^m\gamma _0(s_j-s_j') \gamma (y_j-y_j') \right\| _{p/2} \\&\quad \le \int _{[0,t]^{2m}\times {\mathbb {R}}^{2md}} d\pmb {s_m} d\pmb {s'_m} d\pmb {y_m} d\pmb {y'_m} \big \Vert D^m_{\pmb {s_m},\pmb {y_m} }u(t,x)\big \Vert _p \big \Vert D^m_{\pmb {s'_m},\pmb {y'_m} }u(t,x)\big \Vert _p\\&\quad \quad \prod _{j=1}^m\gamma _0(s_j-s_j') \gamma (y_j-y_j') \\&\quad \lesssim \big \Vert {\widetilde{f}}_{t,x,m} \big \Vert ^2_{{\mathcal {H}}^{\otimes m}} <\infty . \end{aligned}$$

This shows $u(t,x)\in {\mathbb {D}}^{\infty }$ and completes the proof of Theorem  1.3. $\square$

#### Remark 3.2

When $d=2,p=2,m=1$ and for the cases (a), (b) in Hypothesis $\mathbf{(H1)}$, the upper bound in (1.11) can be proved in a much simpler way for almost all $(r,z)\in [0,t]\times {\mathbb {R}}^2$. Let $v_{\lambda }$ be the solution to the stochastic wave equation

$$\begin{aligned} {\left\{ \begin{array}{ll} {\displaystyle \frac{\partial ^2 v_{\lambda }}{\partial t^2}=\Delta v_{\lambda }+ \lambda v_{\lambda } \dot{{\mathfrak {X}}} } \\ v_\lambda (0,\bullet ) =1, \quad \dfrac{\partial v_\lambda }{\partial t} (0, \bullet ) = 0, \end{array}\right. } \end{aligned}$$

where $\lambda >0$ and $\dot{{\mathfrak {X}}}$ is given as before. This solution has the chaos expansion $v_{\lambda }(t,x)=\sum _{n\ge 0} \lambda ^{n}I_{n}^{{\mathfrak {X}}}(f_{t,x,n})$ and its Malliavin derivative has the chaos expansion

$$\begin{aligned} D_{r,z}v_{\lambda }(t,x)=\sum _{n\ge 1} \lambda ^{n} I_{n-1}^{{\mathfrak {X}}}\left( \sum _{j=1}^{n} h_{t,x,n}^{(j)}(r,z;\bullet )\right) ; \end{aligned}$$

see (3.1) and (3.2). From this, we infer that for any $(\lambda , t, x)\in (0,\infty )^2\times {\mathbb {R}}^2$ and for *almost every* $(r,z)\in [0,t]\times {\mathbb {R}}^2$,

3.28 $$\begin{aligned} \big \Vert D_{r,z}v_{\lambda }(t,x) \big \Vert _2^2 \!=\!\sum _{n\ge 1} (n-1)! \, \lambda ^{2n} \Big \Vert \!\sum _{j=1}^{n}h_{t,x,n}^{(j)}(r,z;\bullet )\!\Big \Vert _{{\mathcal {H}}_0^{\otimes (n-1)}}^2 \!\le \! C_{\lambda ,t,\gamma }G_{t-r}^2(x-z),\nonumber \\ \end{aligned}$$

where $C_{\lambda ,t,\gamma }>0$ is a constant depending on $(\lambda , t, \gamma )$ and is increasing in *t*. The inequality above is due to Theorem 1.3 of [35] for case (a), respectively Theorem 1.2 of [4] for case (b). Therefore,

$$\begin{aligned} \big \Vert D_{r,z}u(t,x) \big \Vert _2^2&=\sum _{n\ge 1} (n-1)! \, \big \Vert \sum _{j=1}^{n}h_{t,x,n}^{(j)}(r,z;\bullet )\big \Vert _{{\mathcal {H}}^{\otimes (n-1)}}^2\\&\le \sum _{n\ge 1} (n-1)! \, \Gamma _t^{n-1} \big \Vert \sum _{j=1}^{n}h_{t,x,n}^{(j)}(r,z;\bullet )\big \Vert _{{\mathcal {H}}_0^{\otimes (n-1)}}^2 ~\text {by }(2.13). \end{aligned}$$

Thus, using (3.28) with $\lambda =\sqrt{\Gamma _t}$, we get $\big \Vert D_{r,z}u(t,x) \big \Vert _2^2 \le C_{\Gamma _t,t,\gamma }G_{t-r}^2(x-z)$.

### Consequences of Theorem 1.3

We will establish two estimates that will be useful in Sect. 5.

#### Corollary 3.3

Let $d=1,2$. Then, for any finite $T>0$,

3.29 $$\begin{aligned} \sup _{(t,x)\in [0,T]\times {\mathbb {R}}^d} \, \sup _{r\in [0,t]} {\mathbb {E}}\Big [ \big \Vert |D_{r,\bullet } u(t,x)| \big \Vert _{0}^2 \Big ] <\infty . \end{aligned}$$

In particular, $D_{r,\bullet }u(t,x)(\omega ) \in |{\mathcal {P}}_{0}|$ for almost every $(\omega ,r) \in \Omega \times [0,t]$, where $|{\mathcal {P}}_0|$ is defined in (2.2).

#### Proof

We work with a version of $\{ D_{r,z}u(t,x): (r,z)\in [0,t]\times {\mathbb {R}}^2\}$ that is jointly measurable. By Fubini’s theorem and Cauchy-Schwarz inequality, we have

$$\begin{aligned} {\mathbb {E}}\Big [ \big \Vert |D_{r,\bullet } u(t,x)| \big \Vert _{0}^2 \Big ]&\le {\mathbb {E}}\int _{{\mathbb {R}}^{2d}} |D_{r,z}u(t,x)||D_{r,z'}u(t,x)|\gamma (z-z')dzdz' \\&\le \int _{{\mathbb {R}}^{2d}} \Vert D_{r,z}u(t,x)\Vert _2 \Vert D_{r,z'}u(t,x)\Vert _2 \gamma (z-z')dzdz' \\&\!\le \!C \!\!\int _{{\mathbb {R}}^{2d}} G_{t-r}(x\!-\!z)G_{t-r}(x\!-\!z')\gamma (z\!-\!z')dzdz' \,\text {by Theorem}~1.3\\&= C \int _{{\mathbb {R}}^d} \mu (d\xi ) \big \vert {\widehat{G}}_{t-r}(\xi ) \big \vert ^2 \quad \text {using Fourier transform}\\&\le 2C (t^2\vee 1) \int _{{\mathbb {R}}^d}\frac{\mu (d\xi )}{1+|\xi |^2}~\text {by }(2.33), \end{aligned}$$

where *C* is a constant depending on $\gamma _0,\gamma ,t$ and is increasing in *t*. The above (uniform) bound implies (3.29). Hence, $D_{r,\bullet }u(t,x)(\omega ) \in |{\mathcal {P}}_{0}|$ for almost all $(\omega ,r) \in \Omega \times [0,t]$.

$$\square $$

The space $|{\mathcal {H}}\otimes {\mathcal {P}}_0|$ appearing in the next corollary is defined as the set of measurable functions $h:{\mathbb {R}}_+\times {\mathbb {R}}^{2d} \rightarrow {\mathbb {R}}$ such that

$$\begin{aligned}&\int _{{\mathbb {R}}_+^2\times {\mathbb {R}}^{4d}} |h(r, w, z) | | h(r', w', z')|\\&\quad \gamma _0(r-r') \gamma (w-w') \gamma (z-z') dw dw' dz dz' dr dr'<\infty . \end{aligned}$$

Then, $$ |{\mathcal {H}}\otimes {\mathcal {P}}_0| \subset {\mathcal {H}}\otimes {\mathcal {P}}_0$$.

#### Corollary 3.4

Let $d=1,2$. For almost all $(\omega ,r) \in \Omega \times [0,t]$, $D D_{r,\bullet } u(t,x)(\omega ) \in |{\mathcal {H}}\otimes {\mathcal {P}}_0|$ and for any finite $T>0$,

3.30 $$\begin{aligned} \sup _{(t,x) \in [0,T] \times {\mathbb {R}}^d}\sup _{r \in [0,t]} {\mathbb {E}}\left( \Big \Vert \big \vert D D_{r,\bullet }u(t,x) \big \vert \Big \Vert _{{\mathcal {H}}\otimes {\mathcal {P}}_0}^2 \right) < +\infty . \end{aligned}$$

#### Proof

Using Theorem 1.3, Cauchy-Schwarz inequality and the estimate (1.11), we can write

$$\begin{aligned}&{\mathbb {E}}\left( \Big \Vert \big \vert D D_{r,\bullet } u(t,x) \big \vert \Big \Vert _{{\mathcal {H}}\otimes {\mathcal {P}}_0}^2 \right) \\&\quad = {\mathbb {E}}\Bigg (\int _{[0,t]^2} \int _{{\mathbb {R}}^{4d}} |D_{(\theta ,w),(r,z)}^2u(t,x)| |D_{(\theta ',w'),(r,z')}^2u(t,x)|\\&\qquad \times \gamma _0(\theta -\theta ')\gamma (w-w') \gamma (z-z')dw dw'dzdz'd\theta d\theta ' \Bigg ) \\&\quad \le \int _{[0,t]^2} \int _{{\mathbb {R}}^{4d}} \big \Vert D_{(\theta ,w),(r,z)}^2u(t,x) \big \Vert _2 \big \Vert D_{(\theta ',w'),(r,z')}^2u(t,x) \big \Vert _2\\&\qquad \times \gamma _0(\theta -\theta ')\gamma (w-w') \gamma (z-z')dw dw'dzdz'd\theta d\theta ' \\&\quad \le C\int _{[0,t]^2} \int _{{\mathbb {R}}^{4d}} {\widetilde{f}}_{t,x,2}(r,z,\theta , w) {\widetilde{f}}_{t,x,2}(r,z',\theta ', w')\\&\qquad \gamma _0(\theta -\theta ')\gamma (w-w') \gamma (z-z')dw dw'dzdz'd\theta d\theta '. \end{aligned}$$

As a consequence,

$$\begin{aligned}&{\mathbb {E}}\left( \Big \Vert \big \vert D D_{r,\bullet } u(t,x) \big \vert \Big \Vert _{{\mathcal {H}}\otimes {\mathcal {P}}_0}^2 \right) \\&\quad \le C \int _{{\mathbb {R}}^{2d} } \Vert {\widetilde{f}}_{t,x,2}(r,z;\bullet ) \Vert _{{\mathcal {H}}} \Vert {\widetilde{f}}_{t,x,2}(r,z';\bullet ) \Vert _{{\mathcal {H}}}\gamma (z-z') dzdz'. \end{aligned}$$

By the arguments used in the proof of Theorem 1.3, it follows that

$$\begin{aligned} \Vert {\widetilde{f}}_{t,x,2}(r,z;\bullet ) \Vert _{{\mathcal {H}}} \le C G_{t-r}(x-z). \end{aligned}$$

Therefore,

$$\begin{aligned} {\mathbb {E}}\left( \Big \Vert \big \vert D D_{r,\bullet } u(t,x) \big \vert \Big \Vert _{{\mathcal {H}}\otimes {\mathcal {P}}_0}^2 \right) \le C \int _{{\mathbb {R}}^{2d}} \gamma (z-z') G_{t-r}(x-z)G_{t-r}(x-z')dzdz' \end{aligned}$$

and the same argument as in the proof of Corollary 3.3 ends our proof. $\square$

#### Remark 3.5

Note that for any finite $T>0$, ${\mathbb {E}}\big (\big \Vert \vert D^2 u(t,x)\vert \big \Vert _{{\mathcal {H}}^{\otimes 2}}^2\big ) < \infty$ for any $(t,x) \in [0,T] \times {\mathbb {R}}^d$.

## Gaussian fluctuation: Proof of Theorem 1.4

Recall that

$$\begin{aligned} F_R(t)= \int _{B_R} \big [ u(t,x) -1 \big ] dx \end{aligned}$$

and $\sigma _R(t) = \sqrt{\text {Var}\big ( F_R(t) \big ) }$. First, we need to obtain the limiting covariance structure, which is the content of Proposition 4.1. It will give us the growth order of $\sigma _R(t)$. Then, in Sect. 4.2, we apply the second-order Gaussian Poincaré inequality to establish the quantitative CLT for $F_R(t)/\sigma _R(t)$. Finally, we will prove the functional CLT by showing the convergence of the finite-dimensional distributions and the tightness.

### Limiting covariance

#### Proposition 4.1

Let *u* denote the solution to the hyperbolic Anderson model (1.1) and assume that the non-degeneracy condition (1.17) holds. Then, the following results hold true:

1. Suppose $d \in \{1,2\}$ and $\gamma ({\mathbb {R}}^d) \in (0, \infty )$. Then, for any $t,s\in (0,\infty )$,
  4.1 $$\begin{aligned} \lim _{R\rightarrow \infty } R^{-d} {\mathbb {E}}\big [ F_R(t) F_R(s) \big ] = \omega _d \sum _{p\ge 1} p! \int _{{\mathbb {R}}^d} \big \langle {\widetilde{f}}_{t,x,p}, {\widetilde{f}}_{s,0,p} \big \rangle _{{\mathcal {H}}^{\otimes p}}dx, \end{aligned}$$
  see also (1.18). In particular, $\sigma _R(t) \sim R^{d/2}$.
2. Suppose $d \in \{1,2\}$ and $\gamma (x) = |x|^{-\beta }$ for some $\beta \in (0, 2\wedge d)$. Then, for any $t,s\in (0,\infty )$,
  4.2 $$\begin{aligned} \lim _{R\rightarrow \infty } R^{\beta -2d} {\mathbb {E}}\big [ F_R(t) F_R(s) \big ] = \kappa _{\beta , d} \int _0^t dr\int _0^s dr' \gamma _0(r-r') (t-r)(s-r'), \end{aligned}$$
  where $\kappa _{\beta , d} = \int _{B_1^2} dxdy | x- y |^{-\beta }$ is introduced in (1.16). In particular, $\sigma _R(t) \sim R^{d- \frac{\beta }{2}}$.
3. Suppose $d=2$ and $\gamma (x_1,x_2) =\gamma _1(x_1)\gamma _2(x_2)$ satisfies one of the following conditions:
  4.3 $$\begin{aligned} {\left\{ \begin{array}{ll} (c_1) &{} \gamma _i(x_i) = |x_i|^{-\beta _i}~\text {for some }\beta _i\in (0,1), i=1,2; \\ \quad \\ (c_2) &{} \gamma _1\in L^1({\mathbb {R}}) ~\mathrm{and}~ \gamma _2(x) = |x|^{-\beta }~\text {for some }\beta \in (0,1) \end{array}\right. }\,\, . \end{aligned}$$
  For any $s,t\in (0,\infty )$, the following results hold true:
  $(r_1)$ In $(c_1)$, we have
    4.4 $$\begin{aligned}&\lim _{R\rightarrow \infty } R^{\beta _1-\beta _2-4} {\mathbb {E}}\big [ F_R(t) F_R(s) \big ] \nonumber \\&\quad = K_{\beta _1, \beta _2} \int _0^t dr\int _0^s dr' \gamma _0(r-r') (t-r)(s-r'), \end{aligned}$$
    where $K_{\beta _1, \beta _2}$ is defined in (1.22).
  $(r_2)$ In $(c_2)$, we have
    4.5 $$\begin{aligned} \lim _{R\rightarrow \infty } R^{\beta -3} \!{\mathbb {E}}\big [ F_R(t) F_R(s) \big ] \!=\! \gamma _1({\mathbb {R}}) {\mathcal {L}}_\beta \!\! \int _0^t dr\!\int _0^s dr' \gamma _0(r-r') (t-r)(s-r'), \end{aligned}$$
    where ${\mathcal {L}}_\beta$ is defined in (1.24).

#### Proof of part (1) in Proposition 4.1

**Preparation.** In the following, we will denote by $\varphi$ the density of $\mu$. For $0< s\le t < \infty$ and $x,y\in {\mathbb {R}}^d$, we have

$$\begin{aligned} {\mathbb {E}}\big [ u(t,x) u(s,y)\big ]-1&=\sum _{p\ge 1} p! \big \langle {\widetilde{f}}_{t,x,p}, {\widetilde{f}}_{s,y,p}\big \rangle _{{\mathcal {H}}^{\otimes p}} \\&=: \sum _{p\ge 1} \frac{1}{p!} \Phi _p(t,s; x-y), \end{aligned}$$

where ${\widetilde{f}}_{t,x,p}\in {\mathcal {H}}^{\otimes p}$ is defined as in (1.8)–(1.9) and $\Phi _p(t,s; x-y)$, defined in the obvious manner, depends only on the difference $x-y$. To see this dependency and to prepare for the future computations, we rewrite $\Phi _p(t,s; x-y)$ using Fourier transform in space:

4.6 $$\begin{aligned}&\Phi _p(t,s; x-y) = (p!)^2 \big \langle f_{t,x,p}, {\widetilde{f}}_{s,y,p}\big \rangle _{{\mathcal {H}}^{\otimes p}} \nonumber \\&\quad = p! \sum _{\sigma \in {\mathfrak {S}}_p} \int _{\Delta _p(t)} d\pmb {s_p} \int _{[0,s]^p} d\pmb {{\tilde{s}}_p} \left( \prod _{j=1}^p \gamma _0(s_j - {\tilde{s}}_j ) \right) \nonumber \\&\quad \int _{{\mathbb {R}}^{2pd}} d\pmb {y_p} d\pmb {{\tilde{y}}_p} \left( \prod _{j=1}^p\gamma (y_j - {\tilde{y}}_j) \right) \nonumber \\&\qquad \times \left( \prod _{j=0}^{p-1} G_{s_{j} - s_{j+1}}(y_j - y_{j+1}) \right) \left( \prod _{j=0}^{p-1} G_{{\tilde{s}}_{\sigma (j)} - {\tilde{s}}_{\sigma (j+1)}}( {\widetilde{y}}_{\sigma (j)} - {\widetilde{y}}_{\sigma (j+1)}) \right) \end{aligned}$$

4.7 $$\begin{aligned}&= p! \sum _{\sigma \in {\mathfrak {S}}_p} \int _{\Delta _p(t)} d\pmb {s_p} \int _{[0,s]^p} d\pmb {{\tilde{s}}_p} \left( \prod _{j=1}^p \gamma _0(s_j - {\tilde{s}}_{j} ) \right) \nonumber \\&\quad \times \int _{{\mathbb {R}}^{pd}} d \pmb {\xi _p} \left( \prod _{j=1}^p \varphi (\xi _j) \right) e^{-i (x-y)\cdot (\xi _1+\cdots + \xi _p)} \nonumber \\&\, \times \! \left( \! \prod _{j=0}^{p-1} {\widehat{G}}_{s_{j} - s_{j+1}}( \xi _p+\cdots + \xi _{j+1} ) \right) \!\left( \prod _{j=0}^{p-1} {\widehat{G}}_{{\tilde{s}}_{\sigma (j)}\! -\! {\tilde{s}}_{\sigma (j+1)}}(\xi _{\sigma (p)}\!+\! \cdots \!+\! \xi _{\sigma (j+1)}) \right) , \end{aligned}$$

where $\Delta _p(t) =\{ \pmb {s_p}: t> s_1> \cdots> s_p>0\}$, $(s_0, y_0, {\tilde{s}}_{\sigma (0)}, {\tilde{y}}_{\sigma (0)}) = (t,x,s,y)$, ${\widehat{G}}_t(\xi ) = \frac{\sin (t |\xi | )}{| \xi |}$ is introduced in (2.29) and we have used again the convention $G_t(z)=0$ for $t\le 0$.

Relation (4.6) shows that $\Phi _p(t,s; x-y)$ is always nonnegative and equality (4.7) indicates that $\Phi _p(t,s; x-y)$ indeed depends only on the difference $x-y$, so that we can write

4.8 $$\begin{aligned} \Phi _p(t,s; z) = (p!)^2 \big \langle {\widetilde{f}}_{t,z,p}, {\widetilde{f}}_{s,0,p}\big \rangle _{{\mathcal {H}}^{\otimes p}}. \end{aligned}$$

Note that $\Phi _p(t,t; 0)$ coincides with $\alpha _p(t)$ given in [3, Equation (4.11)]. Moreover, applying Lemma 2.5 with $\mu _p(d\pmb {\xi _p}) = \varphi (\xi _1) \cdots \varphi (\xi _p) d\xi _1 \cdots d\xi _p$ and $g(s_1,\xi _1, \dots , s_p,\xi _p) = \prod _{j=0}^{p-1} \vert {\widehat{G}}_{s_{j} - s_{j+1}}( \xi _p+\cdots + \xi _{j+1} ) \vert ,$ we get (with $s\le t$)

4.9 $$\begin{aligned}&\Phi _p(t,s; z) \le \Gamma _t^p p! \int _{\Delta _p(t)} d\pmb {s_p} \int _{{\mathbb {R}}^{pd}} \mu (d\pmb {\xi _p} ) \prod _{j=0}^{p-1} \Big \vert {\widehat{G}}_{s_{j} - s_{j+1}}( \xi _p+\cdots + \xi _{j+1} ) \Big \vert ^2, \end{aligned}$$

where we recall that $\Gamma _t = \int _{-t}^t \gamma _0(a)da$ and point out that the right-hand side of (4.9) is finite by applying Lemma 2.6 with $z_j = \xi _{j+1}+\cdots +\xi _p$ and $z_p=0$.

Now we are ready to show (4.1).

##### Proof of (4.1)

Let us begin with

$$\begin{aligned} \frac{{\mathbb {E}}\big [ F_R(t) F_R(s)\big ]}{R^d}&= \int _{B_R^2} dx dy \frac{{\mathbb {E}}\big [ u(t,x) u(s,y)\big ]-1}{R^d}\\&= \sum _{p\ge 1} \frac{\omega _d }{p!} \int _{{\mathbb {R}}^d} \frac{\text {Leb}\big ( B_R\cap B_R(-z) \big ) }{\text {Leb}( B_R )} \Phi _p(t,s;z)dz, \end{aligned}$$

where $\omega _1=2$, $\omega _2=\pi$ and $\text {Leb}(A)$ stands for the Lebesgue measure of $A\subset {\mathbb {R}}^d$. We claim that

4.10 $$\begin{aligned} \sum _{p\ge 1} \frac{1}{p!} \int _{{\mathbb {R}}^d} \Phi _p(t,s;z)dz < \infty , \end{aligned}$$

from which and the dominated convergence theorem we can deduce that

4.11 $$\begin{aligned} \lim _{R\rightarrow \infty } R^{-d}{\mathbb {E}}\big [ F_R(t) F_R(s)\big ] = \omega _d \sum _{p\ge 1} \frac{1}{p!} \int _{{\mathbb {R}}^d} \Phi _p(t,s;z)dz. \end{aligned}$$

We remark that, by the monotone convergence theorem and the fact that $\Phi _p(t,s;z)\ge 0$ for all $z\in {\mathbb {R}}^d$, the claim (4.10) is equivalent to

4.12 $$\begin{aligned} \sup _{\varepsilon >0} \sum _{p\ge 1} \frac{1}{p!} \int _{{\mathbb {R}}^d} \Phi _p(t,s;z) e^{-\frac{\varepsilon }{2} |z|^2}dz < \infty . \end{aligned}$$

Let us show the claim (4.12).

For $p=1$, by direct computations, we can perform integration with respect to $z, y, {\tilde{y}}$ (one by one in this order) to obtain

4.13 $$\begin{aligned}&\int _{{\mathbb {R}}^d} \Phi _1(t,s;z)dz\nonumber \\&\quad = \int _{{\mathbb {R}}^d} \left( \int _0^t dr \int _0^s d{\tilde{r}} \gamma _0(r-{\tilde{r}}) \int _{{\mathbb {R}}^{2d}} dyd{\tilde{y}} G_{t-r}(y-z)G_{s-{\tilde{r}}}({\tilde{y}}) \gamma (y - {\tilde{y}} ) \right) dz \nonumber \\&\quad =\gamma ({\mathbb {R}}^d) \int _0^t \int _0^s \gamma _0(r-{\tilde{r}}) (t-r) (s-{\tilde{r}} )d{\tilde{r}} dr \le \gamma ({\mathbb {R}}^d) t^3 \Gamma _t, \end{aligned}$$

where $\int _{{\mathbb {R}}^d} \Phi _1(t,s;z)dz >0$ due to the non-degeneracy assumption (1.17) on $\gamma _0$. This implies in particular that $\sigma _R(t) > 0$ for large enough *R*.

Next we consider $p\ge 2$. Using the expression (4.7) and applying Fubini’s theorem with the dominance condition (4.9), we can write

4.14 $$\begin{aligned}&{\mathcal {T}}_{p,\varepsilon }:=(2\pi )^{-d} \int _{{\mathbb {R}}^d} \Phi _p(t,s;z) e^{-\frac{\varepsilon }{2} |z|^2}dz \nonumber \\&\quad = p! \sum _{\sigma \in {\mathfrak {S}}_p} \int _{\Delta _p(t)} d\pmb {s_p} \int _{[0,s]^p} d\pmb {{\tilde{s}}_p} \prod _{j=1}^p \gamma _0(s_j - {\tilde{s}}_{j} ) \int _{{\mathbb {R}}^{pd}} d\pmb {\xi _p} \nonumber \\&\qquad \times p_\varepsilon (\xi _1+\cdots + \xi _p) \prod _{j=0}^{p-1} \varphi (\xi _{j+1}) {\widehat{G}}_{s_{j} - s_{j+1}}( \xi _p+\cdots + \xi _{j+1} ) \nonumber \\&{\widehat{G}}_{{\tilde{s}}_{\sigma (j)} - {\tilde{s}}_{\sigma (j+1)}}(\xi _{\sigma (p)}+ \cdots + \xi _{\sigma (j+1)}) \nonumber \\&\quad \le \Gamma _t^p p! \int _{\Delta _p(t)} d\pmb {s_p} \int _{{\mathbb {R}}^{pd}} d\pmb {\xi _p} \left( \prod _{j=1}^p\varphi (\xi _j) \right) p_\varepsilon \left( \sum _{j=1}^p\xi _j\right) \nonumber \\&\quad \prod _{j=0}^{p-1} \Big \vert {\widehat{G}}_{s_{j} - s_{j+1}}( \xi _p+\cdots + \xi _{j+1} ) \Big \vert ^2, \end{aligned}$$

where $p_\varepsilon (\xi ) =(2\pi \varepsilon )^{-d/2} e^{-|\xi |^2/(2\varepsilon )}$ for $\xi \in {\mathbb {R}}^d$ and we applied Lemma 2.5 with $\mu _p(d\pmb {\xi _p}) =\varphi (\xi _1)\cdots \varphi (\xi _p) p_\varepsilon (\xi _1+\cdots +\xi _p) d\xi _1 \cdots d\xi _p$.

Next, we make the change of variables

$$\begin{aligned} \eta _j= \xi _p +\cdots + \xi _j~\text {with the convention} \eta _{p+1}=0, \end{aligned}$$

and the bound (4.14) becomes

4.15 $$\begin{aligned} {\mathcal {T}}_{p,\varepsilon }&\le \! \Gamma _t^p p! \int _{\Delta _p(t)} d\pmb {s_p} \!\int _{{\mathbb {R}}^{pd}} d\pmb {\eta _p} \left( \prod _{j=1}^p\varphi (\eta _j -\eta _{j+1}) \right) \! p_\varepsilon (\eta _1) \prod _{j=0}^{p-1} \Big \vert {\widehat{G}}_{s_{j} - s_{j+1}}( \eta _{j+1} ) \Big \vert ^2 \nonumber \\&\le \Gamma _t^p p! \Vert \varphi \Vert _\infty t^2 \int _{{\mathbb {R}}^d} d\eta _1 p_\varepsilon (\eta _1) \int _{\Delta _p(t)} d\pmb {s_p} \int _{{\mathbb {R}}^{pd-d}} d\eta _2 \cdots d\eta _p \nonumber \\&\quad \left( \prod _{j=2}^p\varphi (\eta _j -\eta _{j+1}) \right) \Big \vert {\widehat{G}}_{s_{1} - s_2}( \eta _{2} ){\widehat{G}}_{s_{2} - s_3}( \eta _{3} )\cdots {\widehat{G}}_{s_{p-1} - s_p}( \eta _{p} ) \Big \vert ^2\nonumber \\&\quad = \Gamma _t^p p! \Vert \varphi \Vert _\infty t^2 \int _{{\mathbb {R}}^d} d\eta _1 p_\varepsilon (\eta _1) Q_{p-1}, \end{aligned}$$

where we used $| {\widehat{G}}_{t-s_1} (\xi ) | \le t$, and $\varphi (\eta _1-\eta _2)\le \Vert \varphi \Vert _\infty$ (which is finite because $\gamma ({\mathbb {R}}^d)<\infty$) to obtain (4.15), and

4.16 $$\begin{aligned} Q_{p-1}:= \int _{\Delta _{p}(t)} d\pmb {s_{p}} \int _{{\mathbb {R}}^{pd-d}} \prod _{j=2}^p\varphi (\eta _j -\eta _{j+1}) \big \vert {\widehat{G}}_{s_{j-1} -s_j }( \eta _{j} ) \big \vert ^2 d\eta _j. \end{aligned}$$

Observe that $Q_{p-1}$ does not depend on $\eta _1$, thus for any $p\ge 2$

4.17 $$\begin{aligned} {\mathcal {T}}_{p,\varepsilon } \le \Gamma _t^p p! \Vert \varphi \Vert _\infty t^2 Q_{p-1}. \end{aligned}$$

By Lemma 2.6, we have for any $p\ge 2$

$$\begin{aligned} Q_{p-1} \le \left( 2(t^2\vee 1) \int _{{\mathbb {R}}^d} \frac{\mu (d\xi )}{1+ |\xi |^2} \right) ^{p-1} \frac{t^p}{p!} \le \frac{C^p}{p!}. \end{aligned}$$

Now, plugging the above estimate and (4.17) into (4.12), and using (4.13) for $p=1$, we have

$$\begin{aligned} \sup _{\varepsilon >0} \!\sum _{p\ge 1} \frac{1}{p!} \!\int _{{\mathbb {R}}^d} \!\Phi _p(t,s;z) e^{-\frac{\varepsilon }{2} |z|^2}dz \le \gamma ({\mathbb {R}}^d) t^3 \!\Gamma _t+(2\pi )^d \Vert \varphi \Vert _\infty t^2 \!\sum _{p\ge 2} \frac{\Gamma _t^p C^{p}}{p!} \!< \!+\infty . \end{aligned}$$

This shows the claim (4.12) and the claim (4.10), which confirm the limiting covariance structure (4.11). Hence the proof of (4.1) is completed.

$$\square $$

#### Proof of part (2) in Proposition 4.1

In this case, the corresponding spectral density is given by $\varphi (\xi ) = c_{d,\beta } | \xi |^{\beta - d}$, for some constant $c_{d,\beta }$ that only depends on *d* and $\beta$.

Now, let us recall the chaos expansion (1.7) of *u*(*t*, *x*), from which we can obtain the following chaos expansion of $F_R(t)$:

$$\begin{aligned} F_R(t) = \sum _{p\ge 1} {\mathbf {J}}_{p,R}(t), \end{aligned}$$

where ${\mathbf {J}}_{p,R}(t):= I_p\left( \int _{|x| \le R} {\widetilde{f}}_{t,x,p} dx \right)$ is the projection of $F_R(t)$ onto the *p*th Wiener chaos, with ${\widetilde{f}}_{t,x,p}$ given as in (1.9).

Using the orthogonality of Wiener chaoses with different order, we have

$$\begin{aligned} \sigma ^2_R(t)= \text {Var}\big ( F_R(t) \big ) = \sum _{p\ge 1} \text {Var}\big ( {\mathbf {J}}_{p,R}(t) \big ). \end{aligned}$$

Let us first consider the variance of ${\mathbf {J}}_{1,R}(t)$. With $B_R=\{ x\in {\mathbb {R}}^d: |x | \le R\}$, we can write

4.18 $$\begin{aligned}&\text {Var}\big ( {\mathbf {J}}_{1,R}(t) \big ) = \int _{B_R^2} dxdx' \langle G_{t-\bullet }(x-*), G_{t-\bullet }(x'-*) \rangle _{{\mathcal {H}}}\nonumber \\&\quad = \int _{B_R^2} dxdx' \int _{[0,t]^2} dsds' \gamma _0(s-s') \int _{{\mathbb {R}}^d} d\xi \varphi (\xi ) e^{-i (x-x') \cdot \xi } {\widehat{G}}_{t-s}(\xi ) {\widehat{G}}_{t-s'}(\xi ). \end{aligned}$$

Then, making the change of variables $(x, x', \xi )\rightarrow (Rx, Rx', \xi /R)$, we get

$$\begin{aligned}&\text {Var}\big ( {\mathbf {J}}_{1,R}(t) \big ) =R^{2d-\beta } \int _{[0,t]^2} dsds' \gamma _0(s-s') \int _{B_1^2} dxdx' \\&\quad \int _{{\mathbb {R}}^d} d\xi \varphi (\xi ) e^{-i (x-x') \cdot \xi } {\widehat{G}}_{t-s}(\xi /R) {\widehat{G}}_{t-s'}(\xi /R). \end{aligned}$$

Note that ${\widehat{G}}_{t}(\xi /R)$ is uniformly bounded and convergent to *t* as $R\rightarrow \infty$; observe also that

4.19 $$\begin{aligned} \ell _{R}(\xi ):=\int _{B_R^2} dxdx' e^{-i (x-x') \cdot \xi } = \big \vert {\mathcal {F}}{\mathbf {1}}_{B_R}\big \vert ^2(\xi ) \in [0,\infty ). \end{aligned}$$

Thus we deduce from the dominated convergence theorem that, with $\kappa _{\beta ,d} :=\int _{B_1^2} dxdx' | x- x' |^{-\beta }$,

4.20 $$\begin{aligned}&\frac{\text {Var}\big ( {\mathbf {J}}_{1,R}(t) \big )}{R^{2d-\beta }} \xrightarrow {R\rightarrow \infty } \int _{[0,t]^2} dsds' \gamma _0(s-s') (t-s) (t-s') \int _{{\mathbb {R}}^d} d\xi \varphi (\xi ) \big \vert {\mathcal {F}}{\mathbf {1}}_{B_1}\big \vert ^2(\xi ) \nonumber \\&\quad = \kappa _{\beta ,d} \int _{[0,t]^2} dsds' \gamma _0(s-s') s s' . \end{aligned}$$

In the same way, we can get

4.21 $$\begin{aligned} \frac{ {\mathbb {E}}\big [ {\mathbf {J}}_{1,R}(t) {\mathbf {J}}_{1,R}(s) \big ]}{R^{2d-\beta }} \xrightarrow {R\rightarrow \infty }&\kappa _{\beta ,d}\int _{0}^t dr \int _0^s dr' \gamma _0(r-r') (t-r)(s-r') \end{aligned}$$

In what follows, we will show that as $R\rightarrow \infty$,

4.22 $$\begin{aligned} \sum _{p\ge 2} \text {Var}\big ( {\mathbf {J}}_{p,R}(t) \big ) = o(R^{2d-\beta }). \end{aligned}$$

In view of the orthogonality again, the above claim (4.22) and the results (4.20)–(4.21) imply that the first chaos of $F_R(t)$ is dominant and

$$\begin{aligned} \frac{ {\mathbb {E}}\big [ F_R(t) F_{R}(s) \big ] }{R^{2d-\beta }} \xrightarrow {R\rightarrow \infty } \kappa _{\beta ,d}\int _{0}^t dr \int _0^s dr' \gamma _0(r-r') (t-r)(s-r'), \end{aligned}$$

which gives us the desired limiting covariance structure. Moreover, we obtain immediately that the process $\big \{R^{-d+\frac{\beta }{2} } F_R(t): t\in {\mathbb {R}}_+\big \}$ converges in finite-dimensional distributions to the centered Gaussian process ${\mathcal {G}}_\beta$, whose covariance structure is given by (1.19).

The rest of Sect. 4.1.2 is then devoted to proving (4.22). We point out that the strategy in Sect. 4.1.1 can not be directly used, because $\varphi$ is not uniformly bounded here.

##### Proof of Claim (4.22)

We begin by writing (with $s_0 ={\tilde{s}}_{\sigma (0)} = t$ and $B_R=\{ x: |x| \le R\}$)

$$\begin{aligned}&\text {Var}\big ( {\mathbf {J}}_{p,R}(t) \big ) = p! \int _{B_R^2}dxdx' \big \langle {\widetilde{f}}_{t,x,p}, {\widetilde{f}}_{t,x',p} \big \rangle _{{\mathcal {H}}^{\otimes p}}=p! \int _{B_R^2}dxdx' \big \langle f_{t,x,p}, {\widetilde{f}}_{t,x',p} \big \rangle _{{\mathcal {H}}^{\otimes p}} \\&\quad = c_{d,\beta }^p \sum _{\sigma \in {\mathfrak {S}}_p} \int _{B_R^2}dxdx' \int _{[0,t]^{2p}} d\pmb {s_p}d\pmb {{\tilde{s}}_p} \prod _{k=1}^p \gamma _0(s_k- {\tilde{s}}_{k} ) \int _{{\mathbb {R}}^{pd}} \left( \prod _{j=1}^p d\xi _j | \xi _j|^{\beta -d} \right) \\&\qquad \times e^{-i (x-x')\cdot (\xi _p+\cdots +\xi _1)} \prod _{j=0}^{p-1} {\widehat{G}}_{s_j - s_{j+1}}(\xi _p + \cdots + \xi _{j+1} ) {\widehat{G}}_{{\tilde{s}}_{\sigma (j)} - {\tilde{s}}_{\sigma (j+1)}}\\&\qquad \times (\xi _{\sigma (p)} + \cdots + \xi _{\sigma (j+1)} ), \end{aligned}$$

where we recall the convention that $G_t(z)=0$ for $t\le 0$.

Then, recalling definition (4.19) of $\ell _{R}(\xi )$, we can apply Lemma 2.5 with

$$\begin{aligned} \mu (d\pmb {\xi _p} ) = \varphi (\xi _1) \cdots \varphi (\xi _p) \ell _R(\xi _1 + \cdots + \xi _p) d\xi _1 \cdots d\xi _p \end{aligned}$$

to get $\text {Var}\big ( {\mathbf {J}}_{p,R}(t) \big )$ bounded by

4.23 $$\begin{aligned}&c_{d,\beta }^p \Gamma _t^p \int _{\Delta _p(t)} d\pmb {s_p}\int _{{\mathbb {R}}^{pd}} \left( \prod _{j=1}^p d\xi _j | \xi _j|^{\beta -d} \right) \ell _R(\xi _1+\cdots +\xi _p)\nonumber \\&\quad \times \prod _{j=0}^{p-1} \Big \vert {\widehat{G}}_{s_j - s_{j+1}}(\xi _p + \cdots + \xi _{j+1} ) \Big \vert ^2. \end{aligned}$$

Making change of variables

$$\begin{aligned} \mathrm{(i)} ~\eta _j = \xi _p+\cdots + \xi _j~\text {with }\eta _{p+1}=0 \quad (ii)~ (x, x', \eta _1)\rightarrow (Rx, Rx', \eta _1 R^{-1}), \end{aligned}$$

we obtain

$$\begin{aligned} \text {Var}\big ( {\mathbf {J}}_{p,R}(t) \big )&\le c_{d,\beta }^p \Gamma _t^p \int _{\Delta _p(t)} d\pmb {s_p}\int _{{\mathbb {R}}^{pd}} \left( \prod _{j=1}^p d\eta _j | \eta _j - \eta _{j+1}|^{\beta -d} \right) \\&\quad \times \left( \int _{B_R^2}dxdx' e^{-i (x-x')\cdot \eta _1}\right) \prod _{j=0}^{p-1} \Big \vert {\widehat{G}}_{s_j - s_{j+1}}(\eta _{j+1} ) \Big \vert ^2\\&= c_{d,\beta }^p \Gamma _t^p R^{2d-\beta } \int _{\Delta _p(t)} d\pmb {s_p}\int _{{\mathbb {R}}^{pd}} d\eta _1 | \eta _1 - \eta _2 R|^{\beta -d}\\&\quad \left( \prod _{j=2}^p d\eta _j | \eta _j - \eta _{j+1}|^{\beta -d} \right) \\&\quad \times \left( \int _{B_1^2}dxdx' e^{-i (x-x')\cdot \eta _1}\right) \Big \vert {\widehat{G}}_{t-s_1}( \eta _1/R ) \Big \vert ^2 \prod _{j=1}^{p-1} \Big \vert {\widehat{G}}_{s_j - s_{j+1}}(\eta _{j+1} ) \Big \vert ^2 \\&\le t^2 c_{d,\beta }^{p-1} \Gamma _t^p R^{2d-\beta } \int _{\Delta _p(t)} d\pmb {s_p}\int _{{\mathbb {R}}^{pd-d}} \left( \prod _{j=2}^p d\eta _j | \eta _j - \eta _{j+1}|^{\beta -d} \right) \\&\quad \times \left( \int _{B_1^2}dxdx' |x-x'|^{-\beta } e^{-i (x-x')\cdot \eta _2 R}\right) \prod _{j=1}^{p-1} \Big \vert {\widehat{G}}_{s_j - s_{j+1}}(\eta _{j+1} ) \Big \vert ^2, \end{aligned}$$

where in the last inequality we used $| {\widehat{G}}_t | \le t$ and the following Fourier transform:

$$\begin{aligned}&\int _{B_1^2}dxdx' c_{d,\beta } \int _{{\mathbb {R}}^d} d\eta _1 | \eta _1 - \eta _2 R |^{\beta -d} e^{-i (x-x')\cdot \eta _1}\\&=c_{d,\beta } \int _{{\mathbb {R}}^d} d\eta _1 | \eta _1 - \eta _2 R |^{\beta -d} \big \vert {\mathcal {F}}{\mathbf {1}}_{B_1} \big \vert ^2(\eta _1) \\&=\int _{B_1^2}dxdx' | x- x' |^{-\beta } e^{-i (x-x')\cdot \eta _2 R}. \end{aligned}$$

Note that the integral $\int _{B_1^2}dxdx' |x-x'|^{-\beta } e^{-i (x-x')\cdot \eta _2 R}$ is uniformly bounded by $\kappa _{\beta ,d}$ and it converges to zero as $R\rightarrow \infty$ for $\eta _2\ne 0$. This convergence is a consequence of the Riemann-Lebesgue’s lemma. Taking into account the definition (4.16) of $Q_{p-1}$, then we have

$$\begin{aligned} R^{\beta -2d} \text {Var}\big ( {\mathbf {J}}_{p,R}(t) \big ) \le t^2 \kappa _{\beta ,d} \Gamma _t^p Q_{p-1}, \end{aligned}$$

which is summable over $p\ge 2$ by the arguments in the previous section. Hence by the dominated convergence theorem, we get

$$\begin{aligned} R^{\beta -2d} \sum _{p\ge 2} \text {Var}\big ( {\mathbf {J}}_{p,R}(t) \big ) \xrightarrow {R\rightarrow \infty } 0. \end{aligned}$$

This proves the claim (4.22). $\square$

#### Proof of part (3) in Proposition 4.1

Recall the two cases from (4.3):

$$\begin{aligned} {\left\{ \begin{array}{ll} (c_1) &{} \gamma _i(x_i) = |x_i|^{-\beta _i}~\text {for some }\beta _i\in (0,1), i=1,2, \\ (c_2) &{} \gamma _1\in L^1({\mathbb {R}}) ~\mathrm{and}~ \gamma _2(x) = |x|^{-\beta }~\text {for some }\beta \in (0,1). \end{array}\right. }. \end{aligned}$$

In $(c_1)$, the spectral density is $\varphi (\xi _1, \xi _2) =c_{1,\beta _1}c_{1,\beta _2} | \xi _1|^{\beta _1-1} | \xi _2|^{\beta _2-1}$ for $(\xi _1,\xi _2)\in {\mathbb {R}}^2$, where $c_{1,\beta }$ is a constant that only depends on $\beta$. Now, using the notation from Sect. 4.1.2, we write

$$\begin{aligned}&\text {Var}\big ( {\mathbf {J}}_{1,R}(t) \big ) = \int _{B_R^2} dxdx' \int _{[0,t]^2} dsds' \gamma _0(s-s') \\&\qquad \times \int _{{\mathbb {R}}^d} d\xi \varphi (\xi ) e^{-i (x-x') \cdot \xi } {\widehat{G}}_{t-s}(\xi ) {\widehat{G}}_{t-s'}(\xi ) \quad \text {see }(4.18) \\&\quad =R^{4-\beta _1 -\beta _2} \int _{[0,t]^2} dsds' \gamma _0(s-s') \int _{{\mathbb {R}}^d} d\xi \varphi (\xi _1, \xi _2) \\&\qquad \times \int _{B_1^2} dxdx' e^{-i (x-x') \cdot \xi } {\widehat{G}}_{t-s}(\xi /R) {\widehat{G}}_{t-s'}(\xi /R), \end{aligned}$$

where the last equality is obtained by the change of variables $(x,x', \xi _1, \xi _2)$ to $(Rx,Rx', \xi _1/R, \xi _2/R)$. Thus, by the exactly same arguments that lead to (4.20), we can get

$$\begin{aligned} \frac{\text {Var}\big ( {\mathbf {J}}_{1,R}(t) \big )}{R^{4-\beta _1 - \beta _2}} \xrightarrow {R\rightarrow \infty } K_{\beta _1, \beta _2}\int _{[0,t]^2} dsds' \gamma _0(s-s') ss', \end{aligned}$$

with $K_{\beta _1, \beta _2}$ introduced in (1.22). Similar to (4.21), we also have

4.24 $$\begin{aligned} \frac{{\mathbb {E}}\big [ {\mathbf {J}}_{1,R}(t) {\mathbf {J}}_{1,R}(s) \big ]}{R^{4-\beta _1 - \beta _2}} \xrightarrow {R\rightarrow \infty } K_{\beta _1, \beta _2}\int _{0}^t dr \int _0^s dr' \gamma _0(r-r') (t-r)(s-r'). \end{aligned}$$

To obtain the result $(r_1)$, it remains to show

4.25 $$\begin{aligned} \sum _{p\ge 2} \text {Var}\big ( {\mathbf {J}}_{p,R}(t) \big ) = o\big ( R^{4-\beta _1 - \beta _2} \big ). \end{aligned}$$

Its proof can be done *verbatim* as for the result (4.22), so we omit the details here.

Finally, let us look at the more interesting case $(c_2)$ where $\gamma _1\in L^1({\mathbb {R}})$ and $\gamma _2(x) =|x|^{-\beta }$ for some fixed $\beta \in (0,1)$. In this case, the corresponding spectral density is $\varphi (\xi _1,\xi _2) = \varphi _1(\xi _1) \varphi _2(\xi _2)$, where

4.26 $$\begin{aligned} {\left\{ \begin{array}{ll} \mathrm{(i)} &{} \gamma _1 = {\mathcal {F}}\varphi _1\text { and }\varphi _1\text { is uniformly continuous and bounded, } \\ \mathrm{(ii)} &{} \varphi _2(\xi _2) =c_{1,\beta } |\xi _2|^{\beta -1} \text { for some constant }c_{1,\beta }\text { that only depends on }\beta . \end{array}\right. } \end{aligned}$$

Let us begin with (4.18) and make the usual change of variables $(x,x', \xi )\rightarrow (Rx,Rx', \xi /R)$ to obtain

$$\begin{aligned}&\text {Var}\big ( {\mathbf {J}}_{1,R}(t) \big ) = \int _{B_R^2} dxdx' \int _{[0,t]^2} dsds' \gamma _0(s-s') \\&\quad \int _{{\mathbb {R}}^2} d\xi \varphi _1(\xi _1)\varphi _2(\xi _2) e^{-i (x-x') \cdot \xi } {\widehat{G}}_{t-s}(\xi ) {\widehat{G}}_{t-s'}(\xi ) \\&\quad =R^{3-\beta } \int _{[0,t]^2} dsds' \gamma _0(s-s') \int _{{\mathbb {R}}^2} d\xi \varphi _1(\xi _1/R)\varphi _2(\xi _2) \\&\quad \left( \int _{B_1^2} dxdx' e^{-i (x-x') \cdot \xi } \right) {\widehat{G}}_{t-s}(\xi /R) {\widehat{G}}_{t-s'}(\xi /R)\\&\quad =R^{3-\beta } \int _{[0,t]^2} dsds' \gamma _0(s-s') \\&\quad \int _{{\mathbb {R}}^2} d\xi \varphi _1(\xi _1/R)\varphi _2(\xi _2) \big \vert {\mathcal {F}}{\mathbf {1}}_{B_1} \big \vert ^2(\xi ) {\widehat{G}}_{t-s}(\xi /R) {\widehat{G}}_{t-s'}(\xi /R). \end{aligned}$$

Recall that $\varphi _1$, ${\widehat{G}}_{t-s}$ and ${\widehat{G}}_{t-s'}$ are uniformly bounded and continuous. Note that, applying Plancherel’s theorem and the Parseval-type relation (2.3), we have

$$\begin{aligned} \int _{{\mathbb {R}}^2} d\xi \varphi _2(\xi _2) \big \vert {\mathcal {F}}{\mathbf {1}}_{B_1} \big \vert ^2(\xi )&= 2\pi \int _{{\mathbb {R}}^2} dx_1 d\xi _2 \varphi _2(\xi _2) \left| {\mathcal {F}}{\mathbf {1}}_{B_1} (x_1, \bullet )(\xi _2) \right| ^2 \\&= 2\pi \!\int _{{\mathbb {R}}^3} dx_1dx_2 dx_3 {\mathbf {1}}_{\{ x_1^2 \!+\! x_2^2\!\le \! 1 \}} {\mathbf {1}}_{\{ x_1^2 + x_3^2\le 1 \}} |x_2\!-\!x_3|^{-\beta }\!<\!\infty . \end{aligned}$$

Therefore, by the dominated convergence theorem and the fact that $\varphi _1(0) = \frac{1}{2\pi }\gamma _1({\mathbb {R}})$, we get

$$\begin{aligned}&\frac{ \text {Var}\big ( {\mathbf {J}}_{1,R}(t) \big ) }{R^{3-\beta }}\xrightarrow {R\rightarrow \infty } \varphi _1(0) \int _{[0,t]^2} dsds' \gamma _0(s-s') (t-s) (t-s')\\&\qquad \qquad \qquad \times \int _{{\mathbb {R}}^2} d\xi \varphi _2(\xi _2) \big \vert {\mathcal {F}}{\mathbf {1}}_{B_1} \big \vert ^2(\xi ) \\&\qquad \qquad \qquad = \gamma _1({\mathbb {R}}) {\mathcal {L}}_\beta \int _{[0,t]^2} dsds' \gamma _0(s-s')ss', \end{aligned}$$

where ${\mathcal {L}}_\beta$ is defined in (1.24). In the same way, we get for $s,t\in (0,\infty )$,

4.27 $$\begin{aligned} \frac{ {\mathbb {E}}\big [ {\mathbf {J}}_{1,R}(t) {\mathbf {J}}_{1,R}(s) \big ] }{R^{3-\beta }}\xrightarrow {R\rightarrow \infty } \gamma _1({\mathbb {R}}) {\mathcal {L}}_\beta \int _0^t dr \int _0^s dr' \gamma _0(r-r') (t-r)(s-r'). \end{aligned}$$

Now we claim that the other chaoses are negligible, that is, as $R\rightarrow \infty$,

4.28 $$\begin{aligned} \sum _{p\ge 2} \text {Var}\big ( {\mathbf {J}}_{p,R}(t) \big ) = o(R^{3-\beta }). \end{aligned}$$

Note that the desired limiting covariance structure follows from (4.27) and the above claim (4.28). The rest of this section is devoted to proving claim (4.28).

##### Proof of Claim (4.28)

By the same arguments that lead to the estimate (4.23), we can obtain

$$\begin{aligned}&\text {Var}\big ( {\mathbf {J}}_{p,R}(t) )\le \Gamma _t^p \int _{\Delta _p(t)} d\pmb {s_p}\int _{{\mathbb {R}}^{2p}} d\pmb {\xi _p} \varphi _p(\pmb {\xi _p}) \\&\quad \quad \times \prod _{j=0}^{p-1} \Big \vert {\widehat{G}}_{s_j - s_{j+1}}(\xi _p + \cdots + \xi _{j+1} ) \Big \vert ^2 ~\text {with }s_0=t, \end{aligned}$$

where $\varphi _p(\pmb {\xi _p}) = \varphi (\xi _1)\cdots \varphi (\xi _p) \ell _R(\xi _1+ \cdots + \xi _p)$ for $\xi _j = (\xi _{j}^{(1)}, \xi _{j}^{(2)})\in {\mathbb {R}}^2$, $j=1,\dots ,p$ and $\ell _R$ is defined in (4.19). Recall that in the current case, $\varphi (\xi ) = \varphi _1(\xi ^{(1)}) \varphi _2( \xi ^{(2)} )$ for $\xi =(\xi ^{(1)},\xi ^{(2)} )\in {\mathbb {R}}^2$ and $\varphi _1, \varphi _2$ satisfy the conditions in (4.26). Then, the following change of variables

$$\begin{aligned} \eta _j = \xi _j + \xi _{j+1} + \cdots + \xi _p\text { with }\eta _{p+1}=0 \end{aligned}$$

yields

$$\begin{aligned} \text {Var}\big ( {\mathbf {J}}_{p,R}(t) ) \!&\le \! \Gamma _t^p \int _{\Delta _p(t)} d\pmb {s_p}\int _{{\mathbb {R}}^{2p}} d\pmb {\eta _p} \!\ell _R(\eta _1) \!\prod _{j=0}^{p-1} \varphi (\eta _{j+1} \!-\! \eta _{j+2} ) \Big \vert {\widehat{G}}_{s_j - s_{j+1}}(\eta _{j+1} ) \Big \vert ^2. \end{aligned}$$

In view of (4.19), we have $\ell _R(\eta _1/R) = R^4 \ell _1(\eta _1)$. Thus, by changing $\eta _1$ to $\eta _1/R$, we write

$$\begin{aligned} \text {Var}\big ( {\mathbf {J}}_{p,R}(t) )&\le R^2 \Gamma _t^p \int _{\Delta _p(t)} d\pmb {s_p}\int _{{\mathbb {R}}^{2p}} d\pmb {\eta _p} \ell _1(\eta _1) \varphi (\eta _1R^{-1} - \eta _2) \Big \vert {\widehat{G}}_{t - s_{1}}(\eta _{1}/R ) \Big \vert ^2\\&\quad \times \prod _{j=1}^{p-1} \varphi (\eta _{j+1} - \eta _{j+2} ) \Big \vert {\widehat{G}}_{s_j - s_{j+1}}(\eta _{j+1} ) \Big \vert ^2 \\&\le R^{3-\beta } \Gamma _t^p \Vert \varphi _1 \Vert _\infty t^2 \int _{\Delta _p(t)} d\pmb {s_p}\int _{{\mathbb {R}}^{2p-2}} d\eta _2 ... d\eta _p\\&\quad \left( \int _{{\mathbb {R}}^2} d\eta _1 \ell _1(\eta _1) c_{1,\beta } \big \vert \eta ^{(2)}_1 - \eta _2^{(2)}R \big \vert ^{\beta -1} \right) \\&\quad \times \prod _{j=1}^{p-1} \varphi (\eta _{j+1} - \eta _{j+2} ) \Big \vert {\widehat{G}}_{s_j - s_{j+1}}(\eta _{j+1} ) \Big \vert ^2, \end{aligned}$$

where we used $\vert {\widehat{G}}_{t - s_{1}}(\eta _{1}/R ) \vert ^2 \le t^2$. Observe that with $\eta = (\eta ^{(1)}, \eta ^{(2)})$, we deduce from the fact $\ell _1(\eta ) = \big \vert {\mathcal {F}}{\mathbf {1}}_{B_1}\big \vert ^2(\eta ^{(1)}, \eta ^{(2)})$ that

$$\begin{aligned} \int _{{\mathbb {R}}^2} d\eta \ell _1(\eta ) \varphi _2(\eta ^{(2)} - x R)&= \int _{{\mathbb {R}}^2} d\eta ^{(1)}d \eta ^{(2)} \big \vert {\mathcal {F}}{\mathbf {1}}_{B_1}\big \vert ^2(\eta ^{(1)}, \eta ^{(2)}+xR) \varphi _2(\eta ^{(2)} ) \\&= 2\pi \int _{{\mathbb {R}}^3} {\mathbf {1}}_{\{ x_1^2+x_2^2\le 1 \}}{\mathbf {1}}_{\{ x_1^2+x_3^2\le 1 \}}\\&\quad e^{-i(x_2-x_3) xR} |x_2- x_3|^{-\beta }dx_1dx_2dx_3, \end{aligned}$$

by inverting the Fourier transform. The above quantity is uniformly bounded by $2\pi {\mathcal {L}}_\beta$ with ${\mathcal {L}}_\beta$ given in (1.24) and convergent to zero as $R\rightarrow \infty$ for every $x\ne 0$ in view of the Riemann-Lebesgue lemma. Thus, $R^{\beta -3} \text {Var}\big ( {\mathbf {J}}_{p,R}(t) )$ is uniformly bounded by $2\pi {\mathcal {L}}_\beta \Gamma _t^p \Vert \varphi _1 \Vert _\infty t^2 Q_{p-1}$, with $Q_{p-1}$ given by (4.16) and it converges to zero as $R\rightarrow \infty$. Since $Q_{p} \le C^p/p!$, we have

$$\begin{aligned} \sum _{p\ge 2} \Gamma _t^p Q_{p-1} <\infty , \end{aligned}$$

and the dominated convergence theorem implies (4.28). $\square$

##### Remark 4.2

Under the assumptions of Proposition  4.1, we point out that $\sigma _R(t) > 0$ for large enough *R* so that the renormalized random variable $F_R(t)/ \sigma _R(t)$ is well-defined for large *R*.

### Quantitative central limit theorems (QCLT) and f.d.d. convergence

In this section, we prove the quantitative CLTs that are stated in Theorem 1.4 and, as an easy consequence, we are also able to show the convergence of finite-dimensional distributions in Theorem 1.4. We consider first the part (1) and later we treat parts (2) and (3).

#### Part (1)

We will first show the estimate

4.29 $$\begin{aligned} d_{\mathrm{TV}}\big ( F_R(t)/\sigma _R(t), Z\big ) \lesssim R^{-d/2}, \end{aligned}$$

where $Z \sim N(0,1)$. By Proposition 1.8 applied to $\frac{1}{ \sigma _R(t)} F_R(t)$, we have

4.30 $$\begin{aligned} d_{\mathrm{TV}}\big ( F_R(t)/\sigma _R(t), Z\big ) \le \frac{4}{ \sigma ^2_R(t)} \sqrt{{\mathcal {A}}_R}, \end{aligned}$$

where

$$\begin{aligned} {\mathcal {A}}_R&= \int _{{\mathbb {R}}_+^6\times {\mathbb {R}}^{6d}} drdr' dsds' d\theta d\theta ' dzdz' dydy' dwdw' \\&\quad \gamma _0(\theta - \theta ') \gamma _0(s-s') \gamma _0(r-r')\gamma (z-z') \gamma (w-w') \\&\quad \times \gamma (y-y') \Vert D_{r,z}D_{\theta ,w}F_R(t) \Vert _4 \Vert D_{s,y}D_{\theta ',w'}F_R(t) \Vert _4 \Vert D_{r',z'}F_R(t)\Vert _4 \Vert \\&\quad D_{s', y' }F_R(t) \Vert _4. \end{aligned}$$

Recall from Sect. 4.1.1 that $\sigma ^2_R(t) \sim R^d$. Therefore, in order to show (4.29) it suffices to prove the estimate

4.31 $$\begin{aligned} {\mathcal {A}}_R \lesssim R^d. \end{aligned}$$

Using Minkowski’s inequality, we can write

$$\begin{aligned} \Vert D_{r,z}D_{\theta ,w}F_R(t) \Vert _4 = \left\| \int _{B_R} D_{r,z}D_{\theta ,w} u(t,x) dx \right\| _4 \le \int _{B_R} \big \Vert D_{r,z}D_{\theta ,w} u(t,x) \big \Vert _4 dx. \end{aligned}$$

Then, it follows from our fundamental estimates in Theorem 1.3 that

4.32 $$\begin{aligned} \Vert D_{r,z}D_{\theta ,w}F_R(t) \Vert _4 \lesssim \int _{B_R} {\widetilde{f}}_{t,x,2}(r,z, \theta , w) dx, \end{aligned}$$

with

$$\begin{aligned}&{\widetilde{f}}_{t,x,2}(r,z,\theta ,w) \\&\quad = \frac{1}{2} \left[ G_{t-r}(x-z) G_{r-\theta }(z-w){\mathbf {1}}_{\{ r > \theta \}} + G_{t-\theta }(x-w) G_{\theta -r}(z-w){\mathbf {1}}_{\{ r < \theta \}}\right] ; \end{aligned}$$

and, in the same way, we have

4.33 $$\begin{aligned} \Vert D_{r,z}F_R(t) \Vert _4 \lesssim \int _{B_R} G_{t-r}(x-z)dx, \end{aligned}$$

where the implicit constants in (4.32)–(4.33) do not depend on $(R, r,z,\theta ,w)$ and are increasing in *t*. Now, plugging (4.32)–(4.33) into the expression of ${\mathcal {A}}_R$, we get

$$\begin{aligned}&{\mathcal {A}}_R\lesssim \int _{[0,t]^6\times {\mathbb {R}}^{6d}} drdr'dsds' d\theta d\theta ' dzdz' dydy' dwdw' \\&\qquad \times \gamma _0(r-r') \gamma _0(s-s') \gamma _0(\theta - \theta ')\gamma (z-z') \gamma (w-w') \\&\qquad \times \gamma (y-y') \int _{B_R^4} {\widetilde{f}}_{t,x_1,2}(r,z, \theta , w) {\widetilde{f}}_{t,x_2,2}(s,y, \theta ', w') G_{t-r'}(x_3- z')\\&\qquad \times G_{t-s'}(x_4 - y') d\pmb {x_4} =: \sum _{j=1}^4{\mathcal {A}}_{R,j}. \end{aligned}$$

The four terms ${\mathcal {A}}_{R,1}, \dots , {\mathcal {A}}_{R,4}$ are defined according to whether $r>\theta$ or $r<\theta$, and whether $s>\theta '$ or $s<\theta '$. For example, the term ${\mathcal {A}}_{R,1}$ corresponds to $r>\theta$ and $s>\theta '$:

4.34 $$\begin{aligned} {\mathcal {A}}_{R,1}&= \frac{1}{4} \int _{[0,t]^6\times {\mathbb {R}}^{6d}} drdr'dsds' d\theta d\theta ' dzdz' dydy' dwdw'\nonumber \\&\qquad \times \gamma _0(r-r') \gamma _0(s-s') \gamma _0(\theta - \theta ') \nonumber \\&\qquad \times \gamma (w-w') \gamma (y-y') \gamma (z-z')G_{r-\theta }(z-w)G_{s-\theta '}(y-w') \nonumber \\&\qquad \times \int _{B_R^4} d\pmb {x_4} G_{t-r}(x_1-z) G_{t-s}(x_2-y) G_{t-r'}(x_3- z') G_{t-s'}(x_4 - y'). \end{aligned}$$

The term ${\mathcal {A}}_{R,2}$ corresponds to $r>\theta$ and $s<\theta '$, the term ${\mathcal {A}}_{R,3}$ corresponds to $r<\theta$ and $s>\theta '$ and the term ${\mathcal {A}}_{R,4}$ corresponds to $r< \theta$ and $s<\theta '$. In the following, we estimate ${\mathcal {A}}_{R,j}$ for $j=1,2,3,4$ by a constant times $R^{d}$, which yields (4.31).

To get the bound for ${\mathcal {A}}_{R,1}$, it suffices to perform the integration with respect to $dx_1, dx_2, dx_4$, $dy', dy, dw', dw$, $dz, dz', dx_3$ one by one, by taking into account the following facts:

$$\begin{aligned} \sup _{z\in {\mathbb {R}}^d} \int _{B_R} G_{t-r}(x-z)dx \le t \quad \mathrm{and}\quad \sup _{y'\in {\mathbb {R}}^d}\int _{{\mathbb {R}}^d} \gamma (y-y') dy = \Vert \gamma \Vert _{L^1({\mathbb {R}}^d)}. \end{aligned}$$

To get the bound for ${\mathcal {A}}_{R,2}$, it suffices to perform the integration with respect to $dx_1, dx_3,dz', dz$, $dx_2, dw, dw', dy, dy', dx_4$. To get the bound for ${\mathcal {A}}_{R,3}$, it suffices to perform the integration with respect to $dx_4, dy', dx_2, dy, dw', dx_1, dw, dz, dz', dx_3$ one by one. To get the bound for ${\mathcal {A}}_{R,4}$, it suffices to perform the integration with respect to $dx_1, dx_3, dx_2, dz', dz, dw, dw', dy, dy', dx_4$ one by one. This completes the proof of (4.29).

In the second part of this subsection, we show the f.d.d. convergence in Theorem 1.4-(1).

Fix an integer $m\ge 1$ and choose $t_1, \dots , t_m\in (0,\infty )$. Put ${\mathbf {F}}_R = \big ( F_R(t_1), \dots , F_R(t_m) \big )$. Then, by the result on limiting covariance structure from Sect. 4.1.1, we have that the covariance matrix of $R^{-d/2}{\mathbf {F}}_R$, denoted by ${\mathcal {C}}_R$, converges to the matrix ${\mathcal {C}} = ({\mathcal {C}}_{ij}: 1\le i,j \le m)$, with

$$\begin{aligned} {\mathcal {C}}_{ij}= \omega _d \sum _{p\ge 1} p! \int _{{\mathbb {R}}^d} \big \langle {\widetilde{f}}_{t_i,x,p}, {\widetilde{f}}_{t_j,0,p} \big \rangle _{{\mathcal {H}}^{\otimes p}}dx. \end{aligned}$$

Since $F_R(t)=\delta (-DL^{-1}F_R(t))$, according to [25, Theorem 6.1.2],^11^ for any twice differentiable function $h: {\mathbb {R}}^m \rightarrow {\mathbb {R}}$ with bounded second partial derivatives,

4.35 $$\begin{aligned}&\Big \vert {\mathbb {E}}\big [ h(R^{-d/2}{\mathbf {F}}_R) - h({\mathbf {Z}}) \big ] \Big \vert \le \Big \vert {\mathbb {E}}\big [ h(R^{-d/2}{\mathbf {F}}_R) - h({\mathbf {Z}}_R) \big ] \Big \vert + \Big \vert {\mathbb {E}}\big [ h({\mathbf {Z}}) - h({\mathbf {Z}}_R) \big ] \Big \vert \nonumber \\&\quad \le \frac{m}{2R^d} \Vert h''\Vert _\infty \sqrt{ \sum _{i,j=1}^m \mathrm{Var}\Big ( \big \langle DF_R(t_i), - DL^{-1}F_R(t_j) \big \rangle _{\mathcal {H}} \Big ) } + \Big \vert {\mathbb {E}}\big [ h({\mathbf {Z}}) - h({\mathbf {Z}}_R) \big ] \Big \vert , \end{aligned}$$

with ${\mathbf {Z}}_R\sim N\big (0, {\mathcal {C}}_R \big )$, ${\mathbf {Z}}\sim N\big (0, {\mathcal {C}} \big )$ and $\Vert h'' \Vert _\infty = \sup \big \{ \big \vert \frac{\partial ^2}{\partial x_i \partial x_j} h(x) \big \vert : x\in {\mathbb {R}}^m, i,j=1, \dots , m\big \}$. It is clear that the second term in (4.35) tends to zero as $R\rightarrow \infty$. For the variance term in (4.35), taking advantage of Proposition 1.9 applied to $F=F_R(t_i)$ and $G=F_R(t_j)$ and using arguments analogous to those employed to derive (4.31), we obtain

$$\begin{aligned} \mathrm{Var}\Big ( \big \langle DF_R(t_i), - DL^{-1}F_R(t_j) \big \rangle _{\mathcal {H}} \Big ) \lesssim R^d. \end{aligned}$$

Thus, the first term in (4.35) is $O(R^{-d/2})$, implying that ${\mathbb {E}}\big [ h(R^{-d/2}{\mathbf {F}}_R) - h({\mathbf {Z}}) \big ]$ converges to zero as $R\rightarrow \infty$. This shows the convergence of the finite-dimensional distributions of $\{ R^{-d/2} F_R(t): t\in {\mathbb {R}}_+ \}$ to those of the centered Gaussian process ${\mathcal {G}}$, whose covariance structure is given by

$$\begin{aligned} {\mathbb {E}}\big [ {\mathcal {G}}(t) {\mathcal {G}}(s) \big ] = \omega _d \sum _{p\ge 1} p! \int _{{\mathbb {R}}^d} \big \langle {\widetilde{f}}_{t,x,p}, {\widetilde{f}}_{s,0,p} \big \rangle _{{\mathcal {H}}^{\otimes p}}dx, \; \text {for }s,t\in [0,\infty ). \end{aligned}$$

This concludes the proof of part (1) in Theorem 1.4. $\square$

#### Proofs in parts (2) and (3)

In part (2), in view of the dominance of the first chaos, we have already obtained in Sect. 4.1.2 that the finite-dimensional distributions of the process $\big \{R^{-d+\frac{\beta }{2} } F_R(t): t\in {\mathbb {R}}_+\big \}$ converge to those of a centered Gaussian process $\{{\mathcal {G}}_\beta (t) \}_{ t\in {\mathbb {R}}_+}$, whose covariance structure is given by (1.19). By the same reason, the convergence of the finite-dimensional distributions in part (3) follows from (4.24), (4.25), (4.27) and (4.28).

In this section, we show that:

4.36 $$\begin{aligned} d_{\mathrm{TV}}\big ( F_R(t)/\sigma _R(t), Z \big )\lesssim {\left\{ \begin{array}{ll} R^{-\beta /2} &{} \text {in part (2)}, \\ R^{-\frac{1}{2}(\beta _1+\beta _2)} &{} \text {in part (3) case }(a'),\\ R^{-(1+\beta )/2} &{} \text {in part (3) case }(b'), \end{array}\right. } \end{aligned}$$

where $Z\sim N(0,1)$. Taking into account (4.30) and the variance estimates in Sects. 4.1.2 and 4.1.3, in order to get (4.36) it suffices to show that, for $j\in \{1,2,3,4\}$ and for $R\ge t$,

4.37 $$\begin{aligned} {\mathcal {A}}_{R,j}\lesssim {\left\{ \begin{array}{ll} R^{4d-3\beta } &{} \text {in part (2)}, \\ R^{8- 3(\beta _1+\beta _2)} &{} \text {in case }(a')\text { of part (3),}\\ R^{5-3\beta } &{} \text {in case }(b')\text { of part (3).} \end{array}\right. } \end{aligned}$$

Since the total-variation distance is always bounded by one, the bound (4.36) still holds for $R<t$ by choosing the implicit constant large enough.

The rest of this section is then devoted to proving (4.37) for $R\ge t$ and for $j\in \{1,2,3,4\}$.

##### Proof of (4.37)

Let us first consider the term ${\mathcal {A}}_{R,1}$, which can be expressed as

$$\begin{aligned} {\mathcal {A}}_{R,1}&= \int _{[0,t]^6} drdr'dsds' d\theta d\theta ' \gamma _0(r-r') \gamma _0(s-s') \gamma _0(\theta - \theta ') {\mathbf {S}}_{1,R}. \end{aligned}$$

with

$$\begin{aligned} {\mathbf {S}}_{1,R}:&= \int _{{\mathbb {R}}^{6d}} dzdz' dydy' dwdw' \gamma (w-w') \gamma (y-y') \gamma (z-z') \int _{B_R^4} d\pmb {x_4} G_{t-r}(x_1-z) \\&\quad \times G_{r-\theta }(z-w) G_{t-s}(x_2-y) G_{s-\theta '}(y-w') G_{t-r'}(x_3- z') G_{t-s'}(x_4 - y'). \end{aligned}$$

From now on, when $$d=2$$, we write $$(w, w', y, y', z, z') =(w_1, w_2, w'_1, w'_2, y_1, y_2, y'_1, y'_2,z_1, z_2,z'_1, z'_2) $$ and then $$dy = dy_1 dy_2$$; note also that $$x_1,\dots , x_4$$ denote the dummy variables in $${\mathbb {R}}^d$$. By making the following change of variables

4.38 $$\begin{aligned} (z,z', y, y', w,w', x_1,x_2,x_3,x_4 ) \rightarrow R(z,z', y, y', w,w', x_1,x_2,x_3,x_4 ) \end{aligned}$$

and using the scaling property $G_{t}(Rz) = R^{1-d} G_{tR^{-1}}(z)$ for $d\in \{1,2\}$, we get

4.39 $$\begin{aligned}&{\mathbf {S}}_{1,R}=R^{6+4d} \int _{[-2,2]^{6d}} dzdz' dydy' dwdw' \gamma (Rw-Rw') \gamma (Ry-Ry') \gamma (Rz-Rz')\nonumber \\&\qquad \int _{B_1^4} d\pmb {x_4} \times G_{\frac{t-r}{R}}(x_1-z) G_{\frac{r-\theta }{R}}(z-w) G_{\frac{t-s}{R}}(x_2-y) G_{\frac{s-\theta '}{R}}(y-w') \nonumber \\&\qquad G_{\frac{t-r'}{R}}(x_3- z') G_{\frac{t-s'}{R}}(x_4 - y'). \end{aligned}$$

Note that we have replaced the integral domain ${\mathbb {R}}^{6d}$ by $[-2,2]^{6d}$ in (4.39) without changing the value of ${\mathbf {S}}_{1,R}$, because, for example, $x_1\in B_1$ and $|x_1-z| \le (t-r)/R$ implies $|z|\le 1 + tR^{-1}\le 2$ while $|z-w| \le (r-\theta )/R$ and $|x_1-z| \le (t-r)/R$ imply $|w|\le (t-\theta )R^{-1}+1 \le 2$.

In view of the expression of $\gamma$ in part (2) and part (3), we write, for $z\in {\mathbb {R}}^d$ ($z=(z_1,z_2)\in {\mathbb {R}}^2$ when $d=2$),

$$\begin{aligned} \gamma (Rz) = {\left\{ \begin{array}{ll} R^{-\beta } \gamma (z) &{} \text {in part (2)} ,\\ R^{-\beta _1-\beta _2} \gamma (z) &{} \text {in case }(a')\text { of part (3)}, \\ R^{-\beta } \gamma _1(Rz_1)\gamma _2(z_2) &{} \text {in case }(b')\text { of part (3)}, \end{array}\right. } \end{aligned}$$

and it is easy to see that

$$\begin{aligned}&\sup _{z'\in [-2,2]^d} \int _{[-2,2]^d}\gamma (Rz-Rz')dz \\&\quad \le {\left\{ \begin{array}{ll} {\displaystyle R^{-\beta }\int _{[-4,4]^d}\gamma (z)dz< \infty } &{} \text {in part (2)}, \\ \quad \\ {\displaystyle R^{-\beta _1-\beta _2}\int _{[-4,4]^d}\gamma (z)dz< \infty } &{} \text {in case }(a')\text { of part (3)}, \\ \quad \\ {\displaystyle R^{-\beta -1} \gamma _1({\mathbb {R}})\int _{-4}^4\gamma _2(s)ds < \infty } &{} \text {in case }(b')\text { of part (3)}. \end{array}\right. } \end{aligned}$$

To ease the notation, we just rewrite the above estimates as

4.40 $$\begin{aligned} \sup _{z'\in [-2,2]^d} \int _{[-2,2]^d}\gamma (Rz-Rz')dz \lesssim R^{-\alpha } \end{aligned}$$

with $\alpha = \beta$ in part (2), $\alpha =\beta _1+\beta _2$ in case $(a')$ of part (3), and $\alpha =1+\beta$ in case $(b')$ of part (3).

To estimate ${\mathcal {A}}_{R,1}$, we can use (4.40) to perform integration with respect to $dx_1, dx_2, dx_4$, $dy', dy, dw', dw$, $dz, dz', dx_3$ successively. More precisely, performing the integration with respect to $dx_1, dx_2, dx_4$ and using the fact

4.41 $$\begin{aligned} \sup _{ (s,z')\in [0,t]\times {\mathbb {R}}^d} \int _{{\mathbb {R}}^d}G_{s/R}(z-z') dz = t/R \end{aligned}$$

gives us

$$\begin{aligned} {\mathbf {S}}_{1,R}&\le R^{3+4d} t^3 \int _{[-2,2]^{6d}} dzdz' dydy' dwdw' \gamma (Rw-Rw') \\&\quad \gamma (Ry-Ry') \gamma (Rz-Rz') \int _{B_1} dx_3 \\&\qquad \times G_{\frac{r-\theta }{R}}(z-w) G_{\frac{s-\theta '}{R}}(y-w') G_{\frac{t-r'}{R}}(x_3- z') \\&\lesssim R^{3+4d} R^{-\alpha } \int _{[-2,2]^{5d}} dzdz' dy dwdw' \gamma (Rw-Rw') \gamma (Rz-Rz') \int _{B_1} dx_3 \\&\qquad \times G_{\frac{r-\theta }{R}}(z-w) G_{\frac{s-\theta '}{R}}(y-w') G_{\frac{t-r'}{R}}(x_3- z') \\&\qquad \text {by integrating out }dy'\text { and using }(4.40) \\&\lesssim R^{2+4d -\alpha } \int _{[-2,2]^{4d}} dzdz' dwdw' \gamma (Rw-Rw') \gamma (Rz-Rz') \int _{B_1} dx_3 \\&\qquad \times G_{\frac{r-\theta }{R}}(z-w) G_{\frac{t-r'}{R}}(x_3- z') \,\, \text {by integrating out }dy\text { and using }(4.41) \\&\lesssim R^{2+4d -2\alpha } \! \int _{[-2,2]^{3d}} dzdz' dw \gamma (Rz-Rz') \!\int _{B_1} dx_3 \! G_{\frac{r-\theta }{R}}(z-w) G_{\frac{t-r'}{R}}(x_3- z') \end{aligned}$$

by integrating out $dw'$ and using (4.40); then, using (4.41) to integrate out *dw*

$$\begin{aligned} \lesssim R^{1+4d -2\alpha } \int _{[-2,2]^{2d}} dzdz' \gamma (Rz-Rz') \int _{B_1} dx_3 G_{\frac{t-r'}{R}}(x_3- z') \lesssim R^{4d -3\alpha } \end{aligned}$$

where the last inequality is obtained by integrating out $dz, dz'$, $dx_3$ one by one and using (4.40) and (4.41). The bound

$$\begin{aligned} {\mathbf {S}}_{1,R} \lesssim R^{4d -3\alpha } = {\left\{ \begin{array}{ll} R^{4d-3\beta } &{} \text {in part (2)}, \\ R^{8-3\beta _1-3\beta _2} &{} \text {in cae }(a')\text { of part (3)}, \\ R^{5-3\beta } &{} \text {in cae }(b')\text { of part (3)} \end{array}\right. } \end{aligned}$$

is uniform over $(r,r',s,s' ,\theta ,\theta ')\in [0,t]^6$, and hence we obtain (4.37) for $j=1$. For the other terms ${\mathcal {A}}_{R,2}, {\mathcal {A}}_{R,3}$ and ${\mathcal {A}}_{R,4}$, the arguments are the same: We first go through the same change of variables (4.38) to obtain terms ${\mathbf {S}}_{j, R}$ similar to ${\mathbf {S}}_{1, R}$ in (4.39), and then use the facts (4.40) and (4.41) to perform one-by-one integration with respect to the variables

$$\begin{aligned} {\left\{ \begin{array}{ll} dx_1, dx_3,dz', dz, dx_2, dw, dw', dy, dy', dx_4 \quad \text {for estimating }{\mathcal {A}}_{R,2} \\ dx_4, dy', dx_2, dy, dw', dx_1, dw, dz, dz', dx_3\quad \text {for estimating }{\mathcal {A}}_{R,3} \\ dx_1, dx_3, dx_2, dz', dz, dw, dw', dy, dy', dx_4\quad \text {for estimating }{\mathcal {A}}_{R,4} \end{array}\right. }. \end{aligned}$$

This concludes the proof of (4.37) and hence completes the proof of (4.36). $\square$

### Tightness

This section is devoted to establishing the tightness in Theorem 1.4. This, together with the results in Sects. 4.1 and 4.2 will conclude the proof of Theorem 1.4. To get the tightness, we appeal to the criterion of Kolmogorov-Chentsov (see *e.g.* [17, Corollary 16.9]). Put

4.42 $$\begin{aligned} \sigma _R = {\left\{ \begin{array}{ll} R^{d/2} &{}\text {in part (1) of Theorem}~1.4 \\ R^{d-\frac{\beta }{2}} &{}\text {in part (2) of Theorem}~1.4 \\ R^{2- \frac{1}{2}(\beta _1+\beta _2) } &{}\text {in part .}(3)-(a')\text { of Theorem}~1.4 \\ R^{(3- \beta )/2} &{}\text {in part }(3)-(b')\text { of Theorem}~1.4 \end{array}\right. } \end{aligned}$$

and we will show, for any fixed $T>0$, that the following inequality holds for any integer $k\ge 2$ and any $0< s < t \le T\le R$:

4.43 $$\begin{aligned} \big \Vert F_R(t)- F_R(s) \big \Vert _k \lesssim (t-s) \sigma _R, \end{aligned}$$

where the implicit constant does not depend on *R*, *s* or *t*. This moment estimate (4.43) ensures the tightness of $\big \{ \sigma _R^{-1} F_R(t): t\in [0,T]\big \}$ for any fixed $T>0$ and, therefore, the desired tightness on ${\mathbb {R}}_+$ holds.

To show the above moment estimate (4.43) for the increment $F_R(t)- F_R(s)$, we begin with the chaos expansion

$$\begin{aligned} F_R(t) - F_R(s) = \sum _{n\ge 1} I_n\left( \int _{B_R}dx [ f_{t,x,n} - f_{s,x,n}] \right) = \sum _{n\ge 1} I_n\big ( g_{n,R}\big ), \end{aligned}$$

where *s*, *t* are fixed, so we leave them out of the subscript of the kernel $g_{n,R}$ and

4.44 $$\begin{aligned} g_{n,R}(\pmb {s_n} , \pmb {y_n}) = \Big [ \varphi _{t,R}(s_1,y_1) - \varphi _{s,R}(s_1,y_1) \Big ] \prod _{j=1}^{n-1}G_{s_j- s_{j+1}}(y_j-y_{j+1}) \end{aligned}$$

with $\prod _{j=1}^0 =1$ and $\varphi _{t,R}(r,y) := \int _{B_R} G_{t-r}(x-y) dx$. The rest of this section is then devoted to proving (4.43).

#### Proof of (4.43)

By the triangle inequality and using the moment estimate (2.15), we get, for any $k\in [2,\infty )$,

$$\begin{aligned} \big \Vert F_R(t) - F_R(s) \big \Vert _k \le \sum _{n\ge 1} (k-1)^{n/2} \left\| I_n\left( g_{n,R} \right) \right\| _2. \end{aligned}$$

Note that the kernel $g_{n,R} =0$ outside $[0,t]^n \times {\mathbb {R}}^{dn}$. Then, using (2.8) and (2.13), we can write

$$\begin{aligned} \big \Vert F_R(t) - F_R(s) \big \Vert _k \le \sum _{n\ge 1} \big [ \Gamma _t (k-1) \big ]^{n/2} \Big ( n! \Vert {\widetilde{g}}_{n,R} \Vert _{{\mathcal {H}}_0^{\otimes n}}^2 \Big )^{1/2}, \end{aligned}$$

where ${\widetilde{g}}_{n,R}$ is the canonical symmetrization of $g_{n,R}$:

$$\begin{aligned} {\widetilde{g}}_{n,R}( \pmb {s_n} , \pmb {y_n} )= & {} \frac{1}{n!} \sum _{\sigma \in {\mathfrak {S}}_n} \Big [ \varphi _{t,R}(s_{\sigma (1)} ,y_{\sigma (1)}) - \varphi _{s,R}(s_{\sigma (1)} ,y_{\sigma (1)}) \Big ] \\&\times \prod _{j=1}^{n-1}G_{s_{\sigma (j)}- s_{\sigma (j+1)}}(y_{\sigma (j)}-y_{\sigma (j+1)}). \end{aligned}$$

With the convention (1.6) in mind, we can write

$$\begin{aligned}&n! \Vert {\widetilde{g}}_{n,R} \Vert _{{\mathcal {H}}_0^{\otimes n}}^2 = \int _{t>s_1>\cdots>s_n >0} d\pmb {s_n} \int _{{\mathbb {R}}^{2nd}} \Big [ \varphi _{t,R}(s_1,y_1) - \varphi _{s,R}(s_1,y_1) \Big ] \\&\quad \times \left( \prod _{j=1}^{n-1}G_{s_j- s_{j+1}}(y_j-y_{j+1}) \right) \times \Big [ \varphi _{t,R}(s_1,y'_1) - \varphi _{s,R}(s_1,y'_1) \Big ] \\&\qquad \times \left( \prod _{j=1}^{n-1}G_{s_j- s_{j+1}}(y'_j-y'_{j+1}) \right) \prod _{j=1}^n \gamma (y_j - y_j') dy_j dy_j'. \end{aligned}$$

Then, using Fourier transform, we can rewrite $n! \Vert {\widetilde{g}}_{n,R} \Vert _{{\mathcal {H}}_0^{\otimes n}}^2$ as follows:

4.45 $$\begin{aligned}&n! \Vert {\widetilde{g}}_{n,R} \Vert _{{\mathcal {H}}_0^{\otimes n}}^2 = \int _{t>s_1>\cdots>s_n >0} d\pmb {s_n} \int _{{\mathbb {R}}^{nd}} \mu (d\pmb {\xi _p} ) \big \vert {\mathcal {F}}{\mathbf {1}}_{B_R}\big \vert ^2(\xi _1+\cdots +\xi _p) \nonumber \\&\quad \times \big \vert {\widehat{G}}_{t-t_1}(\xi _1 + \cdots + \xi _p)- {\widehat{G}}_{s-t_1}(\xi _1 + \cdots + \xi _p) \big \vert ^2 \nonumber \\&\quad \times \prod _{j=1}^{n-1} \big \vert {\widehat{G}}_{s_j- s_{j+1}} \big \vert ^2(\xi _{j+1} + \cdots + \xi _p) . \nonumber \\ \end{aligned}$$

Recall the expression (2.29) ${\widehat{G}}_t(\xi )= \frac{\sin (t|\xi |)}{|\xi |}$ and note that it is a 1-Lipschitz function in the variable *t*, uniformly over $\xi \in {\mathbb {R}}^d$. Then

$$\begin{aligned} \big \vert {\widehat{G}}_{t-t_1}(\xi _1 + \cdots + \xi _p)- {\widehat{G}}_{s-t_1}(\xi _1 + \cdots + \xi _p) \big \vert ^2 \le (t-s)^2. \end{aligned}$$

Therefore, plugging this inequality into (4.45) and then applying Lemma 2.6 yields

$$\begin{aligned} n! \Vert {\widetilde{g}}_{n,R} \Vert _{{\mathcal {H}}_0^{\otimes n}}^2&\le (t-s)^2 \int _{t>s_1>\cdots>s_n >0} d\pmb {s_n} \left( \int _{{\mathbb {R}}^{d}} \mu (d\xi ) \big \vert {\mathcal {F}}{\mathbf {1}}_{B_R}\big \vert ^2(\xi )\right) \\&\quad \times \prod _{j=1}^{n-1} \int _{{\mathbb {R}}^d}\mu (d\xi _j) \big \vert {\widehat{G}}_{s_j- s_{j+1}} \big \vert ^2(\xi _j) \\&\le (t-s)^2 \frac{t^n}{n!} \left( 2(t^2\vee 1)\int _{{\mathbb {R}}^{d}} \frac{\mu (d\xi )}{1+ |\xi |^2} \right) ^{n-1} \int _{{\mathbb {R}}^{d}} \mu (d\xi ) \big \vert {\mathcal {F}}{\mathbf {1}}_{B_R}\big \vert ^2(\xi ), \end{aligned}$$

which is finite since ${\mathbf {1}}_{B_R}\in {\mathcal {P}}_0$. Using Fourier transform, we can write

$$\begin{aligned} \int _{{\mathbb {R}}^{d}} \mu (d\xi ) \big \vert {\mathcal {F}}{\mathbf {1}}_{B_R}\big \vert ^2(\xi ) = \int _{{\mathbb {R}}^{2d}} {\mathbf {1}}_{B_R}(x) {\mathbf {1}}_{B_R}(y) \gamma (x-y)dxdy. \end{aligned}$$

Now let us consider the cases in (4.42).

In part (1) where $\gamma \in L^1({\mathbb {R}}^d)$,

$$\begin{aligned} \int _{{\mathbb {R}}^{2d}} {\mathbf {1}}_{B_R}(x) {\mathbf {1}}_{B_R}(y) \gamma (x-y)dxdy \le \gamma ({\mathbb {R}}^d) \omega _d R^d \lesssim \sigma _R^2. \end{aligned}$$

In the other cases, we can make the change of variables $(x,y)\rightarrow R(x,y)$ to obtain

$$\begin{aligned} \int _{{\mathbb {R}}^{2d}} {\mathbf {1}}_{B_R}(x) {\mathbf {1}}_{B_R}(y) \gamma (x-y)dxdy&= R^{2d} \int _{{\mathbb {R}}^{2d}} {\mathbf {1}}_{B_1}(x) {\mathbf {1}}_{B_1}(y) \gamma (Rx-Ry)dxdy \\&\lesssim R^{2d-\alpha } = \sigma _R^2, \end{aligned}$$

using (4.40) with $\alpha = \beta$ in part (2), $\alpha =\beta _1+\beta _2$ in case $(a')$, and $\alpha =1+\beta$ in case $(b')$.

As a consequence, we get

$$\begin{aligned} n! \Vert {\widetilde{g}}_{n,R} \Vert _{{\mathcal {H}}_0^{\otimes n}}^2 \le \frac{C^n}{n!} \sigma _R^2 (t-s)^2, \end{aligned}$$

and therefore,

$$\begin{aligned} \big \Vert F_R(t) - F_R(s) \big \Vert _k \le |t - s| \sigma _R \sum _{n\ge 1} \big [C \Gamma _t (k-1) \big ]^{n/2} \frac{1}{\sqrt{n!} }, \end{aligned}$$

which leads to (4.43). $\square$

## Proof of Theorem 1.10

We argue as in the proof of Theorem 1.2 of [2]. As we explained in the introduction, it suffices to show that for each $m\ge 1$,

$$\begin{aligned} \Vert D u(t,x)\Vert _{{\mathcal {H}}}>0 \quad \text{ a.s. } \text{ on } \ \Omega _m, \end{aligned}$$

where $$\Omega _m =\{ |u(t,x) | \ge 1/m\}$$.

We claim that, almost surely, the function $(s,y) \mapsto D_{s,y}u(t,x)$ satisfies the assumptions of Lemma A.1. Indeed, for $d=2$, by Minkowski’s inequality and the estimate (1.11), we have

$$\begin{aligned} {\mathbb {E}}\left( \int _0^t ds \left( \int _{{\mathbb {R}}^2} | D_{s,y} u(t,x) |^{2q} dy \right) ^{ 1/q} \right)&\le \int _0^t ds \left( \int _{{\mathbb {R}}^2} \Big | {\mathbb {E}}\big [ | D_{s,y} u(t,x) |^{2} \big ] \Big |^q dy \right) ^{ 1/q} \\&\le C \int _0^t ds \left( \int _{{\mathbb {R}}^2} G^{2q}_{t-s} (x-y)dy \right) ^{ 1/q} <\infty . \end{aligned}$$

For $d=1$, again by the estimate (1.11),

$$\begin{aligned} {\mathbb {E}}\left( \int _0^t ds \left( \int _{{\mathbb {R}}} | D_{s,y} u(t,x) |^{2} dy \right) \right) \le C \int _0^t ds \int _{{\mathbb {R}}} G^{2}_{t-s} (x-y)dy <\infty . \end{aligned}$$

Moreover, $(s,y) \mapsto D_{s,y}u(t,x)$ has compact support on $[0,t]\times B_M$ for some $M>0$. As a consequence, by Lemma A.1, it suffices to prove that

5.1 $$\begin{aligned}&\int _0^t \Vert D_{r,\bullet }u(t,x)\Vert _0^2 dr \nonumber \\&\qquad = \int _0^t \int _{{\mathbb {R}}^{2d}}D_{r,z}u(t,x) D_{r,z'}u(t,x)\gamma (z-z')dzdz'dr>0 ~ \text{ a.s. } \text{ on } \Omega _m.\nonumber \\ \end{aligned}$$

As in the proof of Lemma 5.1 of [2], Corollaries 3.3 and 3.4 allow us to infer that the ${\mathcal {H}}\otimes {\mathcal {P}}_0$-valued process $K^{(r)}$ defined by

$$\begin{aligned} K^{(r)}(s,y,z)=G_{t-s}(x-y)D_{r,z}u(s,y) \end{aligned}$$

belongs to the space ${\mathbb {D}}^{1,2}({\mathcal {H}}\otimes {\mathcal {P}}_0)$. This is because, using Corollary 3.3, we can write

$$\begin{aligned}&{\mathbb {E}}\big ( \Vert K^{(r)}\Vert _{{\mathcal {H}}\otimes {\mathcal {P}}_0}^2 \big ) \\&\quad = \int _{[r,t]^2} \int _{{\mathbb {R}}^{2d}} G_{t-s}(x-y)G_{t-s'}(x-y') {\mathbb {E}}\Big ( \big \langle D_{r,\bullet }u(s,y), D_{r,\bullet } u(s',y') \big \rangle _0 \Big )\\&\qquad \times \gamma _0(s-s') \gamma (y-y') dydy' dsds'\\&\quad \le C\int _{[r,t]^2} \int _{{\mathbb {R}}^{2d}} G_{t-s}(x-y)G_{t-s'}(x-y')\\&\qquad \gamma _0(s-s') \gamma (y-y') dydy' dsds' <\infty , \end{aligned}$$

and in the same way, using Corollary 3.4 we can show that ${\mathbb {E}}\big ( \Vert DK^{(r)}\Vert _{ {\mathcal {H}}\otimes {\mathcal {H}}\otimes {\mathcal {P}}_0}^2 \big ) <\infty$. Therefore, the process $K^{(r)}$ belongs to the domain of the ${\mathcal {P}}_0$-valued Skorokhod integral, denoted by $\overline{\delta }$. Then, using the same arguments as in the proof of Proposition 5.2 of [2], replacing $L^2({\mathbb {R}})$ by ${\mathcal {P}}_0$, we can show that for any $r \in [0,t]$, the following equation holds in $L^2(\Omega ;{\mathcal {P}}_0)$:

5.2 $$\begin{aligned} D_{r,\bullet }u(t,x)=G_{t-r}(x-\bullet )u(r,\bullet )+\int _r^t \int _{{\mathbb {R}}^d}G_{t-s}(x-y)D_{r,\bullet }u(s,y)W(\overline{\delta } s, \overline{\delta }y).\nonumber \\ \end{aligned}$$

Let $\delta \in (0,t \wedge 1)$ be arbitrary. Due to relation (5.2) we have, almost surely,

5.3 $$\begin{aligned} \int _{0}^t \Vert D_{r, \bullet } u(t,x)\Vert ^2_{0}\, dr&\ge \int _{t-\delta }^t \Vert D_{r,\bullet } u(t,x)\Vert ^2_{0}\, dr \nonumber \\&\ge \frac{1}{2} \int _{t-\delta }^t \Vert G_{t-r}(x-\bullet ) u(r,\bullet )\Vert ^2_{0} \, dr - I(\delta ), \end{aligned}$$

where

$$\begin{aligned} I(\delta )&= \int _{t-\delta }^t \left\| \int _{r}^t \int _{{\mathbb {R}}^d} G_{t-s}(x-y) D_{r, \bullet } u(s,y) W(\overline{\delta } s, \overline{\delta } y) \right\| ^2_{0} \, dr\\&= \int _{t-\delta }^t \left\| \int _{t-\delta }^t \int _{{\mathbb {R}}^d} G_{t-s}(x-y) D_{r, \bullet } u(s,y) W(\overline{\delta } s, \overline{\delta } y)\right\| ^2_{0} \, dr. \end{aligned}$$

On the event $\Omega _m=\{ | u(t,x) | \ge 1/m\}$, we have

$$\begin{aligned}&\int _{t-\delta }^t \Vert G_{t-r}(x-\bullet ) u(r,\bullet )\Vert _0^2 dr\\&\quad = \int _{t-\delta }^t \int _{{\mathbb {R}}^{2d}} G_{t-r}(x-z)G_{t-r}(x-z') u(r,z) u(r,z')\gamma (z-z')dzdz' dr \\&\quad = \int _{t-\delta }^t \int _{{\mathbb {R}}^{2d}} G_{t-r}(x-z)G_{t-r}(x-z') u(t,x)^2 \gamma (z-z')dzdz' dr \\&\qquad -\!\int _{t-\delta }^t \! \!\int _{{\mathbb {R}}^{2d}} G_{t-r}(x\!-\!z)G_{t-r}(x\!-\!z') \big [ u(t,x)^2\!-\!u(r,z)u(r,z')\big ] \gamma (z-z')dzdz' dr \\&\quad \ge \frac{1}{m^2} \psi _0(\delta ) - J(\delta ), \end{aligned}$$

where

$$\begin{aligned} \psi _0(\delta )&:=\int _{t-\delta }^t \int _{{\mathbb {R}}^{2d}} G_{t-r}(x-z)G_{t-r}(x-z')\gamma (z-z') dzdz' dr\\&= \int _0^\delta \int _{{\mathbb {R}}^{2d}} G_r(z)G_r(z')\gamma (z-z')dz dz'dr \end{aligned}$$

and

$$\begin{aligned} J(\delta )&:= \int _{t-\delta }^t \int _{{\mathbb {R}}^{2d}} G_{t-r}(x-z)G_{t-r}(x-z')\gamma (z-z')\\&\quad \Big ( u(t,x)^2 - u(r,z)u(r,z')\Big ) dz dz' dr. \end{aligned}$$

Coming back to (5.3), we can write

5.4 $$\begin{aligned} \int _0^t \Vert D_{r,\bullet }u(t,x)\Vert _0^2 dr \ge \frac{1}{2 m^2}\psi _0(\delta )- \frac{1}{2} J(\delta ) - I(\delta ) \quad \text{ on } \quad \Omega _m. \end{aligned}$$

We now give upper bounds for the first moments of $J(\delta )$ and $I(\delta )$. We will use the following facts, which were proved in [3]:

$$\begin{aligned} C_t^*&:=\sup _{(s,y) \in [0,t] \times {\mathbb {R}}^d}\Vert u(s,y)\Vert _2<\infty \qquad (\text {see also }(3.16)\text { in Remark}~3.1) \\ g_{t,x}(\delta )&:= \sup _{|t-s|<\delta } \sup _{|x-y|<\delta } \Vert u(t,x)-u(s,y)\Vert _2 \rightarrow 0 \quad \text{ as } \ \delta \rightarrow 0. \end{aligned}$$

We first treat $J(\delta )$. By Cauchy-Schwarz inequality, for any $r\in [0,t]$ and $z,z' \in {\mathbb {R}}^2$,

$$\begin{aligned} {\mathbb {E}}\big [ |u(t,x)^2-u(r,z)u(r,z')| \big ]&\le \Vert u(t,x)\Vert _2 \Vert u(t,x)-u(r,z)\Vert _2\\&\quad + \Vert u(r,z)\Vert _2 \Vert u(t,x)-u(r,z')\Vert _2 \\&\le C_t^* \Big ( \Vert u(t,x)-u(r,z)\Vert _2+ \Vert u(t,x)-u(r,z')\Vert _2 \Big ). \end{aligned}$$

Since $G_{t-r}(x-z)$ contains the indicator of the set $\{|x-z|<t-r\}$, we obtain:

$$\begin{aligned} {\mathbb {E}}(|J(\delta )|)&\le 2C_t^*\int _{t-\delta }^t \int _{{\mathbb {R}}^{2d}} G_{t-r}(x-z)G_{t-r}(x-z')\gamma (z-z')\Vert u(t,x)\\&\qquad -u(r,z)\Vert _2dz dz' dr \\&\le 2C_t^* \int _{t-\delta }^t \int _{{\mathbb {R}}^{2d}} G_{t-r}(x-z)G_{t-r}(x-z')\gamma (z-z') \\&\quad \sup _{ \begin{array}{c} t-\delta<s<t \\ |x-y|<\delta \end{array}}\Vert u(t,x)-u(s,y)\Vert _2dz dz' dr. \end{aligned}$$

It follows that

5.5 $$\begin{aligned} {\mathbb {E}}(|J(\delta )|)\le 2 C_t^* g_{t,x}(\delta ) \psi _0(\delta ). \end{aligned}$$

Next, we treat $I(\delta )$. Applying Proposition 6.2 of [1] to the ${\mathcal {P}}_0$-valued process

$$\begin{aligned} U(s,y)={\mathbf {1}}_{[t-\delta ,t]}(s)G_{t-s}(x-y)D_{r,\bullet }u(s,y) \end{aligned}$$

we obtain

$$\begin{aligned} {\mathbb {E}}(\Vert \overline{\delta }(U)\Vert _0^2) \le {\mathbb {E}}(\Vert U\Vert _{{\mathcal {H}}\otimes {\mathcal {P}}_0}^2)+{\mathbb {E}}(\Vert DU\Vert _{{\mathcal {H}}\otimes {\mathcal {H}}\otimes {\mathcal {P}}_0}^2). \end{aligned}$$

We have,

$$\begin{aligned} {\mathbb {E}}(\Vert U\Vert _{{\mathcal {H}}\otimes {\mathcal {P}}_0}^2)&={\mathbb {E}}\Bigg ( \int _{[t-\delta ,t]^2} \int _{{\mathbb {R}}^{2d}} G_{t-s}(x-y)G_{t-s'}(x-y') \gamma _0(s-s')\gamma (y-y') \\&\quad \times \big \langle D_{r,\bullet }u(s,y),D_{r,\bullet }u(s',y') \big \rangle _0 dydy' dsds' \Bigg ) \end{aligned}$$

and

$$\begin{aligned}&{\mathbb {E}}(\Vert DU\Vert _{{\mathcal {H}}\otimes {\mathcal {H}}\otimes {\mathcal {P}}_0}^2)\\&\quad ={\mathbb {E}}\Bigg (\int _{[t-\delta ,t]^2} \int _{[0,r]^2} \int _{{\mathbb {R}}^{4d}}G_{t-s}(x-y) G_{t-s'}(x-y') \gamma _0(s-s')\gamma (y-y') \\&\qquad \times \big \langle D^2_{(\theta ,w),(r,\bullet )}u(s,y),D_{(\theta ',w'),(r,\bullet )}u(s',y')\big \rangle _0\, \gamma _0(\theta -\theta ')\\&\qquad \times \gamma (w-w') dwdw' dydy' d\theta d\theta ' dsds' \Bigg )\\&\quad ={\mathbb {E}}\Bigg (\int _{[t-\delta ,t]^2} \int _{{\mathbb {R}}^{2d}}G_{t-s}(x-y) G_{t-s'}(x-y') \gamma _0(s-s')\gamma (y-y') \\&\qquad \times \big \langle DD_{r,\bullet }u(s,y), DD_{r,\bullet }u(s',y')\big \rangle _{{\mathcal {H}}\otimes {\mathcal {P}}_0} dydy' ds ds'\Bigg ). \end{aligned}$$

Hence, $${\mathbb {E}}(I(\delta )) \le I_1(\delta )+I_2(\delta )$$, where

$$\begin{aligned} I_1(\delta )&:={\mathbb {E}}\Bigg ( \int _{[t-\delta ,t]^3} \int _{{\mathbb {R}}^{2d}} G_{t-s}(x-y)G_{t-s'}(x-y') \gamma _0(s-s')\gamma (y-y') \\&\qquad \times \big \langle D_{r,\bullet }u(s,y),D_{r,\bullet }u(s',y') \big \rangle _0 dydy' dsds'dr \Bigg ) \end{aligned}$$

and

$$\begin{aligned} I_2(\delta )&:={\mathbb {E}}\Bigg ( \int _{[t-\delta ,t]^3} \int _{{\mathbb {R}}^{2d}}G_{t-s}(x-y) G_{t-s'}(x-y') \gamma _0(s-s')\gamma (y-y') \\&\qquad \times \langle DD_{r,\bullet } u(s,y), DD_{r,\bullet }u(s',y')\rangle _{{\mathcal {H}}\otimes {\mathcal {P}}_0} dydy' ds ds'dr \Bigg ). \end{aligned}$$

Using Cauchy-Schwarz inequality and Corollaries 3.3 and 3.4 , we obtain:

$$\begin{aligned}&{\mathbb {E}}\Big ( \big |\langle D_{r,\bullet }u(s,y),D_{r,\bullet }u(s',y')\rangle _0 \big |\Big )\le C_t \quad \text{ and } \\&\quad {\mathbb {E}}\Big ( \big | \langle DD_{r,\bullet }u(s,y), DD_{r,\bullet }u(s',y')\rangle _{{\mathcal {H}}\otimes {\mathcal {P}}_0}\big | \Big )\le C_t''. \end{aligned}$$

Hence,

5.6 $$\begin{aligned} {\mathbb {E}}[I(\delta )] \le (C_t+C_t'')\delta \phi (\delta ), \end{aligned}$$

where

5.7 $$\begin{aligned} \phi (\delta ):= & {} \int _{[t-\delta ,t]^2} \int _{{\mathbb {R}}^{2d}}G_{t-s}(x-y)G_{t-s'}(x-y')\gamma _0(s-s')\gamma (y-y')dydy'ds ds'\nonumber \\= & {} \int _{[0,\delta ]^2}\int _{{\mathbb {R}}^{2d}} G_s(y)G_{s'}(y')\gamma _0(s-s')\gamma (y-y')dydy'ds ds'. \end{aligned}$$

Using (5.4), (5.5) and (5.6), we conclude the proof as follows. For any $n\ge 1$,

$$\begin{aligned}&{\mathbb {P}}\left( \left\{ \int _{0}^t \Vert D_{r,\bullet } u(t,x)\Vert _0^2 \, dr <\frac{1}{n}\right\} \cap \Omega _m \right) \le {\mathbb {P}}\left( I(\delta ) + \frac{1}{2} J(\delta ) > \frac{1}{2m^2}\psi _0(\delta ) - \frac{1}{n} \right) \\&\quad \le \left( \frac{1}{2m^2} \psi _0(\delta ) - \frac{1}{n}\right) ^{-1} \Big ({\mathbb {E}}[I(\delta )] + \frac{1}{2} {\mathbb {E}}[|J(\delta )|]\Big ) \\&\quad \le \frac{ (C_t+C_t'') \delta \phi (\delta ) + C_t^* g_{t,x}(\delta )\psi _0(\delta ) }{ \frac{1}{2m^2} \psi _0(\delta ) - \frac{1}{n} }. \end{aligned}$$

Letting $n\rightarrow \infty$, we obtain:

$$\begin{aligned} {\mathbb {P}}\left( \left\{ \int _{0}^t \Vert D_{r,\bullet } u(t,x)\Vert _0^2 dr =0 \right\} \cap \Omega _m \right) \le 2m^2 \Big ((C_t+C_t'') \delta \frac{\phi (\delta )}{\psi _0(\delta )} + C_t^* g_{t,x}(\delta )\Big ). \end{aligned}$$

Note that using Fourier transform and the expression (2.29), we can rewrite (5.7) as

$$\begin{aligned} \phi (\delta )&= \int _{[0,\delta ]^2}\int _{{\mathbb {R}}^{d}} {\widehat{G}}_s(\xi ) {\widehat{G}}_{s'}(\xi )\gamma _0(s-s') \mu (d\xi ) ds ds' \\&\le \int _{[0,\delta ]^2}\int _{{\mathbb {R}}^{d}} \frac{1}{2} \Big [ {\widehat{G}}_s(\xi )^2 + {\widehat{G}}_{s'}(\xi )^2 \Big ]\gamma _0(s-s') \mu (d\xi ) ds ds' \\&\le \Gamma _\delta \int _{[0,\delta ]}\int _{{\mathbb {R}}^{d}} {\widehat{G}}_s(\xi )^2 \mu (d\xi ) ds, \end{aligned}$$

where $\Gamma _{\delta }=2\int _0^{\delta }\gamma _0(s)ds$. That is, we have $\phi (\delta ) \le \Gamma _{\delta } \psi _0(\delta )$. Finally taking $\delta \rightarrow 0$ proves (5.1), since $g_{t,x}(\delta )\rightarrow 0$ and $\delta \frac{\phi (\delta )}{\psi _0(\delta )} \le \delta \Gamma _\delta \rightarrow 0$ as $\delta \rightarrow 0$. $\square$

## Appendix

### Auxiliary Results

Let $d=2$ and assume Hypothesis $\mathbf{(H1)}$. Suppose that $S: {\mathbb {R}}_+\times {\mathbb {R}}^2 \rightarrow {\mathbb {R}}$ is a measurable function such that $S\in L^{2}( {\mathbb {R}}_+; L^{2q} ({\mathbb {R}}^2))$, where *q* is given in (2.20) in cases (a) and (b) and it is given in (2.23) in case (c). We assume also that *S* has support in $[0,T]\times B_M$ for some $M>0$. We claim that *S* belongs to ${\mathcal {H}}$ and the following estimates hold true:

$$\begin{aligned} \Vert S \Vert _{{\mathcal {H}}} \le \sqrt{\Gamma _T} \Vert S \Vert _{{\mathcal {H}}_0} \le \sqrt{\Gamma _T D_\gamma } \Vert S\Vert _{ L^{2}( {\mathbb {R}}_+; L^{2q} ({\mathbb {R}}^2))}. \end{aligned}$$

Indeed, the first inequality is due to (2.13) and the second one follows from (2.25).

For $d=1$, if $S\in L^{2}( {\mathbb {R}}_+ \times {\mathbb {R}})$ has support in $[0,T]\times B_M$ for some $M>0$, then $S\in {\mathcal {H}}$ and the following estimates hold true:

$$\begin{aligned} \Vert S \Vert _{{\mathcal {H}}}\le & {} \sqrt{\Gamma _T} \Vert S \Vert _{{\mathcal {H}}_0} \le \sqrt{\Gamma _T \Vert \gamma {\mathbf {1}}_{B_{2M}} \Vert _{L^1({\mathbb {R}})} } \Vert S\Vert _{ L^{2}( {\mathbb {R}}_+\times {\mathbb {R}})}. \end{aligned}$$

Indeed, the first inequality is due to (2.13) and the second one follows from

$$\begin{aligned} \Vert S \Vert _{{\mathcal {H}}_0}^2&= \int _0^T \int _{{\mathbb {R}}^2} S(t,y) S(t,y') \gamma (y-y') dydy' dt \\&\le \int _0^T \int _{{\mathbb {R}}^2} \frac{ S^2(t,y) + S^2(t,y') }{2} \gamma (y-y') dydy' dt \end{aligned}$$

and

$$\begin{aligned} \sup _{y'\in B_M}\int _{B_M} \gamma (y-y') dy \le \int _{B_{2M}} \gamma (y) dy. \end{aligned}$$

Let us recall the Hypothesis $\mathbf{(H2)}$: The measures $\mu _0$ and $\mu$ such that $\gamma _0 ={\mathcal {F}}\mu _0$ and $\gamma = {\mathcal {F}} \mu$ are absolutely continuous with respect to the Lebesgue measures with strictly positive densities.

#### Lemma A.1

Fix $d\in \{1,2\}$ and assume that the Hypothesis $\mathbf{(H2)}$ holds. Let the Hypothesis $\mathbf{(H1)}$ hold if in addition $d=2$. Suppose that the function $S: {\mathbb {R}}_{+} \times {\mathbb {R}}^d\rightarrow {\mathbb {R}}$ has support in $[0,T]\times B_M$ for some $M>0$ and $S\in L^{2}\big ( {\mathbb {R}}_+; L^{2q}({\mathbb {R}}^d) \big )$, where

$$\begin{aligned} {\left\{ \begin{array}{ll} q\text { is given by }(2.20)\text { in cases }{(\texttt {a})}\text { and }{(\texttt {b})}\text { and by }(2.23)\text { in case }{(\texttt {c})} \text { if }d=2, \\ q=1\text { if }d=1. \end{array}\right. } \end{aligned}$$

If

A.1 $$\begin{aligned} I:=\int _0^{T}\int _{{\mathbb {R}}^d}\int _{{\mathbb {R}}^d} S(t,x) S(t,y)\gamma (x-y)dxdydt>0, \end{aligned}$$

then $$\Vert S\Vert _{{\mathcal {H}}}>0$$.

#### Proof

Suppose that $\Vert S\Vert _{{\mathcal {H}}}=0$. There exists a sequence of smooth functions $(\psi _k)_{k\ge 1}$ in $C^\infty ( {\mathbb {R}}_+\times {\mathbb {R}}^d)$, with support in $[0,T]\times B_M$, which converges to *S* in $L^2({\mathbb {R}}_+; L^{2q}({\mathbb {R}}^d))$. Then,

$$\begin{aligned} 0=\Vert S \Vert _{{\mathcal {H}}}^2 = \lim _{k\rightarrow \infty } \Vert \psi _k \Vert ^2_{{\mathcal {H}}} =\lim _{k\rightarrow \infty } \int _{{\mathbb {R}}_+\times {\mathbb {R}}^d} | {\mathcal {F}} \psi _k(\tau , \xi ) |^2 \mu _0(d\tau ) \mu (d\xi ), \end{aligned}$$

where $\gamma _0 ={\mathcal {F}}\mu _0$, $\gamma = {\mathcal {F}} \mu$ and ${\mathcal {F}} \psi _k$ stands for the Fourier transform of $\psi _k$ in space-time variables *in this proof*. By choosing a subsequence $(k_j)_{j\ge 1}$ we have that

$$\begin{aligned} \lim _{j\rightarrow \infty } {\mathcal {F}} \psi _{k_j}(\tau , \xi ) =0 \end{aligned}$$

for $\mu _0 \otimes \mu$-almost all $(\tau , \xi )$. On the other hand, keeping in mind that the supports of $S,\psi _k$ are contained in $[0,T]\times B_M$, we have

$$\begin{aligned} \big \Vert \psi _k - S\big \Vert _{ L^1({\mathbb {R}}_+\times {\mathbb {R}}^2)}\le & {} (\pi M^2 T)^{1-\frac{1}{2q}} \big \Vert \psi _k - S\big \Vert _{ L^{2q}({\mathbb {R}}_+\times {\mathbb {R}}^2)} \\\le & {} (\pi M^2)^{1-\frac{1}{2q}} T^\frac{1}{2} \big \Vert \psi _k - S\big \Vert _{ L^2({\mathbb {R}}_+; L^{2q}({\mathbb {R}}^2))}, \end{aligned}$$

from which we deduce that $(\psi _k)_{k\ge 1}$ converge in $L^1([0,T] \times B_M)$ to *S*. Thus ${\mathcal {F}} \psi _k(\tau , \xi )$ converges to ${\mathcal {F}} S(\tau ,\xi )$ for all $(\tau ,\xi )$ and the convergence is uniform. As a consequence, ${\mathcal {F}}S(\tau ,\xi )=0$ for $\mu _0 \otimes \mu$-almost all $(\tau ,\xi ) \in {\mathbb {R}}_+ \times {\mathbb {R}}^d$ and by Hypothesis $\mathbf{(H2)}$, we obtain ${\mathcal {F}}S(\tau ,\xi )=0$ for almost all $(\tau ,\xi ) \in {\mathbb {R}}_+ \times {\mathbb {R}}^d$ with respect to the Lebesgue measure.

Hence $S(t,x)=0$ for almost all $t>0$ and $x \in {\mathbb {R}}^d$, i.e. there exists a Borel set $N \subset {\mathbb {R}}_{+} \times {\mathbb {R}}^d$ with $\lambda _{d+1}(N)=0$ such that $S(t,x)=0$ for all $(t,x) \not \in N$. Here $\lambda _{k}$ denotes the Lebesgue measure on ${\mathbb {R}}^{k}$. Therefore,

$$\begin{aligned} I=\int _0^{\infty }\int _{{\mathbb {R}}^d}\int _{{\mathbb {R}}^d} 1_{A}(t,x,y) S(t,x) S(t,y)\gamma (x-y)dxdydt, \end{aligned}$$

where $$A:=\{(t,x,y) \in {\mathbb {R}}_{+} \times {\mathbb {R}}^d \times {\mathbb {R}}^d; (t,x) \in N,(t,y) \in N\}$$.

Let $N_t=\{x \in {\mathbb {R}}^d; (t,x) \in N\}$ be the section of the set *N* at point $t>0$. By Fubini’s theorem, $\lambda _{d+1}(N)=\int _{0}^{\infty }\lambda _d(N_t)dt$. Since $\lambda _{d+1}(N)=0$, we infer that $\lambda _d(N_t)=0$ for almost all $t>0$. Note that the section of the set *A* at point *t* is $A_t=\{(x,y) \in {\mathbb {R}}^d \times {\mathbb {R}}^d; (t,x,y) \in A\}=N_t \times N_t$, and its Lebesque measure is $\lambda _{2d}(A_t)=\lambda _{d}^2(N_t)=0$ for almost all $t>0$. By applying Fubini again, we infer that $\lambda _{2d+1}(A)=\int _0^{\infty }\lambda _{2d}(A_t)dt=0$. This shows $I=0$, which contradicts (A.1). $\square$

### Proof of Proposition 1.9

In this section, we only sketch the proof of Proposition 1.9 as the main body of the proof is almost identical to that in [42, Proposition 3.2].

#### Proof of (1.27)

Using the duality relation (2.5) and the identity $L = -\delta D$, we have

$$\begin{aligned}&{\mathbb {E}}\big [ \langle DF, - DL^{-1}G \rangle _{\mathcal {H}} \big ] \\&\quad = {\mathbb {E}}\big [ F (- \delta D)L^{-1}G \big ] = {\mathbb {E}}[ F LL^{-1}G] ={\mathbb {E}}[FG]= \text {Cov}(F,G), \end{aligned}$$

which shows the equality in (1.27). Then, applying the Gaussian Poincaré inequality (2.12) and using Lemma 3.2 of [26], we can bound the variance appearing in the left-hand side of (1.27) by

$$\begin{aligned} {\mathbb {E}}\Big [ \Vert D \langle DF, - DL^{-1}G \rangle _{\mathcal {H}} \Vert _{\mathcal {H}}^2 \Big ]&\le 2 {\mathbb {E}}\Big [ \Vert \langle D^2F, - DL^{-1}G \rangle _{\mathcal {H}} \Vert _{\mathcal {H}}^2 \Big ] \\&\quad + 2 {\mathbb {E}}\Big [ \Vert \langle DF, - D^2L^{-1}G \rangle _{\mathcal {H}} \Vert _{\mathcal {H}}^2 \Big ]. \end{aligned}$$

We will show that the first expectation-term is bounded by $A_1$ and the other one can be estimated in the same way and bounded by $A_2$. Using the representation (see *e.g.* [25, Proposition 2.9.3])

$$\begin{aligned} -DL^{-1} G = \int _0^\infty dt e^{-t} P_t DG, \end{aligned}$$

with $\{P_t, t\ge 0\}$ the Ornstein-Uhlenbeck semigroup, we can write

6.2 $$\begin{aligned} \langle D^2F, - DL^{-1}G \rangle _{\mathcal {H}} = \int _0^\infty dt e^{-t} \langle D^2F, P_t DG \rangle _{\mathcal {H}}. \end{aligned}$$

Note that if $({\mathcal {M}}, {\mathfrak {M}}, \nu )$ is a probability space on which $s\in {\mathcal {M}}\longmapsto V_s\in |{\mathcal {H}}|$ is ${\mathfrak {M}}$-measurable such that $\int _{{\mathcal {M}}} \big \Vert |V_s|\big \Vert _{{\mathcal {H}}}^2 \nu (ds)<\infty$, then by Fubini’s theorem and Cauchy-Schwarz inequality,

$$\begin{aligned} \left\| \int _{{\mathcal {M}}} V_s \nu (ds) \right\| _{{\mathcal {H}}}^2&= \int _{{\mathcal {M}}^2} \langle V_s, V_{s'}\rangle _{{\mathcal {H}}} \nu (ds) \nu (ds') \\&\le \int _{{\mathcal {M}}^2} \frac{\Vert V_s\Vert ^2_{{\mathcal {H}}} +\Vert V_{s'}\Vert ^2_{{\mathcal {H}}} }{2} \nu (ds) \nu (ds') =\int _{{\mathcal {M}}} \Vert V_s\Vert _{{\mathcal {H}}}^2 \nu (ds). \end{aligned}$$

Using the above inequality on $({\mathbb {R}}_+, e^{-t}dt)$, we deduce from (6.2) that

$$\begin{aligned} \big \Vert \langle D^2F, - DL^{-1}G \rangle _{\mathcal {H}} \big \Vert _{{\mathcal {H}}}^2 \le \int _0^\infty dt e^{-t} \big \Vert \langle D^2F, P_t DG \rangle _{\mathcal {H}} \big \Vert ^2_{{\mathcal {H}}}. \end{aligned}$$

Observe that $\langle D^2F, P_t DG \rangle _{\mathcal {H}}$ is nothing else but the one-contraction $D^2F\otimes _1 P_tDG$, so that

$$\begin{aligned} \big \Vert \langle D^2F, P_t DG \rangle _{\mathcal {H}} \big \Vert ^2_{{\mathcal {H}}}&= \langle D^2F\otimes _1 P_tDG, D^2F\otimes _1 P_tDG \rangle _{{\mathcal {H}}} \\&= \big \langle D^2F\otimes _1 D^2F, (P_tDG)\otimes (P_tDG) \big \rangle _{{\mathcal {H}}^{\otimes 2}}, \end{aligned}$$

where the last equality follows from the definition of contractions. Therefore, we have

$$\begin{aligned}&{\mathbb {E}}[ \Vert \langle D^2F, - DL^{-1}G \rangle _{\mathcal {H}} \Vert _{\mathcal {H}}^2 ] \\&\quad \le {\mathbb {E}}\int _0^\infty dt ~e^{-t} \int _{{\mathbb {R}}_+^6\times {\mathbb {R}}^{6d}} drdr' dsds' d\theta d\theta ' dzdz'\\&\qquad \times dydy' dwdw' \gamma _0(\theta - \theta ') \gamma _0(s-s') \gamma _0(r-r') \gamma (z-z') \gamma (w-w') \gamma (y-y') \\&\qquad \times \big [ D_{r,z}D_{\theta ,w}F \big ] \big [ D_{s,y}D_{\theta ',w'}F \big ] P_t (D_{r',z'}G) P_t(D_{s', y' }G) \end{aligned}$$

and thus we end our estimation of ${\mathbb {E}}[ \Vert \langle D^2F, - DL^{-1}G \rangle _{\mathcal {H}} \Vert _{\mathcal {H}}^2 ]$ by using Hölder inequality and the contraction property of $P_t$ on $L^4(\Omega )$, that is, using $\Vert P_t (D_{r',z'}G) \Vert _4 \le \Vert D_{r',z'}G \Vert _4$.

To estimate the other expectation-term ${\mathbb {E}}[ \Vert \langle DF, - D^2L^{-1}G \rangle _{\mathcal {H}} \Vert _{\mathcal {H}}^2 ]$, one can begin with

$$\begin{aligned} - D^2L^{-1}G = \int _0^\infty dt e^{-2t} P_t D^2G \end{aligned}$$

and then follow the same arguments.

$$\square $$

## Footnotes

1 The spectral measure $\mu$ of $\gamma$ is a tempered measure on ${\mathbb {R}}^d$ such that $\gamma ={\mathcal {F}}\mu$, that is, $\gamma$ is the Fourier transform of $\mu$, and its existence is guaranteed by the Bochner-Schwarz theorem.

2 In higher dimension $(d\ge 3)$, the fundamental wave solution is a uniform measure supported on certain surfaces, then the Malliavin derivative *Du*(*t*, *x*) is expected to be merely a random measure instead of being a random function. In this case, the expression $D_{s,y}u(t,x)$ does not make sense; see also the recent article [34] for related discussions.

3 $p_t(x) =(2\pi t)^{-d/2} e^{-|x|^2/(2t)}$ for $t>0$ and $x\in {\mathbb {R}}^d$; in (1.13), $d=1$.

4 The use of second-order Gaussian Poincaré inequality for obtaining CLT on a Gaussian space is one of the central techniques in the Malliavin-Stein approach; for example, in the recent paper [13], Dunlap *et al.* have used this Poincaré inequality to investigate the Gaussian fluctuation of the KPZ in dimension three and higher. We remark here that we can not directly apply this inequality because of the complicated correlation structure of the underlying Gaussian homogeneous noise, while the underlying Gaussian noise in [13] is white in time and smooth in space so that they can directly apply the version from [26]. In this article, we have established a quite involved variant of second-order Poincaré inequality, which is tailor-made for our applications.

5 Note that there is a typo in equation (5.3.2) of [25]: We have $E[\Vert DF\Vert _{{\mathcal {H}}}^4]^{1/4}$ instead of $E[\Vert D^2F\Vert _{{\mathcal {H}}}^4]^{1/4}$.

6 Unlike in [25], we do not assume *F* to have a density; in fact, it suffices to use [44, Proposition 2.1.1] and [25, (5.1.1)] to establish [25, Eq. (5.1.4)].

7 For the sake of completeness, we sketch a proof of (2.13) here: Given such a function $f \in {\mathcal {H}}_0^{\otimes n}$,

$$\begin{aligned} \Vert f\Vert _{{\mathcal {H}}^{\otimes n}}^2&= \int _{[0,t]^{2n}} d\pmb {s_n} d\pmb {t_n} \big \langle f(\pmb {s_n}, \bullet ), f(\pmb {t_n}, \bullet ) \big \rangle _{{\mathcal {P}}_0^{}\otimes n} \prod _{j=1}^n \gamma _0(s_j-t_j) \\&\le \int _{[0,t]^{2n}} d\pmb {s_n} d\pmb {t_n} \frac{1}{2} \Big ( \big \Vert f(\pmb {s_n}, \bullet )\big \Vert _{{\mathcal {P}}_0^{\otimes n} }^2 + \big \Vert f(\pmb {t_n}, \bullet ) \big \Vert _{{\mathcal {P}}_0^{\otimes n} } ^2 \Big )\prod _{j=1}^n \gamma _0(s_j-t_j) \le \Gamma _t^n \Vert f\Vert _{{\mathcal {H}}_0^{\otimes n}}^2. \end{aligned}$$

8 This means $f = 0$ outside $(J \times {\mathbb {R}}^{d})^p$ and $g= 0$ outside $(J^c\times {\mathbb {R}}^{d})^q$ for some set $J\subset {\mathbb {R}}_+$. We will apply this formula to functions $f=f_{t,x,j}^{(j)}(r,z;\bullet )$ and $g=f_{r,z,n-j}$ given in Sect. 3.1, in which case $J=(r,t)$.

9 We can apply this lemma to the function $y\in {\mathbb {R}}^2\mapsto G_{t-s}(x-y)$ whose support is contained in $\{y \in {\mathbb {R}}^2; |x-y|<t-s\}$, so we can choose $\Lambda = 2t-2s$.

10 The function $\pmb {x_{j-1}} \rightarrow f_{t,x,j}^{(j)}(\pmb {t_{j-1}},\pmb {x_{j-1}})=G_{t-t_1}(x-x_1)G_{t_1-t_2}(x_1-x_2)\ldots G_{t_{j-1}-r}(x_{j-1}-z)$ has support contained in $\{\pmb {x_{j-1}}\in {\mathbb {R}}^{2(j-1)};|x_i-x|<t-t_i, \ \text{ for } \text{ all } \ i=1,\ldots ,j-1\}$.

11 Note that there is a typo in Theorem 6.1.2 of [25]: In (6.1.3) of [25], one has *d*/2 instead of 1/2.
